# Global burden and risk factors of chronic kidney disease due to hypertension in adults aged 20 plus years, 1990–2021

**DOI:** 10.3389/fpubh.2025.1503837

**Published:** 2025-05-07

**Authors:** Yanfei Lai, Haichun Long, Zhonge Liang, Chunxiang Wu, Linghong Wu, Chunxiao Liu, Xiaoyan Meng

**Affiliations:** ^1^Department of Nephrology, The Fourth Affiliated Hospital, Guangxi Medical University, Liuzhou, Guangxi, China; ^2^Linghong Wu - Medical Case Data Center, The Fourth Affiliated Hospital of Guangxi Medical University, Liuzhou, Guangxi, China

**Keywords:** global burden 2021, chronic kidney disease, hypertension, adults, risk factors

## Abstract

**Background:**

The incidence of hypertension-related chronic kidney disease (CKD) is increasing globally annually, and delayed intervention may lead to more complications and an increased disease burden. No study has analyzed the most recent disease burden data for hypertension-associated CKD from 1990 to 2021.

**Methods:**

In this study, by investigating trends in hypertension-related CKD age-standardized incidence, death, and disability-adjusted life year (DALY) rates, and risk factors for death and DALYs among populations aged 20 years and over between 1990 and 2021, the burden of hypertension-induced CKD was estimated globally, regionally, and nationally based on data from the Global Burden of Disease 2021 from 204 countries and areas.

**Results:**

In 2021, there were 1.27 million individuals with hypertension-induced CKD among the population aged 20 years and older globally. Between 1990 and 2021, the number of incident cases globally increased by 63.87%, the number of deaths increased by 67.37%, the corresponding age-standardized incidence rate increased from 15.025 to 24.334 per 100,000 population, and the age-standardized death rate increased from 4.811 to 8.628 per 100,000 population. The age-standardized death and DALY rates remained stable in the low sociodemographic index (SDI) regions, and most of the medium- and high-SDI regions experienced an increasing trend between 1990 and 2021. Among the 204 countries, Seychelles had the highest age-standardized death and DALY rates. Kidney dysfunction, high fasting plasma glucose levels, dietary risks, high body mass index, and high systolic blood pressure are key risk factors for mortality and DALYs in 2021.

**Conclusion:**

The global burden of hypertension-induced CKD has risen rapidly for the last 32 years and will continue to increase in the future.

## Introduction

Hypertension, a global health issue affecting over 1 billion adults worldwide, is a significant contributor to renal failure, resulting in early mortality and functional impairment (1–3). Research findings indicate that hypertension is emerging as a significant contributor to the onset of chronic kidney disease (CKD) in various income brackets worldwide (4). In 2000, over a quarter of the mature population showed signs of elevated blood pressure, with this rate forecasted to rise significantly, reaching 60% by 2025 (5). Increasing trends in fatalities and disease burden are linked to inadequate blood pressure management (1, 6). Despite advancements in its recognition and management, the prevalence of hypertension continues to increase. The National Health and Nutrition Examination Survey data spanning 2011 to 2014 indicated that almost half (47%) of individuals aged 20 years and above in the United States with elevated blood pressure exhibited unregulated elevated blood pressure (1).

CKD is defined as kidney structural or functional abnormalities persisting ≥3 months due to various causes (7). Hypertensive renal arteriolar sclerosis is the primary cause of CKD (8). From 1990 to 2019, there was an increase in the global incidence rate of hypertension-induced CKD, with regions with high sociodemographic index (SDI) exhibiting the highest age-standardized incidence rate (ASIR), whereas regions with low SDI had the highest age-standardized death rate (ASDR) as opposed to high-SDI regions (9). The incidence of cardiovascular disease in individuals with CKD is notably elevated and seems to pose a standalone risk for predicting cardiovascular disease outcomes, especially in high-risk populations (10). CKD contributes significantly to the increased mortality risk associated with cardiovascular disease and serves as a confounding factor in individuals with elevated blood pressure (11–14).

Moreover, patients with CKD have a high demand for healthcare resources, and there is a projected increase in the number of individuals with uremia (11). Hypertension-related CKD is associated with a higher mortality rate in patients with uremia (15). The study of disease burden and quality of life in patients with hypertension-related CKD is highly important for both patients and society.

This comprehensive study delved into the global impact of CKD resulting from hypertension in adults aged 20 years and older, along with its causes, using data from Global Burden of Disease (GBD) research conducted between 1990 and 2021. Variances observed in different countries and regions and the time trend of the incidence, mortality, and disability-adjusted life years (DALYs) caused by CKD are linked to hypertension, exploring the association between various SDI regions and the occurrence, fatality rate, and burden of disease in relation to hypertension-induced CKD, and offering suggestions for enhancing the quality of life among individuals with hypertension-induced CKD.

## Methods

### Data collection

Data for 371 diseases and injuries across 204 countries and areas from 1990 to 2021 were gathered from vital registration systems, verbal autopsy, census, family surveys, registries specific to various diseases and health conditions, and data on health service contacts, gathered from various sources, including records from different sources (16). Diseases were defined according to standardized criteria, and data regarding the incidence, death, and DALYs of hypertension-induced CKD were gathered for individuals over the age of 20 years using the Global Health Data Exchange query tool^1^ established by GBD collaborators (16). A detailed research methodology of GBD has been published in previous studies (16). We extracted the data on hypertension-related CKD and its risk factors from 1990 to 2021 for further analysis. The study population was segmented into 13 categories, including those aged 20–24, 25–29, 30–34, 35–39, 40–44, 45–49, 50–54, 55–59, 60–64, 65–69, 70–74, 75–79, and 80 years and over. In our research, individuals aged 20 years and above were examined for data regarding the incidence, deaths, and DALYs that were gathered by us, and the risk factors at level 2 that contributed to DALYs and deaths, along with the corresponding rates globally, in 21 geographical regions, and in 204 national levels.

### SDI

The SDI serves as a comprehensive measure of the development status of a specific country or region and is based on a comprehensive assessment of the total fertility rate of women under 25, educational attainment of women aged 15 and older, and income per capita. The SDI was divided into five levels. This study investigated the correlation between the burden of CKD caused by hypertension in individuals aged ≥ 20 years and socioeconomic development in five SDI regions.

### DALYs

DALYs quantify the cumulative impact on both mortality and morbidity, reflecting the years of healthy life lost due to premature death and disability from disease. DALY represents the measurement of life both in terms of quantity and quality and is the most widely used and representative index for evaluating and measuring the economic burden of disease (17–19).

### Statistical analysis

We calculated the incidence, death, and DALYs cases and their corresponding rates (per 100,000 population), along with the 95% uncertainty interval (UI) according to the GBD algorithm (20). Age-standardized rates were used for global comparisons across regions, over time, and across populations to analyze the burden of disease among the groups. The trend in disease burden was investigated by analyzing the dynamics of CKD linked to hypertension in individuals aged ≥ 20 years, using estimated annual percentage changes (EAPCs). The value of EAPC was calculated using the formula (21). An EAPC of less than 0 indicates a decreasing trend in the rate over time, and a value greater than 0 indicates an increasing trend. The 95% confidence intervals (CIs) for EAPCs were determined using a linear model. Calculating the estimated average percentage change (EAPC) by fitting the natural logarithm of the age-standardized rate (ASR) to the year to depict long-term trends in the ASR of the disease burden, Y = *α* + *β*X + *ε*, where y = specific value of ln (ratio), x = year, and ε = error. Therefore, the EAPC and its 95% confidence interval (CI) were calculated as 100 × (exp (β) −1). Additionally, risk factors for mortality and DALYs were assessed. Statistical analyses were performed using R software (version 4.2.2). *p* < 0.05 was considered statistically significant.

## Results

### Global trends

#### Incidence

As shown in Supplementary Table S1, globally, the incident cases of hypertension-induced CKD in populations aged ≥20 years reached 1.27 million (95% UI, 1.19–1.36) in 2021. Between 1990 and 2021, the number of incident cases increased by 63.87% globally (95% UI, 1.631–1.907). The corresponding ASIR increased accordingly from 15.025 (95% UI, 13.779–16.393) in 1990 to 24.334 (95% UI, 22.691–25.931) per 100,000 population in 2021, and the EAPC was 1.572 (95% CI, 1.511–1.633). Between 1990 and 2021, the ASIR of the disease increased in adults aged ≥20 years; the largest increase (28.89%) occurred in adults aged 75–79 years, and the smallest increase (13.05%) occurred in adults aged 25–29 years (Supplementary Figure 1A). The highest ASIRs were reported in populations over 80 years of age among the different age groups in 1990 and 2021 (30.0 and 28.3%, respectively) (Supplementary Figures 2A,C). The groups with the highest numbers of incidents in 1990 and 2021 were adults aged ≥80 years (81167.365 and 261749.974, respectively) (Supplementary Figure 1D). Supplementary Figure 3 illustrates the numbers of incident and age-specific rates (per 100,000 person-years) between 1990 and 2021. In 1990, incident cases peaked in females aged 80 + years and in males aged 65–69 years. In 2021, incident cases peaked in females aged 80 + years and males aged 70–74 years; the incident cases in males decreased in the 75–79 years age group and increased again in the 80 + years age group. Unlike incident cases, the incidence rates continue to increase with age in males and females.

#### Mortality

Over the past 32 years, the number of deaths from CKD caused by hypertension increased by 67.37% in 1990 (147950.889, 95% UI: 122218.426–176320.795) and 2021 (453419.544, 95% UI: 380122.953–523419.266) globally. Similarly, the ASDR increased from 4.811 (95% UI: 3.975–5.734) in 1990 to 8.628 (95% UI: 7.233–9.959) per 100,000 population in 2021. The EAPC was 2.020 (95% CI, 1.961–2.078) (Supplementary Table S2). With the exception of a modest decrease (10.60%) among those aged 20–24 years, the mortality rate increased in adults of all ages, increasing much faster in those aged 55–59 years or older; the largest increase in mortality (35.69%) was among adults aged 80 + years (Supplementary Figure 1B). The percentages of different age groups in terms of ASDR in 1990 and 2021 were highest among adults aged 80 + years (46.4 and 52.1%, respectively) (Supplementary Figures 2B,E). The highest numbers of deaths in 1990 and 2021 occurred among adults aged 80 + years (43246.420 and 188815.715, respectively) (Supplementary Figure 1E). In 1990 and 2021, the death cases among adults aged 20–79 years were greater in men than in women; however, among adults aged 80 + years, the cases were greater in women than in men. The mortality rates continued to rise with age for both men and women, and those in the 80 + years group were more rapid (Supplementary Figure 3).

#### DALYs

The global DALYs cases of CKD caused by hypertension increased by 60.47% (4256733.709; 95% UI, 3577599.278–5030814.990) in 1990 vs. 10767724.382 (95% UI, 9142005.282–12240814.073) in 2021. The age-standardized DALY rate increased from 138.428 (95% UI: 116.343–163.601) per 100,000 in 1990 vs. 204.884 (95% UI: 173.951–232.914) per 100,000 in 2021. The EAPC was 1.319 (95% CI, 1.269–1.369) (Supplementary Table S3). Between 1990 and 2021, the number of DALY cases increased in populations older than 20 years in all age groups. The most increase in DALY cases (74.83%) occurred among adults aged 80 + years. The groups with the most DALYs cases in 1990 and 2021 were adults aged 80 + years (552883.602 and 2196447.853, respectively) (Supplementary Figure 1F). With the exception of a modest decrease (9.25%) among those aged 20–24 years, the age-standardized DALY rate increased in adults of all ages, and those in the 55–59 years and over groups increased more rapidly; the greatest increase in the age-standardized DALY rate (29.32%) occurred in adults aged 80 + years (Supplementary Figure 1C). Adults aged 80 + years had the highest age-standardized DALY rate component ratios for different age groups in 1990 and 2021 (28.4 and 32.0%, respectively) (Supplementary Figures 2C,F). In 1990 and 2021, the number of DALYs among adults aged 20–79 years was greater in men than in women; however, among adults aged 80 + years, the number was greater in women than in men. The age-standardized DALY rate followed the same trend as the mortality rate (Supplementary Figure 3).

### SDI regional trends

#### Incidence

In 1990 and 2021, the highest number of cases occurred in the high-SDI region (190828.459, 95% UI: 175524.915–209857.592; 410258.895, 95% UI: 380657.395–438324.532, respectively); in 1990, females had the highest number of cases, but by 2021, males had the highest number of cases. In 1990 and 2021, the largest increase (72.76%) in incident cases occurred in the middle-SDI region, similarly, the increase in the number of cases was largest among males and females. The maximum increase (51.71%) in ASIR occurred in the middle-SDI region (EAPC, 2.446; 95% CI, 2.360–2.532). Similarly, the increase in ASIR in males and females was the largest among the five SDI regions (Table 1; Supplementary Table S1; and Supplementary Figure 4A).

**Table 1:** Sex-specific incidence of chronic kidney disease due to hypertension between 1990 and 2021 at the global and regional level.

	Rate per 100,000 (95% UI)					
	1990		2021		1990–2021	
Location	Incident cases	ASIR	Incident cases	ASIR	Cases change	EAPC (95%CI)
Global						
Male	227688.474 (207881.051, 247882.345)	14.898 (13.602, 16.219)	646854.536 (603639.347, 689845.784)	24.870 (23.209, 26.523)	1.841 (1.699–1.992)	1.679 (1.624, 1.733)
Female	234323.480 (215058.974, 256417.984)	15.150 (13.905, 16.579)	632020.653 (590440.096, 671319.794)	23.808 (22.242, 25.289)	1.697 (1.566–1.838)	1.465 (1.397, 1.534)
High SDI						
Male	87052.223 (79749.936, 95489.084)	28.594 (26.196, 31.365)	208082.323 (193558.042, 222176.784)	48.841 (45.432, 52.149)	1.39 (1.262–1.525)	1.680 (1.612, 1.748)
Female	103776.236 (95404.157, 114290.868)	32.052 (29.466, 35.300)	202176.572 (186312.097, 216374.231)	46.447 (42.802, 49.709)	0.948 (0.838–1.068)	1.175 (1.126, 1.224)
High middle SDI						
Male	46139.277 (41828.791, 50613.934)	13.613 (12.341, 14.933)	127329.331 (117371.019, 137080.287)	25.796 (23.779, 27.772)	1.760 (1.603–1.934)	2.170 (2.099, 2.241)
Female	49418.967 (45255.351, 54411.056)	13.943 (12.769, 15.352)	134020.640 (124269.549, 143677.233)	26.430 (24.507, 28.335)	1.712 (1.556–1.889)	2.125 (2.047, 2.204)
Middle SDI						
Male	54258.371 (49042.641, 59642.130)	22026.109 (20030.120, 23938.216)	192453.196 (178351.564, 205540.486)	22.811 (21.140, 24.362)	2.547 (2.317–2.8)	2.419 (2.344, 2.493)
Female	49718.123 (44990.553, 54839.182)	10.499 (9.501, 11.581)	189333.483 (175685.130, 201791.241)	22.127 (20.532, 23.583)	2.808 (2.521–3.094)	2.480 (2.380, 2.580)
Low middle SDI						
Male	31147.214 (28299.371, 34294.457)	10.794 (9.807, 11.884)	93751.703 (86020.783, 101514.033)	16.360 (15.011, 17.714)	2.010 (1.871–2.165)	1.230 (1.145, 1.315)
Female	24156.679 (21815.671, 26709.198)	8.572 (7.741, 9.478)	83874.020 (77065.058, 91234.833)	14.370 (13.204, 15.632)	2.472 (2.305–2.646)	1.557 (1.473, 1.641)
Low SDI						
Male	8883.663 (7980.939, 9734.798)	8.066 (7.246, 8.838)	24649.387 (22377.927, 26760.084)	9.394 (8.528, 10.198)	1.775 (1.633–1.946)	0.427 (0.306, 0.548)
Female	7043.101 (6341.693, 7787.796)	6.312 (5.683, 6.979)	22026.109 (20030.120, 23938.216)	8.135 (7.398, 8.841)	2.127 (1.951–2.311)	0.738 (0.605, 0.870)

#### Mortality

Only the low-SDI region exhibited a decrease (7.89%) in ASDR, and the largest increase in ASDR (65.24%) occurred in the high-SDI region; the same trend was observed when the total ASDR was divided into males and females. In 2021, the region with a high SDI had the highest ASDR (11.881, 95% UI: 9.471–13.794), and the lowest ASDR was in the low SDI region (6.328, 95% UI: 5.112–7.738). The lowest EAPC was in the low-SDI region (−0.335, 95% UI: −0.455 to −0.214). The middle-SDI region had the highest number of death cases in 1990 and 2021 (53798.019, 95% UI: 44546.776–64030.977; 169362.713, 95% UI: 139634.165–197779.088, respectively). In 1990 and 2021, the lowest number of deaths occurred in the low-SDI regions (15231.631, 95% UI: 12089.308–18855.459; 33738.021, 95% UI: 27253.680–41255.771, respectively) (Table 2; Supplementary Table S2; Supplementary Figure 4B).

**Table 2:** Sex-specific mortality of chronic kidney disease due to hypertension between 1990 and 2021 at the global and regional level.

	Rate per 100,000 (95% UI)					
	1990		2021		1990–2021	
Location	Deaths cases	ASDR	Deaths cases	ASDR	Cases change	EAPC (95%CI)
Global						
Male	80107.356 (64998.448, 99670.033)	5.241 (4.253, 6.521)	239260.222 (199603.268, 279808.588)	9.199 (7.674, 10.758)	1.987 (1.409–2.376)	1.517 (1.369, 1.665)
Female	67843.533 (55965.721, 80582.158)	4.386 (3.618, 5.210)	214159.322 (175088.101, 249118.059)	8.067 (6.596, 9.384)	2.157 (1.710–2.499)	1.641 (1.488, 1.794)
High SDI						
Male	12329.655 (10249.363, 14615.246)	4.050 (3.367, 4.801)	50280.527 (42446.848, 57433.228)	11.802 (9.963, 13.481)	3.078 (2.681–3.568)	3.110 (2.817, 3.404)
Female	13616.161 (10797.995, 16531.837)	4.205 (3.335, 5.106)	52049.482 (38744.597, 61676.220)	11.958 (8.901, 14.169)	2.823 (2.382–3.336)	3.269 (3.033, 3.505)
High middle SDI						
Male	13222.358 (10638.728, 17105.288)	3.901 (3.139, 5.047)	33042.634 (26083.869, 40952.595)	6.694 (5.284, 8.297)	1.499 (0.885–1.973)	1.379 (1.215, 1.544)
Female	11561.441 (9153.189, 13991.708)	3.262 (2.583, 3.948)	31643.806 (24407.219, 38921.035)	6.241 (4.813, 7.676)	1.737 (1.297–2.181)	1.642 (1.450, 1.834)
Middle SDI						
Male	28861.039 (23368.217, 35987.272)	5.953 (4.820, 7.423)	90225.278 (72342.979, 107792.962)	10.694 (8.575, 12.776)	2.126 (1.388–2.629)	1.507 (1.335, 1.679)
Female	24936.981 (20174.380, 29847.392)	5.266 (4.260, 6.303)	79137.435 (64905.385, 92924.016)	9.249 (7.585, 10.860)	2.173 (1.589–2.651)	1.274 (1.085, 1.464)
Low middle SDI						
Male	16161.826 (12425.675, 22145.837)	5.601 (4.306, 7.674)	45845.628 (36317.843, 55735.392)	8.000 (6.337, 9.726)	1.837 (1.015–2.463)	0.938 (0.864, 1.013)
Female	11886.957 (9490.534, 15057.089)	4.218 (3.368, 5.343)	37055.325 (30153.767, 43692.171)	6.349 (5.166, 7.486)	2.117 (1.421–2.675)	1.125 (1.057, 1.193)
Low SDI						
Male	9450.791 (7035.700, 12409.416)	8.581 (6.388, 11.267)	19647.728 (15542.919, 25303.033)	7.487 (5.923, 9.643)	1.079 (0.787–1.451)	−0.434(−0.495, −0.373)
Female	5780.840 (4562.037, 7358.664)	5.181 (4.088, 6.595)	14090.292 (11407.009, 17322.753)	5.204 (4.213, 6.398)	1.437 (0.977–1.815)	−0.181(−0.270, −0.093)

#### DALYs

Like the mortality rate, only the low-SDI region presented a decrease (10.51%) in the age-standardized DALY rate, and the greatest increase (55.35%) occurred in regions with a high SDI; the same trend was observed when the total age-standardized DALY rate was divided into male and female. The highest number of DALY cases occurred in regions with medium SDI in 1990 and 2021 (1656473.928, 95% UI: 1387826.491–1958501.033; 4301234.444, 95% UI: 3575028.872–4962368.909, respectively), and the largest increase (67.43%) was in the high-SDI region (Table 3; Supplementary Table S3; Supplementary Figure 4C).

**Table 3:** Sex-specific DALYs of chronic kidney disease due to hypertension between 1990 and 2021 at the global and regional level.

	Rate per 100,000 (95% UI)					
	1990		2021		1990–2021	
Location	DALYs cases	Age-standardized DALY rate	DALYs cases	Age-standardized DALY rate	Cases change	EAPC (95%CI)
Global						
Male	2380618.591 (1984600.605, 2910628.702)	155.762 (129.851, 190.440)	5987010.953 (5015843.275, 6967032.304)	230.190 (192.851, 267.870)	1.515 (1.110–1.797)	1.335 (1.289, 1.381)
Female	1876115.117 (1573716.145, 2190945.322)	121.299 (101.748, 141.655)	4780713.429 (4059584.788, 5484673.235)	180.091 (152.925, 206.609)	1.548 (1.236–1.812)	1.304 (1.246, 1.363)
High SDI						
Male	302625.054 (258235.626, 345437.979)	99.404 (84.823, 113.466)	961197.484 (847484.396, 1066431.366)	225.612 (198.921, 250.313)	2.176 (1.919–2.427)	3.076 (2.929, 3.223)
Female	281244.929 (238408.793, 323132.898)	86.865 (73.635, 99.802)	831661.044 (699572.795, 943414.334)	191.061 (160.716, 216.735)	1.957 (1.685–2.223)	2.950 (2.815, 3.084)
High middle SDI						
Male	389429.248 (323363.279, 486161.760)	114.895 (95.404, 143.435)	772966.901 (638814.619, 903696.192)	156.598 (129.419, 183.083)	0.985 (0.580–1.328)	1.033 (0.958, 1.109)
Female	317276.599 (262781.419, 375607.392)	89.519 (74.143, 105.977)	661522.123 (543086.150, 776846.479)	130.460 (107.103, 153.203)	1.085 (0.804–1.371)	1.240 (1.155, 1.325)
Middle SDI						
Male	916787.842 (751628.227, 1121930.313)	189.102 (155.036, 231.416)	2392950.351 (1913830.994, 2833184.472)	283.630 (226.841, 335.810)	1.610 (1.113–2.006)	1.380 (1.338, 1.421)
Female	739686.086 (616614.996, 863486.044)	156.203 (130.213, 182.346)	1908284.093 (1594063.822, 2225726.456)	223.017 (186.295, 260.116)	1.580 (1.194–1.931)	1.114 (1.039, 1.189)
Low middle SDI						
Male	506354.438 (400095.994, 650241.501)	175.468 (138.646, 225.330)	1309893.634 (1057805.411, 1583748.855)	228.575 (184.586, 276.363)	1.587 (0.983–2.071)	0.824 (0.781, 0.866)
Female	372642.953 (304936.531, 465919.158)	132.230 (108.205, 165.329)	1001432.410 (815945.002, 1183675.584)	171.579 (139.799, 202.804)	1.687 (1.163–2.131)	0.772 (0.709, 0.834)
Low SDI						
Male	263069.226 (202352.655, 335274.875)	238.848 (183.721, 304.405)	544461.688 (429064.105, 716276.194)	207.486 (163.510, 272.962)	1.070 (0.791–1.404)	−0.543(−0.639, −0.447)
Female	163602.381 (131781.923, 201254.081)	146.619 (118.102, 180.362)	373752.735 (292419.827, 451340.105)	138.034 (107.996, 166.689)	1.285 (0.881–1.623)	−0.343(−0.489, −0.196)

### Geographic regional trends

#### Incidence

Among the 21 geographic regions, East Asia had the highest number of incident cases in 2021 (234048.632, 95% UI: 214661.524–251427.041), whereas Oceania had the fewest (728.701, 95% UI: 645.549–816.448). The highest ASIR occurred in the high-income Asia-Pacific region (61.784, 95% UI: 56.573–66.840). In contrast, the lowest ASIR was observed in Eastern Sub-Saharan Africa (5.845, 95% UI: 5.346–6.344). From 1990 to 2021, Andean Latin America had the most increase in the ASIR (EAPC: 3.392, 95% CI: 3.251–3.534), whereas Eastern Sub-Saharan Africa had the smallest increase (EAPC: 0.355, 95% CI: 0.137–0.574) (Supplementary Table S1). Except for Oceania, Western Sub-Saharan Africa, and Eastern Sub-Saharan Africa in the low-SDI regions remained stable, and the ASIR of most regions significantly increased (Supplementary Figure 5A).

#### Mortality

In 2021, Southeast Asia had the highest number of deaths cases in adults aged 20 + years (79260.470, 95% UI: 65987.291–93019.145). The highest ASDR occurred in High-income North America (21.219, 95% UI: 17.426–23.816). Except for a modest decrease in Central Sub-Saharan Africa, Eastern Sub-Saharan Africa, and Western Sub-Saharan Africa, the ASDR increased in most regions. Oceania had the smallest increase in the ASDR (EAPC, 0.868; 95% CI: 0.780–0.957), whereas the highest increase was in High-income North America (EAPC, 4.403; 95% CI: 4.217–4.589) (Supplementary Table S2). Overall, the ASDR in the low-SDI regions remained stable, whereas, except for Eastern Europe and Central Europe, the regions with middle and high SDI clearly increased from 1990 to 2021 (Supplementary Figure 5B).

#### DALYs

As shown in Supplementary Table S3, in 2021, Southeast Asia had the highest number of DALY cases in adults aged ≥20 years (2228641.026, 95% UI: 1821439.816–2641164.936), whereas Australasia had the lowest number (22498.785, 95% UI: 17984.292–27196.813). The United Republic of Tanzania had the highest age-standardized DALY rate (475.156, 95% UI: 388.339–563.108). Except for a modest decrease in Central Sub-Saharan Africa, Eastern Sub-Saharan Africa, and Western Sub-Saharan Africa, the age-standardized DALY rate increased in most regions. Central Asia had the lowest age-standardized DALY rate (46.143, 95% UI: 36.261–57.513). From 1990 to 2021, Southern Latin America had the lowest decrease in the age-standardized DALY rate (EAPC, 0.443, 95% CI: 0.184–0.702), and the greatest decrease was in high-income North America (EAPC: 3.748, 95% CI: 3.579–3.917). The age-standardized DALY rate of the regions with low SDI remained stable, and most regions with medium and high SDI increased from 1990 to 2021 (Supplementary Figure 5C).

### National trends

#### Incidence

In 2021, among 204 countries, China had the highest number of incident cases (222113.143, 95% UI: 203154.884–239134.886), Japan had the highest ASIR (72.962, 95% UI: 66.388–78.997), and Somalia had the lowest ASIR (4.360, 95% UI: 3.668–5.193). The highest increase in the incidence rate was observed in the Northern Mariana Islands (EAPC, 4.521; 95% CI: 4.157–4.886), and Afghanistan (EAPC, −1.126; 95% CI, −1.424 to −0.826) experienced the largest decrease. In 2021, the global ASIR was 24.334 (95% UI: 22.691–25.931), and age-standardized incidence rates were higher than the global mean in 100 countries and lower than the global mean in 104 countries (Supplementary Table S4; Supplementary Figure 6A).

#### Mortality

In 2021, China had the highest number of death cases (65231.195, 95% UI: 49295.002–82461.552). Seychelles (29.965, 95% UI: 24.101–36.442) had the highest ASDR, whereas Tajikistan (0.095, 95% UI: 0.064–0.141) had the lowest. Ukraine (EAPC, 13.871, 95% CI: 11.805–15.975) had the greatest increase in ASDR, while Ethiopia (EAPC, −2.027, 95% CI: −2.346 to −1.706) had the greatest decrease. The global ASDR in 2021 was 8.628 (95% UI: 7.233–9.959), which was higher than the global mean in 103 countries and lower than the global mean in 101 countries (Supplementary Table S5; Supplementary Figure 6B).

#### DALYs

In 2021, China had the highest number of DALY cases (1622065.500, 95% UI: 1293394.384–1981189.476). Seychelles had the highest age-standardized DALY rate (743.906; 95% UI: 594.438–898.808). Austria (EAPC, 4.906, 95% CI: 4.326–5.489) had the greatest increase in the age-standardized DALY rate, while Ethiopia (EAPC, −2.653, 95% CI: −2.963 to −2.342) and the Maldives (EAPC, −2.639, 95% CI: −2.827 to −2.450) had the greatest decreases. The global age-standardized rate of DALY in 2021 was 204.884 (95% UI: 173.951–232.914), which was higher than the global mean in 102 countries and lower than the global mean in 102 countries (Supplementary Table S6; Supplementary Figure 6C).

#### Risk factors for death and DALYs

**Figure 1:**
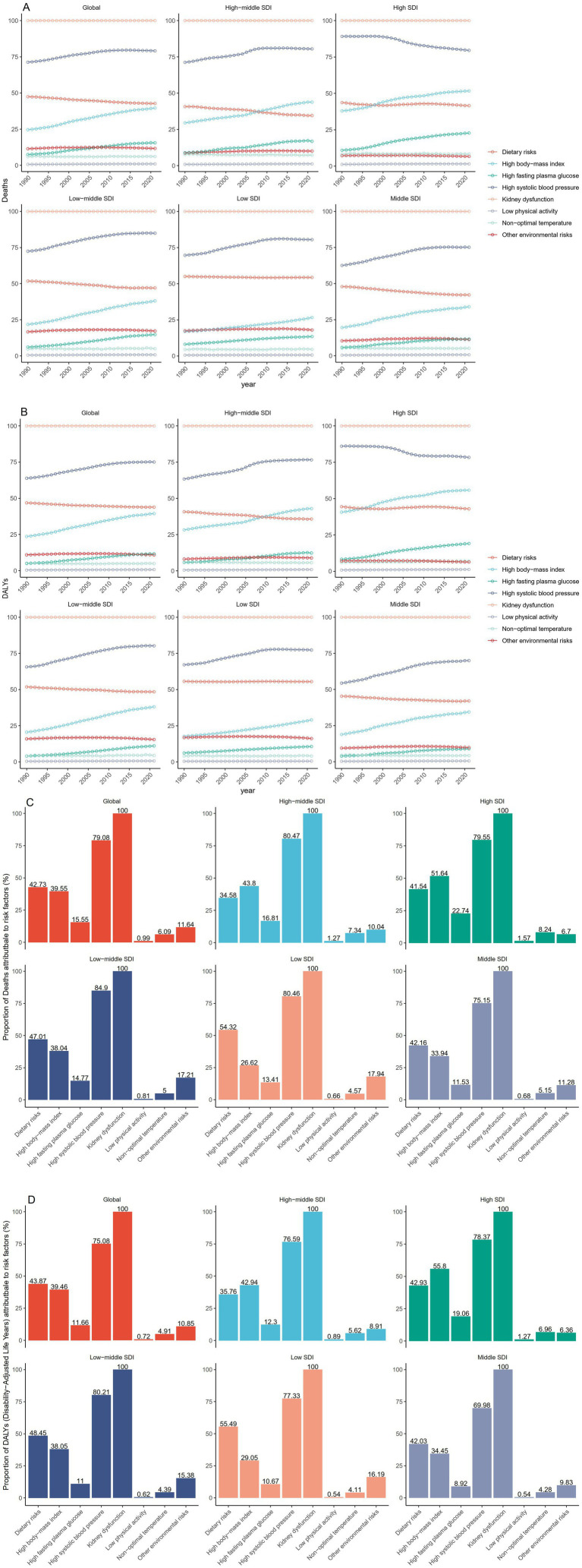
Trends and proportions of deaths and DALYs due to hypertension-induced chronic kidney disease in adults aged 20 years and older attributable to 8 level 2 risk factors, globally and in 5 SDI regions. **(A)** Trends in risk factors for death from 1990 to 2021. **(B)** Trends in risk factors for DALYs from 1990 to 2021. **(C)** Proportion of risk factors for death in 2021. **(D)** Proportion of risk factors for DALYs in 2021. DALYs, Disability adjusted life years. SDI, Sociodemographic Index.

The GBD database identified the following eight level 2 risk factors for death and DALYs of hypertension-related CKD in adults aged ≥20 years: kidney dysfunction, non-optimal temperature, low physical activity, high fasting plasma glucose, dietary risks, high body-mass index (BMI), high systolic blood pressure, and other environmental risks. Overall, in 2021, kidney dysfunction and high systolic blood pressure were the key attributable risk factors globally and in the five SDI regions. In 2021, compared with those in low-SDI regions, deaths from hypertension-related CKD in high-SDI regions were more attributable to kidney dysfunction (100% vs. 100%), high BMI (51.64% vs. 26.62%), high fasting plasma glucose (22.74% vs. 13.41%), non-optimal temperature (8.24% vs. 4.57%), and low physical activity (1.57% vs. 0.66%). In contrast, high systolic blood pressure (80.46% vs. 79.55%), dietary risk (54.32% vs. 41.54%), and other environmental risks (17.94% vs. 6.7%) contributed more to death in countries with a low to high SDI (Figure 1C). The DALYs for hypertension-related CKD in the high-SDI regions were more attributable to kidney dysfunction (100% vs. 100%), high BMI (55.8% vs. 29.05%), high fasting plasma glucose (19.06% vs. 10.67%), high systolic blood pressure (78.37% vs. 77.33%), low physical activity (1.27% vs. 0.54%), and non-optimal temperature (6.96% vs. 4.11%). In contrast, when countries with low to high SDI were compared, dietary risks (55.49% vs. 42.93%) and other environmental risks (16.19% vs. 6.36%) contributed more to DALYs (Figure 1D). The proportion of deaths and DALYs attributed to high BMI globally and in the five SDI regions showed a clear upward trend between 1990 and 2021 and a smaller upward trend in the proportion attributed to high fasting plasma glucose. In addition to the high-SDI regions, there was an increase in the proportion attributed to elevated systolic blood pressure. Globally, and in the five SDI regions, the proportions attributed to kidney dysfunction, dietary risks, non-optimal temperature, low physical activity, and other environmental risks remained stable (Figures 1A,B).

## Discussion

Over the past 32 years, the incidence of hypertension-induced CKD has increased among adults aged ≥20 years worldwide. Our findings provide information about the burden of hypertension-induced CKD for the past 32 years among populations aged 20 years and above in regions and countries with different income levels. Our study reinforces the findings of previous studies conducted between the years 1990 and 2021. Our study may help health managers to develop appropriate prevention and management measures to guide the health management of populations with hypertension-induced CKD.

From 1990 to 2021, the incident, death, and DALY numbers of hypertension-induced CKD in populations aged 20 years and over had increased. The disease burden of hypertension-induced CKD varies greatly between sexes. Overall, the disease burden was greater in males than in females. Especially in the high SDI region, the age-standardized incidence and DALY rates were significantly higher in males than in females. A possible reason is that males are more exposed than females to bad behaviors, such as smoking and drinking, leading to a greater disease burden. The proportion of disease burden in patients of different ages with hypertension-induced CKD differed. Overall, disease burden increased with age. People over 80 years of age have a significantly greater disease burden than those under 80 years of age, indicating that the disease burden in the older adult has increased significantly. The largest increase was among adults aged 75–79 years, possibly because those aged 75–79 years had a long disease duration and more complications as well as malnutrition, anemia, and other adverse health conditions (22). Moreover, the patients undergoing dialysis for CKD were relatively older. A study in the United States indicated that the median age of patients starting dialysis was 65 years (23), which also increases the overall burden of the disease in older adult individuals compared to younger adults. Chen et al. (24) and Ren et al. (25) also reported that the disease burden of hypertension-associated CKD tends to be greater in males and the older adult population. However, it is imperative not to overlook the impact of hypertension-induced CKD in young and middle-aged individuals. Age-standardized mortality and DALY rates increased much faster in those aged 55–59 years and older, which may be mainly related to obesity and poor lifestyle habits. Previous studies have demonstrated a significant association between early-onset hypertension and increased risk of CKD (26, 27), highlighting the need for age-specific prevention and treatment strategies. Our study further identified dietary factors and elevated BMI as significant risk factors for hypertension-related CKD. Global research indicates that high sodium intake shows a strong correlation with both hypertension and CKD risk, with the highest consumption levels observed in East Asia and Eastern Europe (28). Excessive salt consumption may elevate CKD risk (29). In areas with high SDI, it may be mainly related to excessive consumption of processed foods (30). However, the precise mechanisms linking dietary components to hypertension-related CKD require further investigation.

Among the 204 countries, China had the highest number of incident, death, and DALYs cases of hypertension-induced CKD among adults aged ≥20 years, which may be related to the large number of people in China. At present, China is becoming an aging society. Aging is a major factor causing a range of chronic diseases, and the number of patients is increasing annually, which provides an early warning of the disease burden. With the changes in demographic structure, planning for personnel in geriatrics and rehabilitation medicine is inadequate to meet the increasing older adult population, resulting in a lack of medical resources (31, 32). It is necessary to attract the attention of the state and relevant departments and develop relevant measures to address this high burden. The Republic of Seychelles is a middle-income island with the highest hypertension-associated age-standardized mortality and DALY rates linked to CKD. A previous study revealed that in Seychelles, the prevalence rates of hypertension exceeded those observed in the majority of the populations analyzed by Addo et al. (33). Moreover, only 26% of the patients with newly diagnosed high blood pressure maintained good medication adherence over a one-year period (34). The Seychelle population needs to improve their awareness of chronic disease control among patients with hypertension-related CKD. Our study revealed that Austria had the largest increase in age-standardized DALY rate, which may cause a serious disease burden in the country. Several studies have shown that Austrian patients with hypertension are unaware of their blood pressure levels, the damage caused by high blood pressure, and the benefits of controlling it (35, 36). Therefore, most countries should improve health management awareness of the chronic disease population and reduce complications and national disease burden.

The etiology of hypertension-related CKD involves a genetic predisposition and behavioral, environmental, and metabolic factors. The exact role of each factor in hypertension-related CKD needs to be further explored and may vary according to the geographic location. Globally, eight level 2 risk factors contribute to the disease burden of hypertension-induced CKD, one of which is kidney dysfunction, which is the key cause of mortality in populations with hypertension-induced CKD. According to KDIGO guidelines (37), the clinical staging of CKD is classified into five stages. Patients with end-stage renal disease (ESRD) require dialysis or kidney transplantation to survive. ESRD populations have more complications, significantly increased mortality, and poor prognosis (38). Therefore, even in populations with hypertension-related CKD who have already developed the condition, it remains essential to control disease progression to prevent rapid deterioration to ESRD. Despite possessing advanced healthcare systems, high-SDI countries continue to demonstrate increasing age-standardized DALYs and mortality rates related to hypertension-related CKD. High-income North America currently exhibits the highest ASDRs for hypertension-related CKD. While Central, Eastern, and Western Sub-Saharan Africa have shown modest decreases in ASDRs, most other regions have experienced increases. This disparity appears primarily attributable to suboptimal lifestyle factors prevalent in high-SDI regions. In 2021, compared with those in low-SDI regions, deaths and DALYs from hypertension-related CKD in high-SDI regions were more attributable to kidney dysfunction, high BMI, high fasting plasma glucose, non-optimal temperature, and low physical activity. Changes in modern lifestyles, including high BMI, elevated fasting plasma glucose, and elevated systolic blood pressure, have led to rapid increases in patients with metabolic syndrome. A key treatment approach for metabolic syndrome involves adapting one’s lifestyle, such as following a nutritious diet and engaging in regular physical activity (39). Therefore, aggressive sports and dietary management can help mitigate hypertension-related CKD progression. Early identification and treatment of high-risk patients can reduce mortality and DALY rates in patients with hypertension-associated CKD, and help reduce the disease burden.

The study has several limitations. First, the data in the study were obtained from the GBD database, which is affected by the accuracy of the national registration information, and the number of patients with undiagnosed hypertension-related CKD and other uncounted risk factors may have been missed. Second, the study analyzed only the disease burden among adults aged ≥20 years and not among children; our study had certain age limitations. Third, the study analyzed only global, regional, and national disease burdens, and there was a lack of data on the domestic population burden of disease. Fourth, the study did not use the Bayesian age-period cohort to analyze and predict disease burden trends.

## Conclusion

The global burden of CKD caused by hypertension in adults has gradually increased over the past 32 years and is expected to continue to increase. Our findings suggest that early detection through health education can reduce the disease burden by decreasing mortality and disability in patients with hypertension-induced CKD. To delay the progression of hypertension-related CKD, the disease burden of hypertension-induced CKD should be reduced by controlling hypertension, hyperglycemia, and high BMI. Owing to the differences in risk factors for disease burden between men and women, different prevention and control measures should be developed according to sex differences. The burden of disease in the older adult population is high; therefore, we need to pay more attention to geriatric populations with hypertension-related CKD to reduce complication and death rates.

## Data Availability

The original contributions presented in the study are included in the article/Supplementary material, further inquiries can be directed to the corresponding authors.

## Footnotes

^1^ https://vizhub.healthdata.org/gbd-results/
