# Supplementary material for: Global burden and risk factors of chronic kidney disease due to hypertension in adults aged 20 plus years, 1990–2021
Source: Front Public Health. 2025 May 7;13:1503837. doi: 10.3389/fpubh.2025.1503837 (PMC12092432; doi:10.3389/fpubh.2025.1503837)
Supplement: Supplementary file 1 [file Data_Sheet_1.docx]

Supplementary Table S1. Incidence of chronic kidney disease due to hypertension between 1990 and 2021 at the global and regional level.

|  | Rate per 100 000 (95% UI) | | | |  |  |
| --- | --- | --- | --- | --- | --- | --- |
|  | 1990 |  | 2021 |  | 1990-2021 |  |
| Location | Incident cases | ASIR | Incident cases | ASIR | Cases change | EAPC (95%CI) |
| Global | 462011.953(423695.599-504090.710) | 15.025(13.779-16.393) | 1278875.189(1192522.079-1362825.761) | 24.334(22.691-25.931) | 1.768(1.631-1.907) | 1.572(1.511-1.633) |
| SDI |  |  |  |  |  |  |
| High | 190828.459(175524.915-209857.592) | 30.376(27.940-33.405) | 410258.895(380657.395-438324.532) | 47.631(44.194-50.890) | 1.150(1.037-1.271) | 1.416(1.357-1.475) |
| High middle | 95558.244(86828.509-104767.760) | 13.782(12.523-15.110) | 261349.971(242341.588-279787.280) | 26.118(24.218-27.960) | 1.735(1.579-1.904) | 2.147(2.073-2.221) |
| Middle SDI | 103976.495(94111.673-114306.420) | 10.849(9.820-11.927) | 381786.679(354516.626-406928.407) | 22.467(20.862-23.946) | 2.672(2.412-2.928) | 2.446(2.360-2.532) |
| Low middle | 55303.893(50131.011-60897.290) | 9.696(8.789-10.676) | 177625.724(163227.798-192705.421) | 15.356(14.111-16.660) | 2.212(2.064-2.372) | 1.371(1.286-1.457) |
| Low SDI | 15926.764(14352.868-17493.046) | 7.183(6.473-7.890) | 46675.496(42451.970-50670.855) | 8.754(7.962-9.504) | 1.931(1.780-2.099) | 0.565(0.438-0.691) |
| Regions |  |  |  |  |  |  |
| Andean Latin America | 1984.530(1739.176-2234.510) | 10.424(9.136-11.738) | 10963.224(9876.613-12112.172) | 25.820(23.261-28.526) | 4.524(4.094-5.005) | 3.392(3.251-3.534) |
| Australasia | 4355.458(3908.574-4792.508) | 31.103(27.912-34.225) | 11752.027(10371.964-13103.461) | 50.180(44.287-55.951) | 1.698(1.485-1.878) | 1.524(1.443-1.604) |
| Caribbean | 2573.049(2318.137-2836.822) | 12.742(11.480-14.048) | 8782.114(8070.724-9536.135) | 27.277(25.067-29.619) | 2.413(2.205-2.643) | 2.664(2.576-2.751) |
| Central Asia | 2712.105(2369.014-3099.966) | 7.188(6.279-8.216) | 8553.953(7622.052-9652.715) | 13.981(12.458-15.776) | 2.154(1.968-2.345) | 2.154(1.915-2.393) |
| Central Europe | 11681.672(10423.938-13042.083) | 13.611(12.146-15.196) | 30953.257(28399.957-34018.754) | 33.752(30.968-37.095) | 1.650(1.454-1.869) | 2.789(2.686-2.892) |
| Central Latin America | 13754.818(12407.834-15199.949) | 16.820(15.173-18.587) | 61426.389(57444.647-65342.770) | 36.625(34.251-38.960) | 3.466(3.101-3.850) | 2.744(2.683-2.805) |
| Central Sub-Saharan Africa | 1172.588(1013.570-1343.541) | 4.891(4.228-5.604) | 4036.317(3532.759-4582.774) | 6.370(5.575-7.232) | 2.442(2.187-2.698) | 0.732(0.509-0.956) |
| East Asia | 76224.476(68001.235-84196.240) | 10.065(8.979-11.118) | 234048.632(214661.524-251427.041) | 20.752(19.033-22.293) | 2.071(1.834-2.342) | 2.496(2.318-2.674) |
| Eastern Europe | 14653.228(13050.325-16453.196) | 9.203(8.197-10.334) | 32802.975(29748.484-35972.626) | 20.425(18.523-22.399) | 1.239(1.092-1.377) | 2.569(2.428-2.710) |
| Eastern Sub-Saharan Africa | 4064.515(3666.737-4513.399) | 5.085(4.588-5.647) | 11602.957(10612.286-12594.247) | 5.845(5.346-6.344) | 1.855(1.676-2.048) | 0.355(0.137-0.574) |
| High-income Asia Pacific | 35961.572(32930.268-39216.109) | 29.224(26.761-31.869) | 95553.642(87494.646-103373.146) | 61.784(56.573-66.840) | 1.657(1.502-1.814) | 2.561(2.496-2.627) |
| High-income North America | 71562.790(64989.718-78685.320) | 35.839(32.547-39.406) | 142808.948(132197.757-153940.100) | 50.891(47.110-54.858) | 0.996(0.885-1.121) | 1.008(0.885-1.130) |
| North Africa and Middle East | 25770.637(23314.526-28425.951) | 15.867(14.355-17.502) | 116476.434(107513.477-125926.723) | 30.136(27.817-32.581) | 3.520(3.270-3.813) | 1.959(1.802-2.116) |
| Oceania | 218.479(191.884-249.769) | 6.863(6.027-7.846) | 728.701(645.549-816.448) | 9.663(8.560-10.827) | 2.335(2.054-2.623) | 1.040(0.932-1.149) |
| South Asia | 50916.475(45934.761-56586.415) | 9.241(8.337-10.270) | 159993.948(145873.890-175304.811) | 13.756(12.542-15.073) | 2.142(1.977-2.334) | 1.096(1.017-1.175) |
| Southeast Asia | 22814.673(20694.779-25258.741) | 9.289(8.426-10.284) | 89609.045(82061.296-97431.784) | 19.105(17.496-20.773) | 2.928(2.685-3.165) | 2.339(2.232-2.447) |
| Southern Latin America | 6435.079(5693.100-7221.146) | 21.337(18.877-23.944) | 17112.173(15293.990-18968.724) | 35.514(31.740-39.367) | 1.659(1.421-1.934) | 1.757(1.637-1.878) |
| Southern Sub-Saharan Africa | 2766.762(2504.292-3053.678) | 10.659(9.648-11.764) | 8293.065(7599.018-9002.799) | 16.911(15.496-18.358) | 1.997(1.848-2.513) | 1.356(1.144-1.568) |
| Tropical Latin America | 10613.138(9543.794-11675.065) | 12.742(11.459-14.017) | 41394.404(38083.958-44547.343) | 25.721(23.664-27.680) | 2.900(2.624-3.234) | 2.249(2.189-2.309) |
| Western Europe | 94960.115(86176.371-105120.609) | 33.195(30.125-36.747) | 171304.389(157872.396-185421.988) | 49.556(45.670-53.640) | 0.804(0.685-0.932) | 1.408(1.371-1.445) |
| Western Sub-Saharan Africa | 6815.792(6177.032-7498.736) | 7.956(7.211-8.754) | 20678.595(18808.190-22397.689) | 9.346(8.501-10.123) | 2.034(1.884-2.195) | 0.479(0.297-0.662) |

ASIR, age-standardized incidence rate; CI, credibility interval. EAPC, estimated annual percentage change. SDI, Sociodemographic Index. UI, uncertainty interval.

Supplementary Table S2. Mortality of chronic kidney disease due to hypertension between 1990 and 2021 at the global and regional level.

|  | Rate per 100 000 (95% UI) | | | |  |  |
| --- | --- | --- | --- | --- | --- | --- |
|  | 1990 |  | 2021 |  | 1990-2021 |  |
| Location | Deaths cases | ASDR | Deaths cases | ASDR | Cases change | EAPC (95%CI) |
| Global | 147950.889(122218.426-176320.795) | 4.811(3.975-5.734) | 453419.544(380122.953-523419.266) | 8.628(7.233-9.959) | 2.065(1.632-2.335) | 2.020(1.961-2.078) |
| SDI |  |  |  |  |  |  |
| High | 25945.816(21193.493-31182.704) | 4.130(3.374-4.964) | 102330.009(81574.312-118809.816) | 11.881(9.471-13.794) | 2.944(2.540-3.424) | 3.973(3.805-4.140) |
| High middle | 24783.800(20188.386-30126.124) | 3.574(2.912-4.345) | 64686.440(52615.758-78070.399) | 6.464(5.258-7.802) | 1.610(1.150-1.981) | 2.021(1.923-2.120) |
| Middle SDI | 53798.019(44546.776-64030.977) | 5.614(4.648-6.681) | 169362.713(139634.165-197779.088) | 9.966(8.217-11.638) | 2.148(1.584-2.500) | 2.021(1.923-2.120) |
| Low middle | 28048.783(22292.891-35457.471) | 4.918(3.908-6.216) | 82900.953(66928.416-99098.653) | 7.167(5.786-8.567) | 1.956(1.312-2.421) | 1.188(1.125-1.252) |
| Low SDI | 15231.631(12089.308-18855.459) | 6.870(5.452-8.504) | 33738.021(27253.680-41255.771) | 6.328(5.112-7.738) | 1.215(0.958-1.497) | -0.335(-0.455--0.214) |
| Regions |  |  |  |  |  |  |
| Andean Latin America | 1722.561(1400.071-2090.341) | 9.048(7.354-10.980) | 7130.896(5335.579-8838.550) | 16.794(12.566-20.816) | 3.140(2.355-4.097) | 2.244(1.909-2.580) |
| Australasia | 370.075(310.887-433.942) | 2.643(2.220-3.099) | 1553.526(1144.360-1999.752) | 6.633(4.886-8.539) | 3.198(2.550-3.820) | 3.524(3.141-3.908) |
| Caribbean | 1353.836(1107.648-1671.870) | 6.704(5.485-8.279) | 4178.607(3398.130-5092.102) | 12.979(10.554-15.816) | 2.086(1.632-2.616) | 2.562(2.428-2.695) |
| Central Asia | 94.724(70.277-127.712) | 0.251(0.186-0.338) | 415.902(306.740-535.871) | 0.680(0.501-0.876) | 3.391(2.590-4.290) | 2.720(2.253-3.189) |
| Central Europe | 2292.657(1854.807-2795.918) | 2.671(2.161-3.258) | 4152.947(3288.309-4959.456) | 4.529(3.586-5.408) | 0.811(0.592-1.051) | 2.719(2.355-3.085) |
| Central Latin America | 4734.421(3851.665-5619.222) | 5.789(4.710-6.871) | 23809.302(18794.794-28664.727) | 14.196(11.206-17.091) | 4.029(3.512-4.597) | 3.480(3.017-3.946) |
| Central Sub-Saharan Africa | 2270.765(1688.973-2955.622) | 9.472(7.045-12.328) | 5866.539(4142.707-7810.456) | 9.258(6.538-12.326) | 1.584(0.930-2.432) | -0.212(-0.332--0.092) |
| East Asia | 29983.975(23969.413-37339.422) | 3.959(3.165-4.931) | 69138.272(52576.756-86891.032) | 6.130(4.662-7.704) | 1.306(0.751-1.890) | 1.361(1.205-1.518) |
| Eastern Europe | 941.514(754.220-1164.884) | 0.591(0.474-0.732) | 2273.346(1750.301-2856.258) | 1.416(1.090-1.778) | 1.415(1.066-1.791) | 2.754(1.978-3.536) |
| Eastern Sub-Saharan Africa | 5420.778(4145.070-6831.953) | 6.782(5.186-8.548) | 10976.513(8530.418-13679.767) | 5.529(4.297-6.891) | 1.025(0.745-1.322) | -0.904(-1.111--0.697) |
| High-income Asia Pacific | 3497.071(2862.473-4246.299) | 2.842(2.326-3.451) | 11331.466(8010.955-15008.673) | 7.327(5.180-9.704) | 2.240(1.707-2.802) | 3.388(3.031-3.747) |
| High-income North America | 12433.628(10166.337-14667.378) | 6.227(5.091-7.345) | 59542.890(48900.762-66831.718) | 21.219(17.426-23.816) | 3.789(3.272-4.427) | 4.403(4.217-4.589) |
| North Africa and Middle East | 14077.925(10473.487-21722.927) | 8.668(6.448-13.375) | 47421.047(37850.688-56472.040) | 12.269(9.793-14.611) | 2.368(1.071-3.212) | 1.353(1.100-1.606) |
| Oceania | 51.750(34.461-72.672) | 1.626(1.082-2.283) | 165.629(125.551-218.969) | 2.196(1.665-2.904) | 2.201(1.243-3.623) | 0.868(0.780-0.957) |
| South Asia | 14204.424(10735.349-17741.362) | 2.578(1.948-3.220) | 43529.123(33220.893-55385.897) | 3.743(2.856-4.762) | 2.064(1.406-2.696) | 1.057(0.900-1.216) |
| Southeast Asia | 25345.286(21305.034-30775.966) | 10.319(8.674-12.531) | 79260.470(65987.291-93019.145) | 16.899(14.069-19.832) | 2.127(1.578-2.632) | 1.577(1.525-1.629) |
| Southern Latin America | 3267.117(2665.694-3876.586) | 10.833(8.839-12.854) | 6772.242(5403.147-8195.467) | 14.055(11.213-17.008) | 1.073(0.877-1.281) | 1.065(0.720-1.410) |
| Southern Sub-Saharan Africa | 1707.963(1423.545-2164.695) | 6.580(5.484-8.339) | 6865.495(5836.582-8200.832) | 14.000(11.902-16.723) | 3.020(2.152-3.603) | 2.051(1.626-2.478) |
| Tropical Latin America | 4630.257(3837.077-5394.735) | 5.559(4.607-6.477) | 15795.775(12753.653-18743.103) | 9.815(7.925-11.646) | 2.411(2.123-2.680) | 1.896(1.747-2.046) |
| Western Europe | 9769.925(7482.831-12394.732) | 3.415(2.616-4.333) | 29752.263(21810.421-37712.383) | 8.607(6.309-10.910) | 2.045(1.617-2.589) | 3.799(3.589-4.009) |
| Western Sub-Saharan Africa | 9780.236(7879.846-11936.597) | 11.417(9.198-13.934) | 23487.296(18926.339-28442.001) | 10.616(8.554-12.855) | 1.402(1.033-1.802) | -0.354(-0.438--0.271) |

ASDR, age-standardized deaths rate; CI, credibility interval. EAPC, estimated annual percentage change. SDI, Sociodemographic Index. UI, uncertainty interval.

Supplementary Table S3. DALYs of chronic kidney disease due to hypertension between 1990 and 2021 at the global and regional level.

|  | Rate per 100 000 (95% UI) | | | |  |  |
| --- | --- | --- | --- | --- | --- | --- |
|  | 1990 |  | 2021 |  | 1990-2021 |  |
| Location | DALYs cases | Age-standardized DALY rate | DALYs cases | Age-standardized DALY rate | Cases change | EAPC (95%CI) |
| Global | 4256733.709(3577599.278-5030814.990) | 138.428(116.343-163.601) | 10767724.382(9142005.282-12240814.073) | 204.884(173.951-232.914) | 1.530(1.216-1.720) | 1.319(1.269-1.369) |
| SDI |  |  |  |  |  |  |
| High | 583869.982(500473.670-667625.074) | 92.941(79.666-106.274) | 1792858.528(1559906.437-2007077.448) | 208.151(181.105-233.022) | 2.071(1.840-2.311) | 3.021(2.887-3.155) |
| High middle | 706705.847(603557.646-846667.172) | 101.924(87.047-122.110) | 1434489.025(1211026.026-1662497.681) | 143.353(121.022-166.139) | 1.030(0.739-1.273) | 1.129(1.050-1.208) |
| Middle SDI | 1656473.928(1387826.491-1958501.033) | 172.846(144.814-204.361) | 4301234.444(3575028.872-4962368.909) | 253.110(210.376-292.015) | 1.597(1.215-1.859) | 1.254(1.200-1.307) |
| Low middle | 878997.391(721250.897-1080851.943) | 154.106(126.450-189.495) | 2311326.045(1885844.669-2758901.317) | 199.816(163.033-238.510) | 1.630(1.180-1.993) | 0.792(0.742-0.842) |
| Low SDI | 426671.607(339894.815-524437.321) | 192.433(153.296-236.527) | 918214.423(736358.210-1129148.393) | 172.216(138.108-211.778) | 1.152(0.916-1.407) | -0.472(-0.588--0.357) |
| Regions |  |  |  |  |  |  |
| Andean Latin America | 39749.988(32424.247-47637.757) | 208.800(170.319-250.234) | 141621.698(105325.778-176079.152) | 333.544(248.060-414.697) | 2.563(1.866-3.341) | 1.595(1.289-1.901) |
| Australasia | 6886.753(5877.291-7889.255) | 49.180(41.971-56.339) | 22498.785(17984.292-27196.813) | 96.068(76.791-116.128) | 2.267(1.822-2.728) | 2.497(2.231-2.765) |
| Caribbean | 34891.084(28951.788-42944.779) | 172.783(143.372-212.666) | 95481.777(77194.688-117496.178) | 296.563(239.764-364.939) | 1.737(1.366-2.159) | 2.153(2.035-2.272) |
| Central Asia | 12750.056(9452.286-16244.446) | 33.791(25.051-43.052) | 28232.343(22185.944-35188.723) | 46.143(36.261-57.513) | 1.214(1.026-1.455) | 0.783(0.642-0.924) |
| Central Europe | 62453.966(52886.106-72726.016) | 72.770(61.622-84.739) | 89440.308(73705.747-103511.611) | 97.529(80.371-112.873) | 0.432(0.295-0.584) | 1.648(1.394-1.902) |
| Central Latin America | 118675.484(99337.158-138896.393) | 145.121(121.474-169.848) | 563275.375(449185.892-686632.486) | 335.849(267.824-409.400) | 3.746(3.216-4.277) | 3.186(2.739-3.635) |
| Central Sub-Saharan Africa | 64391.276(49082.872-82944.427) | 268.582(204.729-345.969) | 163899.498(116524.252-220947.823) | 258.649(183.886-348.676) | 1.545(0.936-2.375) | -0.251(-0.374--0.127) |
| East Asia | 973539.754(799913.604-1175501.628) | 128.554(105.627-155.223) | 1710458.671(1375468.844-2079529.615) | 151.660(121.958-184.384) | 0.757(0.410-1.126) | 0.475(0.340-0.611) |
| Eastern Europe | 54880.354(44231.818-66120.567) | 34.469(27.781-41.529) | 78926.333(63617.885-94660.203) | 49.145(39.613-58.942) | 0.438(0.307-0.600) | 0.845(0.517-1.174) |
| Eastern Sub-Saharan Africa | 144584.291(111866.434-179196.750) | 180.894(139.960-224.199) | 273021.926(217947.003-341295.739) | 137.533(109.790-171.926) | 0.888(0.668-1.170) | -1.165(-1.363--0.966) |
| High-income Asia Pacific | 81743.515(70693.597-92941.016) | 66.429(57.450-75.529) | 183060.140(145513.402-222417.984) | 118.365(94.088-143.813) | 1.239(0.972-1.509) | 2.185(1.901-2.469) |
| High-income North America | 253831.177(216879.487-290775.380) | 127.119(108.614-145.621) | 1017048.932(899875.785-1116016.055) | 362.434(320.678-397.701) | 3.007(2.640-3.446) | 3.748(3.579-3.917) |
| North Africa and Middle East | 341340.075(262380.127-514181.212) | 210.159(161.545-316.576) | 1062018.487(853387.978-1260974.652) | 274.772(220.794-326.248) | 2.111(1.001-2.853) | 1.004(0.793-1.215) |
| Oceania | 2244.824(1641.079-2964.316) | 70.513(51.549-93.113) | 6646.704(5224.130-8389.195) | 88.139(69.275-111.245) | 1.961(1.274-2.893) | 0.636(0.578-0.694) |
| South Asia | 520147.159(419232.483-645461.620) | 94.403(76.088-117.147) | 1402006.399(1103137.634-1711379.630) | 120.543(94.847-147.142) | 1.695(1.238-2.130) | 0.710(0.633-0.788) |
| Southeast Asia | 832804.604(695675.928-986659.630) | 339.082(283.249-401.725) | 2228641.026(1821439.816-2641164.936) | 475.156(388.339-563.108) | 1.676(1.265-2.107) | 1.065(1.020-1.111) |
| Southern Latin America | 67560.808(56010.006-79219.013) | 224.015(185.715-262.670) | 116476.791(96182.601-136528.128) | 241.729(199.612-283.343) | 0.724(0.593-0.863) | 0.443(0.184-0.702) |
| Southern Sub-Saharan Africa | 47140.312(40165.625-58075.952) | 181.606(154.736-223.735) | 173149.763(145822.713-206625.543) | 353.083(297.359-421.346) | 2.673(2.060-3.220) | 1.771(1.371-2.172) |
| Tropical Latin America | 131998.827(110460.988-153267.013) | 158.481(132.622-184.016) | 353940.025(297022.325-413640.303) | 219.924(184.558-257.019) | 1.681(1.481-1.875) | 0.979(0.835-1.123) |
| Western Europe | 218498.843(179024.365-254443.614) | 76.380(62.581-88.945) | 459274.534(371015.484-541557.908) | 132.862(107.330-156.665) | 1.102(0.881-1.344) | 2.292(2.134-2.451) |
| Western Sub-Saharan Africa | 246620.560(199346.941-302703.574) | 287.891(232.707-353.359) | 598604.869(478029.656-735821.644) | 270.554(216.057-332.572) | 1.427(1.064-1.852) | -0.351(-0.449--0.252) |

CI, credibility interval. DALYs, disability adjusted life years. EAPC, estimated annual percentage change. SDI, Sociodemographic Index. UI, uncertainty interval.

Supplementary Table S4. Incidence of chronic kidney disease due to hypertension between 1990 and 2021 in 204 countries and territories.

|  | Rate per 100 000 (95% UI) | | | |  |
| --- | --- | --- | --- | --- | --- |
|  | 1990 |  | 2021 |  | 1990-2021 |
| Region | Incident cases | ASIR | Incident cases | ASIR | EAPC (95%CI) |
| Global | 462011.953(423695.599-504090.710) | 15.025(13.779-16.393) | 1278875.189(1192522.079-1362825.761) | 24.334(22.691-25.931) | 1.572(1.511-1.633) |
| Afghanistan | 1036.069(872.699-1217.914) | 23.706(19.968-27.867) | 2126.077(1792.421-2491.823) | 15.813(13.331-18.533) | -1.126(-1.424--0.826) |
| Albania | 148.048(124.981-176.435) | 7.963(6.722-9.489) | 566.841(490.348-665.511) | 27.641(23.911-32.452) | 4.508(4.253-4.764) |
| Algeria | 1926.801(1598.828-2289.815) | 16.368(13.582-19.452) | 9175.153(7849.596-10684.859) | 33.065(28.288-38.505) | 2.164(1.967-2.361) |
| American Samoa | 2.939(2.519-3.451) | 11.960(10.250-14.043) | 8.977(7.696-10.393) | 29.307(25.124-33.932) | 3.037(2.984-3.090) |
| Andorra | 9.013(7.794-10.548) | 22.034(19.055-25.788) | 24.962(21.977-28.618) | 35.051(30.860-40.185) | 1.536(1.330-1.742) |
| Angola | 192.548(161.952-228.606) | 4.269(3.591-5.069) | 863.691(734.407-1024.017) | 6.156(5.234-7.298) | 1.159(0.883-1.435) |
| Antigua and Barbuda | 6.694(5.788-7.824) | 18.421(15.929-21.531) | 22.438(19.429-26.058) | 33.980(29.424-39.462) | 2.225(2.049-2.400) |
| Argentina | 4519.724(3904.536-5209.875) | 22.504(19.441-25.940) | 10590.149(9206.162-12099.974) | 33.328(28.972-38.079) | 1.314(1.166-1.462) |
| Armenia | 136.757(112.321-164.402) | 6.535(5.367-7.856) | 455.997(393.624-532.562) | 20.429(17.634-23.859) | 3.959(3.852-4.067) |
| Australia | 3622.744(3204.314-4026.062) | 30.998(27.418-34.449) | 9937.837(8633.306-11189.148) | 50.823(44.152-57.223) | 1.562(1.472-1.652) |
| Austria | 1960.621(1681.173-2290.453) | 33.259(28.519-38.854) | 3759.862(3289.092-4284.681) | 52.022(45.509-59.284) | 1.684(1.604-1.763) |
| Azerbaijan | 282.722(232.410-342.776) | 6.737(5.538-8.168) | 1181.729(987.026-1406.381) | 15.855(13.243-18.869) | 2.924(2.625-3.224) |
| Bahamas | 18.068(15.368-21.035) | 12.123(10.311-14.114) | 71.919(62.150-82.623) | 26.373(22.791-30.298) | 2.763(2.686-2.840) |
| Bahrain | 32.244(27.610-38.240) | 10.525(9.012-12.482) | 285.839(242.346-342.237) | 25.384(21.521-30.392) | 2.287(1.902-2.674) |
| Bangladesh | 2590.493(2191.209-2996.893) | 5.313(4.494-6.146) | 11151.989(9533.460-12774.536) | 10.760(9.198-12.325) | 2.409(2.103-2.716) |
| Barbados | 31.890(27.536-37.344) | 18.924(16.341-22.161) | 92.816(80.632-106.535) | 39.896(34.659-45.793) | 2.523(2.476-2.569) |
| Belarus | 599.169(505.484-715.036) | 8.201(6.918-9.786) | 1236.171(1055.507-1442.063) | 16.935(14.460-19.756) | 2.376(2.122-2.630) |
| Belgium | 2716.146(2338.022-3162.855) | 36.181(31.144-42.131) | 4382.659(3861.182-5000.717) | 49.105(43.262-56.030) | 1.170(1.070-1.269) |
| Belize | 10.630(8.977-12.556) | 12.602(10.643-14.886) | 56.414(47.953-66.591) | 21.695(18.442-25.609) | 1.778(1.637-1.918) |
| Benin | 157.831(136.384-183.089) | 7.968(6.885-9.243) | 538.932(454.657-632.390) | 9.008(7.599-10.570) | 0.388(0.248-0.527) |
| Bermuda | 7.245(6.242-8.377) | 16.661(14.355-19.267) | 25.798(22.216-29.105) | 49.570(42.687-55.924) | 3.884(3.722-4.046) |
| Bhutan | 18.021(14.745-22.102) | 6.226(5.094-7.636) | 67.671(57.316-80.465) | 13.482(11.419-16.031) | 2.428(2.336-2.519) |
| Bolivia (Plurinational State of) | 339.973(291.946-390.695) | 11.212(9.628-12.885) | 1492.639(1312.116-1707.099) | 20.587(18.097-23.545) | 2.238(2.161-2.315) |
| Bosnia and Herzegovina | 292.220(247.317-344.927) | 9.687(8.199-11.435) | 894.753(774.320-1030.180) | 33.921(29.355-39.055) | 4.179(3.871-4.486) |
| Botswana | 51.617(43.816-61.313) | 8.919(7.571-10.594) | 196.924(167.330-231.834) | 13.339(11.335-15.704) | 1.123(0.826-1.422) |
| Brazil | 10355.015(9306.938-11394.460) | 12.734(11.445-14.013) | 40294.589(37001.496-43392.121) | 25.757(23.652-27.737) | 2.254(2.194-2.314) |
| Brunei Darussalam | 18.947(16.441-21.855) | 13.136(11.399-15.153) | 70.010(59.694-81.768) | 21.742(18.539-25.394) | 1.738(1.651-1.824) |
| Bulgaria | 948.833(812.269-1112.364) | 15.021(12.859-17.610) | 1974.883(1700.863-2285.895) | 35.955(30.966-41.617) | 2.822(2.733-2.911) |
| Burkina Faso | 312.516(267.586-371.112) | 8.073(6.913-9.587) | 899.713(772.203-1056.013) | 9.038(7.757-10.608) | 0.382(0.266-0.498) |
| Burundi | 126.733(108.881-148.074) | 5.284(4.540-6.174) | 313.205(269.301-362.699) | 5.268(4.529-6.100) | -0.278(-0.495--0.060) |
| Cabo Verde | 15.723(13.605-18.077) | 9.868(8.538-11.345) | 45.986(39.472-54.023) | 12.547(10.769-14.740) | 0.670(0.493-0.846) |
| Cambodia | 314.881(264.091-369.419) | 6.876(5.767-8.067) | 1309.376(1125.687-1545.506) | 12.574(10.810-14.842) | 1.953(1.737-2.170) |
| Cameroon | 445.731(374.554-528.777) | 9.906(8.324-11.751) | 1757.603(1472.718-2090.988) | 11.814(9.899-14.055) | 0.436(0.286-0.587) |
| Canada | 5970.440(5254.773-6671.545) | 30.480(26.826-34.059) | 13644.363(11830.156-15625.957) | 46.663(40.458-53.440) | 1.607(1.520-1.694) |
| Central African Republic | 56.993(47.152-68.226) | 4.620(3.823-5.531) | 145.301(120.985-175.618) | 5.610(4.671-6.780) | 0.565(0.309-0.822) |
| Chad | 210.859(179.380-244.378) | 8.430(7.172-9.770) | 532.997(443.324-630.550) | 7.838(6.520-9.273) | -0.243(-0.458--0.027) |
| Chile | 1377.786(1192.181-1588.442) | 17.199(14.882-19.828) | 5548.248(4883.746-6293.763) | 39.904(35.124-45.265) | 3.015(2.887-3.143) |
| China | 72363.018(64412.741-80284.416) | 9.894(8.807-10.977) | 222113.143(203154.884-239134.886) | 20.407(18.665-21.970) | 2.510(2.321-2.699) |
| Colombia | 2424.781(2050.298-2888.909) | 13.905(11.757-16.566) | 10733.746(9265.765-12391.112) | 31.111(26.857-35.915) | 2.936(2.856-3.016) |
| Comoros | 11.426(9.635-13.506) | 5.764(4.860-6.812) | 35.888(30.884-41.121) | 8.338(7.175-9.554) | 1.144(1.038-1.251) |
| Congo | 60.610(51.279-71.920) | 5.638(4.770-6.690) | 229.483(194.525-271.031) | 7.900(6.697-9.331) | 1.011(0.736-1.287) |
| Cook Islands | 1.314(1.129-1.524) | 12.741(10.950-14.779) | 4.423(3.829-5.000) | 35.205(30.477-39.795) | 3.450(3.382-3.518) |
| Costa Rica | 456.440(402.934-519.689) | 28.114(24.818-32.009) | 1475.714(1306.137-1673.832) | 43.674(38.655-49.537) | 1.529(1.449-1.610) |
| Croatia | 559.825(474.287-652.848) | 15.837(13.417-18.468) | 1495.078(1307.791-1699.866) | 43.935(38.432-49.953) | 3.532(3.273-3.791) |
| Cuba | 868.345(746.052-1010.681) | 12.069(10.369-14.047) | 3110.371(2678.544-3585.783) | 35.073(30.203-40.434) | 3.814(3.696-3.932) |
| Cyprus | 161.078(135.730-189.201) | 31.037(26.153-36.456) | 466.694(411.425-530.981) | 43.507(38.355-49.500) | 0.971(0.907-1.035) |
| Czechia | 1069.855(902.901-1233.504) | 14.756(12.453-17.013) | 3077.578(2636.351-3585.899) | 36.586(31.341-42.629) | 3.072(2.999-3.145) |
| Côte d'Ivoire | 344.463(287.061-407.996) | 6.524(5.437-7.728) | 1267.575(1067.793-1502.386) | 9.405(7.923-11.148) | 1.009(0.828-1.190) |
| Democratic People's Republic of Korea | 1228.335(1032.771-1450.857) | 9.595(8.067-11.333) | 3614.744(3139.884-4241.768) | 18.266(15.866-21.434) | 2.145(2.082-2.207) |
| Democratic Republic of the Congo | 814.234(689.768-961.472) | 4.936(4.181-5.828) | 2637.264(2211.097-3115.186) | 6.263(5.251-7.398) | 0.611(0.407-0.816) |
| Denmark | 1245.019(1054.507-1460.725) | 31.977(27.084-37.517) | 2214.138(1917.294-2523.068) | 48.581(42.068-55.360) | 1.500(1.403-1.596) |
| Djibouti | 7.524(6.308-8.966) | 3.961(3.321-4.720) | 47.550(40.265-55.486) | 6.511(5.513-7.598) | 1.541(1.269-1.814) |
| Dominica | 7.693(6.702-8.893) | 19.403(16.902-22.428) | 15.758(13.594-18.025) | 33.069(28.528-37.827) | 1.775(1.736-1.814) |
| Dominican Republic | 308.799(258.845-365.081) | 8.463(7.094-10.006) | 1450.091(1252.396-1668.827) | 20.360(17.584-23.431) | 2.991(2.883-3.100) |
| Ecuador | 578.198(494.420-677.219) | 11.516(9.848-13.489) | 3564.080(3085.266-4051.075) | 31.130(26.948-35.383) | 3.679(3.523-3.836) |
| Egypt | 4228.117(3512.994-4998.829) | 15.425(12.816-18.237) | 16979.563(14363.214-19686.648) | 28.741(24.313-33.324) | 1.867(1.703-2.030) |
| El Salvador | 406.380(339.731-488.269) | 15.935(13.321-19.146) | 1439.218(1251.050-1660.140) | 35.378(30.752-40.808) | 3.042(2.849-3.235) |
| Equatorial Guinea | 9.854(8.346-11.964) | 5.390(4.565-6.544) | 49.122(41.669-57.660) | 6.619(5.614-7.769) | 0.979(0.782-1.176) |
| Eritrea | 54.778(45.529-66.656) | 3.806(3.164-4.632) | 182.451(152.310-216.623) | 5.396(4.505-6.407) | 0.900(0.706-1.094) |
| Estonia | 113.439(95.152-133.177) | 10.227(8.579-12.007) | 319.919(278.539-366.509) | 31.044(27.029-35.565) | 3.879(3.785-3.973) |
| Eswatini | 32.048(27.007-38.625) | 9.733(8.202-11.731) | 80.063(67.042-95.348) | 12.909(10.809-15.373) | 0.776(0.504-1.048) |
| Ethiopia | 1027.144(909.927-1161.404) | 4.868(4.312-5.504) | 2970.146(2676.737-3306.620) | 5.741(5.173-6.391) | 0.496(0.314-0.678) |
| Fiji | 42.439(35.747-51.106) | 10.619(8.944-12.787) | 120.979(103.095-139.693) | 21.082(17.966-24.343) | 2.212(2.129-2.296) |
| Finland | 893.045(752.253-1038.901) | 23.870(20.107-27.769) | 1968.710(1716.256-2262.369) | 44.872(39.118-51.565) | 2.101(2.047-2.156) |
| France | 13103.351(11072.213-15163.022) | 31.476(26.597-36.424) | 24688.993(21694.280-28022.316) | 48.756(42.842-55.338) | 1.584(1.526-1.641) |
| Gabon | 38.349(32.572-45.163) | 8.067(6.852-9.501) | 111.456(93.830-132.878) | 11.269(9.487-13.435) | 1.008(0.789-1.228) |
| Gambia | 27.434(23.131-32.182) | 6.535(5.510-7.666) | 102.209(86.355-118.416) | 9.065(7.659-10.502) | 1.193(1.127-1.258) |
| Georgia | 337.151(278.734-393.941) | 9.048(7.480-10.572) | 552.677(467.757-643.572) | 20.638(17.467-24.032) | 2.833(2.711-2.955) |
| Germany | 21901.777(18806.850-25251.857) | 34.981(30.038-40.332) | 39077.473(34216.245-44146.783) | 56.287(49.285-63.589) | 1.809(1.717-1.901) |
| Ghana | 419.107(349.973-504.510) | 6.244(5.214-7.517) | 1644.970(1385.037-1948.269) | 9.173(7.724-10.865) | 1.205(1.130-1.279) |
| Greece | 3097.557(2657.490-3544.780) | 40.872(35.065-46.773) | 4876.911(4277.031-5570.252) | 58.974(51.720-67.358) | 1.495(1.391-1.598) |
| Greenland | 3.687(3.112-4.291) | 9.788(8.262-11.392) | 10.039(8.782-11.628) | 24.647(21.560-28.547) | 3.178(3.092-3.264) |
| Grenada | 8.519(7.340-9.825) | 18.994(16.365-21.905) | 24.555(20.893-28.314) | 33.859(28.809-39.042) | 2.072(1.854-2.291) |
| Guam | 8.330(7.044-9.914) | 10.078(8.521-11.993) | 32.341(28.186-37.647) | 29.212(25.459-34.005) | 3.563(3.471-3.655) |
| Guatemala | 517.963(436.001-606.666) | 14.912(12.552-17.465) | 2595.082(2249.020-3046.137) | 28.354(24.573-33.282) | 2.291(2.206-2.376) |
| Guinea | 256.185(219.036-298.194) | 9.464(8.092-11.016) | 569.416(479.602-661.811) | 9.545(8.040-11.094) | -0.122(-0.208--0.036) |
| Guinea-Bissau | 32.186(26.980-37.821) | 7.681(6.439-9.026) | 71.846(59.474-85.866) | 7.600(6.291-9.083) | -0.114(-0.288-0.061) |
| Guyana | 40.397(34.006-48.276) | 10.148(8.542-12.127) | 113.479(95.826-132.079) | 23.445(19.798-27.288) | 2.986(2.881-3.092) |
| Haiti | 320.272(269.907-382.480) | 10.555(8.895-12.605) | 982.939(837.487-1154.331) | 13.546(11.542-15.908) | 0.743(0.644-0.843) |
| Honduras | 290.565(248.039-347.363) | 14.554(12.424-17.398) | 1241.284(1076.369-1465.834) | 21.551(18.688-25.450) | 1.436(1.363-1.509) |
| Hungary | 982.276(828.607-1145.654) | 13.110(11.059-15.290) | 2847.967(2472.919-3317.614) | 36.882(32.025-42.964) | 3.554(3.447-3.662) |
| Iceland | 40.230(34.696-45.916) | 23.751(20.484-27.108) | 87.562(76.831-99.659) | 33.513(29.405-38.142) | 1.102(1.007-1.198) |
| India | 42898.682(38530.733-47643.487) | 9.691(8.705-10.763) | 132589.464(120811.241-145398.608) | 14.508(13.219-15.909) | 1.072(0.979-1.165) |
| Indonesia | 7131.159(6348.827-7923.456) | 7.313(6.510-8.125) | 25509.434(22849.653-28275.066) | 13.519(12.109-14.984) | 1.807(1.605-2.010) |
| Iran (Islamic Republic of) | 4830.252(4270.735-5430.851) | 18.875(16.689-21.222) | 19528.318(18053.449-21048.450) | 32.820(30.341-35.375) | 1.588(1.393-1.783) |
| Iraq | 1387.444(1191.492-1610.062) | 17.089(14.676-19.831) | 6548.990(5667.136-7574.806) | 27.765(24.026-32.114) | 1.685(1.379-1.991) |
| Ireland | 772.392(685.667-867.157) | 33.977(30.162-38.145) | 1364.643(1236.201-1512.815) | 37.700(34.151-41.793) | 0.373(0.089-0.657) |
| Israel | 954.163(822.192-1106.834) | 32.293(27.826-37.459) | 2681.187(2342.401-3084.573) | 43.102(37.656-49.587) | 0.972(0.924-1.020) |
| Italy | 14069.843(12600.904-15680.190) | 32.585(29.183-36.314) | 25855.473(23507.769-28168.630) | 52.411(47.652-57.100) | 1.654(1.610-1.697) |
| Jamaica | 210.598(183.850-244.644) | 16.543(14.442-19.218) | 540.631(474.565-619.209) | 27.242(23.913-31.202) | 1.766(1.663-1.870) |
| Japan | 31887.213(29301.673-34656.527) | 34.486(31.690-37.481) | 77678.264(70679.470-84103.775) | 72.962(66.388-78.997) | 2.562(2.497-2.627) |
| Jordan | 231.100(193.943-274.250) | 14.099(11.832-16.732) | 2146.334(1854.410-2477.369) | 29.007(25.061-33.480) | 2.367(2.288-2.445) |
| Kazakhstan | 643.746(530.564-782.604) | 6.603(5.442-8.027) | 1860.470(1552.792-2218.317) | 15.211(12.695-18.136) | 2.694(2.437-2.951) |
| Kenya | 452.640(410.020-497.190) | 4.838(4.383-5.315) | 1605.694(1455.678-1781.534) | 6.306(5.717-6.997) | 0.593(0.309-0.879) |
| Kiribati | 3.247(2.671-4.024) | 8.600(7.074-10.659) | 8.986(7.449-11.019) | 13.271(11.001-16.272) | 1.296(1.216-1.376) |
| Kuwait | 116.222(97.041-137.887) | 11.255(9.398-13.353) | 748.607(631.867-881.811) | 21.060(17.776-24.807) | 1.602(1.420-1.785) |
| Kyrgyzstan | 146.130(119.426-180.429) | 6.212(5.077-7.670) | 396.919(326.913-480.452) | 9.824(8.091-11.892) | 1.411(1.140-1.682) |
| Lao People's Democratic Republic | 199.859(164.657-238.559) | 10.513(8.662-12.549) | 627.038(524.841-752.148) | 14.296(11.966-17.149) | 0.910(0.720-1.101) |
| Latvia | 165.361(139.984-193.878) | 8.677(7.346-10.174) | 348.557(297.615-409.413) | 23.500(20.065-27.603) | 3.259(3.093-3.426) |
| Lebanon | 348.045(297.960-409.316) | 21.057(18.027-24.764) | 1596.276(1408.076-1831.344) | 41.203(36.345-47.270) | 2.071(1.811-2.332) |
| Lesotho | 75.218(63.383-87.964) | 10.819(9.117-12.652) | 129.923(108.457-151.755) | 12.546(10.473-14.654) | 0.287(0.033-0.543) |
| Liberia | 88.546(76.085-102.336) | 8.091(6.952-9.350) | 219.298(184.624-258.907) | 8.215(6.916-9.699) | -0.653(-0.919--0.386) |
| Libya | 302.074(256.456-350.626) | 15.843(13.450-18.389) | 1341.838(1156.620-1567.521) | 28.054(24.182-32.772) | 1.462(1.169-1.755) |
| Lithuania | 238.466(202.090-280.195) | 9.291(7.874-10.917) | 449.703(385.350-513.007) | 20.511(17.576-23.398) | 2.746(2.572-2.920) |
| Luxembourg | 96.582(84.362-112.459) | 32.970(28.798-38.389) | 201.093(176.960-225.825) | 39.481(34.742-44.336) | 0.757(0.586-0.929) |
| Madagascar | 249.697(212.850-291.895) | 4.825(4.113-5.641) | 684.775(574.474-808.219) | 5.030(4.220-5.937) | 0.116(-0.179-0.412) |
| Malawi | 212.629(182.352-247.681) | 5.024(4.308-5.852) | 531.706(459.145-607.661) | 6.003(5.184-6.861) | 0.526(0.341-0.711) |
| Malaysia | 965.475(824.954-1163.450) | 10.355(8.848-12.478) | 4503.898(3861.334-5345.910) | 20.893(17.912-24.799) | 2.431(2.346-2.516) |
| Maldives | 10.099(8.410-12.007) | 10.754(8.955-12.786) | 60.909(51.844-70.423) | 15.743(13.400-18.202) | 1.002(0.703-1.303) |
| Mali | 276.576(234.131-323.659) | 7.441(6.299-8.708) | 826.743(701.309-962.367) | 8.375(7.105-9.749) | 0.507(0.399-0.615) |
| Malta | 76.149(65.853-87.275) | 29.823(25.790-34.180) | 196.090(171.951-223.940) | 54.734(47.996-62.507) | 1.962(1.920-2.004) |
| Marshall Islands | 1.633(1.356-1.958) | 8.759(7.271-10.500) | 5.150(4.256-6.099) | 15.511(12.820-18.369) | 1.893(1.656-2.131) |
| Mauritania | 85.205(72.562-99.123) | 9.254(7.881-10.766) | 256.014(215.514-296.128) | 12.403(10.441-14.347) | 1.030(0.861-1.199) |
| Mauritius | 110.297(92.827-130.933) | 16.583(13.956-19.685) | 440.207(378.130-509.199) | 45.147(38.780-52.222) | 3.498(3.379-3.617) |
| Mexico | 7722.657(6947.682-8569.033) | 18.377(16.533-20.391) | 34819.280(32733.462-37087.248) | 40.429(38.007-43.062) | 2.806(2.734-2.877) |
| Micronesia (Federated States of) | 5.291(4.405-6.372) | 11.507(9.579-13.857) | 12.780(10.688-15.369) | 20.905(17.483-25.140) | 2.007(1.923-2.092) |
| Monaco | 11.641(10.039-13.477) | 45.529(39.265-52.710) | 17.076(14.655-19.599) | 54.918(47.131-63.032) | 0.567(0.517-0.618) |
| Mongolia | 79.943(65.312-96.460) | 7.817(6.386-9.432) | 251.851(210.377-300.854) | 12.446(10.396-14.867) | 1.454(1.166-1.743) |
| Montenegro | 67.822(58.920-79.711) | 16.475(14.312-19.363) | 169.875(145.090-199.899) | 36.324(31.024-42.743) | 2.750(2.625-2.875) |
| Morocco | 1785.985(1527.842-2073.576) | 13.869(11.865-16.103) | 8147.528(6991.921-9308.506) | 33.583(28.820-38.368) | 2.892(2.633-3.151) |
| Mozambique | 313.969(263.590-367.023) | 5.423(4.553-6.340) | 741.696(630.076-858.665) | 5.569(4.731-6.447) | 0.016(-0.205-0.237) |
| Myanmar | 2030.102(1686.611-2407.312) | 9.519(7.908-11.288) | 6233.897(5343.307-7227.502) | 17.486(14.988-20.273) | 2.015(1.776-2.255) |
| Namibia | 60.285(51.172-70.018) | 9.385(7.966-10.900) | 168.450(143.317-195.376) | 12.388(10.540-14.368) | 0.794(0.516-1.072) |
| Nauru | 0.490(0.398-0.586) | 9.813(7.960-11.726) | 0.878(0.708-1.050) | 14.898(12.011-17.807) | 1.266(1.128-1.405) |
| Nepal | 686.054(580.377-816.076) | 7.522(6.364-8.948) | 2939.425(2528.151-3461.331) | 15.740(13.537-18.534) | 2.308(2.217-2.399) |
| Netherlands | 3210.244(2813.913-3649.207) | 28.950(25.376-32.909) | 6636.071(5780.538-7532.083) | 49.085(42.757-55.712) | 1.931(1.822-2.040) |
| New Zealand | 732.715(637.654-849.212) | 31.634(27.530-36.664) | 1814.190(1613.517-2035.183) | 46.926(41.736-52.643) | 1.323(1.275-1.371) |
| Nicaragua | 264.305(223.819-313.270) | 16.143(13.670-19.134) | 1308.218(1126.662-1520.006) | 32.145(27.684-37.349) | 2.264(2.230-2.299) |
| Niger | 194.865(162.035-229.779) | 6.127(5.094-7.224) | 739.441(612.054-860.531) | 7.824(6.476-9.105) | 0.945(0.799-1.092) |
| Nigeria | 3438.188(3108.271-3796.793) | 8.280(7.486-9.144) | 9650.290(8740.325-10544.246) | 9.334(8.453-10.198) | 0.356(0.105-0.609) |
| Niue | 0.231(0.197-0.268) | 17.996(15.314-20.823) | 0.351(0.297-0.408) | 30.694(26.012-35.633) | 1.810(1.638-1.983) |
| North Macedonia | 153.098(129.684-176.662) | 11.819(10.011-13.638) | 561.782(481.894-655.541) | 32.475(27.857-37.895) | 3.393(3.150-3.636) |
| Northern Mariana Islands | 2.866(2.342-3.645) | 9.916(8.104-12.611) | 11.083(9.414-13.168) | 33.089(28.105-39.314) | 4.521(4.157-4.886) |
| Norway | 987.083(886.790-1121.271) | 31.500(28.300-35.782) | 1669.380(1507.967-1834.411) | 39.970(36.105-43.921) | 0.722(0.684-0.760) |
| Oman | 91.165(77.808-107.924) | 9.283(7.922-10.989) | 517.091(434.369-613.717) | 16.099(13.524-19.107) | 1.349(1.157-1.540) |
| Pakistan | 4723.225(4174.118-5327.711) | 9.416(8.321-10.621) | 13245.399(11675.144-14970.361) | 10.485(9.242-11.850) | 0.393(0.304-0.483) |
| Palau | 1.236(1.037-1.516) | 13.697(11.488-16.794) | 4.833(4.085-5.721) | 35.361(29.887-41.858) | 3.308(3.181-3.434) |
| Palestine | 149.824(126.408-176.201) | 17.557(14.813-20.648) | 682.294(581.180-795.384) | 25.013(21.306-29.159) | 0.940(0.733-1.148) |
| Panama | 220.282(188.723-255.407) | 16.920(14.496-19.618) | 977.995(854.031-1113.461) | 35.119(30.668-39.984) | 2.586(2.529-2.643) |
| Papua New Guinea | 100.151(82.751-121.882) | 5.072(4.191-6.173) | 389.317(324.961-462.709) | 6.999(5.842-8.318) | 0.983(0.762-1.204) |
| Paraguay | 258.123(220.479-299.745) | 13.079(11.172-15.188) | 1099.816(951.644-1258.463) | 24.450(21.156-27.977) | 2.038(1.968-2.109) |
| Peru | 1066.359(908.323-1249.322) | 9.708(8.269-11.374) | 5906.506(5155.548-6690.125) | 24.859(21.698-28.157) | 3.554(3.400-3.709) |
| Philippines | 3225.056(2943.590-3526.362) | 10.418(9.509-11.392) | 13199.089(12140.327-14284.931) | 19.277(17.730-20.863) | 2.023(1.971-2.075) |
| Poland | 3798.380(3316.725-4282.641) | 14.766(12.894-16.649) | 9338.751(8408.534-10658.636) | 30.579(27.533-34.901) | 1.598(1.368-1.829) |
| Portugal | 2075.774(1785.574-2479.453) | 29.007(24.952-34.648) | 4502.798(3896.856-5202.719) | 51.809(44.837-59.863) | 2.113(2.015-2.211) |
| Puerto Rico | 493.822(425.069-565.756) | 21.657(18.641-24.811) | 1391.125(1219.522-1583.804) | 52.677(46.179-59.973) | 3.069(2.929-3.210) |
| Qatar | 22.880(18.500-27.382) | 7.842(6.341-9.385) | 335.591(275.925-397.311) | 14.101(11.594-16.694) | 0.909(0.443-1.378) |
| Republic of Korea | 3706.409(3155.206-4339.213) | 13.088(11.142-15.323) | 16085.618(14163.884-18241.803) | 37.246(32.796-42.239) | 3.651(3.534-3.768) |
| Republic of Moldova | 160.369(136.392-188.829) | 5.595(4.759-6.588) | 397.760(337.539-465.030) | 13.729(11.650-16.051) | 2.756(2.484-3.029) |
| Romania | 1832.302(1557.076-2158.277) | 11.541(9.807-13.594) | 4908.702(4274.518-5529.901) | 32.949(28.693-37.119) | 3.558(3.400-3.717) |
| Russian Federation | 10291.505(9180.753-11537.747) | 9.727(8.677-10.905) | 24881.916(22548.849-27258.846) | 22.403(20.303-24.543) | 2.686(2.547-2.825) |
| Rwanda | 153.118(127.989-179.690) | 5.019(4.195-5.890) | 440.617(376.864-514.599) | 6.449(5.516-7.532) | 0.732(0.360-1.106) |
| Saint Kitts and Nevis | 4.824(4.095-5.581) | 20.882(17.724-24.158) | 15.011(12.764-17.633) | 33.669(28.630-39.551) | 1.633(1.484-1.781) |
| Saint Lucia | 10.412(8.988-12.149) | 14.918(12.877-17.407) | 45.070(39.028-52.415) | 33.249(28.791-38.667) | 2.681(2.652-2.711) |
| Saint Vincent and the Grenadines | 7.836(6.733-8.984) | 14.055(12.078-16.115) | 24.901(21.277-28.816) | 30.983(26.473-35.853) | 2.722(2.648-2.797) |
| Samoa | 9.522(8.109-11.347) | 12.313(10.486-14.672) | 23.273(19.884-27.166) | 20.651(17.644-24.106) | 1.677(1.638-1.716) |
| San Marino | 5.184(4.440-6.007) | 29.169(24.984-33.800) | 12.150(10.538-13.830) | 45.848(39.764-52.186) | 1.523(1.456-1.590) |
| Sao Tome and Principe | 5.925(4.969-6.999) | 11.528(9.667-13.618) | 15.277(12.893-18.271) | 13.305(11.229-15.913) | 0.354(0.218-0.490) |
| Saudi Arabia | 1093.496(931.796-1279.090) | 14.303(12.188-16.731) | 6680.372(5627.440-7910.893) | 24.230(20.411-28.693) | 1.245(1.069-1.421) |
| Senegal | 254.933(217.352-295.459) | 8.003(6.823-9.275) | 788.540(673.027-918.422) | 10.182(8.691-11.859) | 0.790(0.733-0.847) |
| Serbia | 950.720(811.562-1121.828) | 14.130(12.062-16.673) | 2652.796(2295.649-3039.081) | 37.652(32.583-43.134) | 3.549(3.348-3.751) |
| Seychelles | 7.357(6.399-8.569) | 17.673(15.371-20.585) | 22.647(19.400-26.386) | 30.234(25.899-35.227) | 1.614(1.517-1.712) |
| Sierra Leone | 152.453(129.492-176.633) | 7.834(6.654-9.077) | 363.396(310.382-423.251) | 8.419(7.190-9.805) | -0.086(-0.320-0.149) |
| Singapore | 349.004(307.149-390.592) | 16.414(14.446-18.370) | 1719.749(1510.920-1948.605) | 36.716(32.258-41.602) | 2.637(2.447-2.826) |
| Slovakia | 501.130(426.012-581.811) | 14.204(12.075-16.491) | 1361.675(1191.901-1565.838) | 31.605(27.665-36.344) | 2.550(2.463-2.637) |
| Slovenia | 190.350(163.166-222.109) | 13.436(11.517-15.677) | 652.043(559.688-754.653) | 39.166(33.618-45.329) | 3.660(3.515-3.805) |
| Solomon Islands | 12.838(10.098-16.348) | 8.888(6.991-11.318) | 35.991(28.971-44.397) | 10.201(8.211-12.583) | 0.220(0.046-0.396) |
| Somalia | 126.924(107.099-152.302) | 3.958(3.340-4.750) | 384.677(323.603-458.138) | 4.360(3.668-5.193) | 0.115(-0.071-0.301) |
| South Africa | 2150.305(1950.299-2365.419) | 11.089(10.058-12.198) | 6927.259(6325.897-7563.732) | 18.755(17.126-20.478) | 1.591(1.395-1.788) |
| South Sudan | 153.970(135.506-176.438) | 5.942(5.229-6.809) | 267.171(230.418-309.683) | 6.384(5.506-7.400) | -0.025(-0.453-0.404) |
| Spain | 9337.841(8127.512-10720.759) | 33.790(29.410-38.794) | 17958.779(15685.960-20216.259) | 48.878(42.692-55.022) | 1.220(1.180-1.259) |
| Sri Lanka | 1216.851(1037.592-1423.768) | 12.303(10.491-14.395) | 4701.499(4031.157-5494.235) | 30.589(26.227-35.746) | 3.094(3.027-3.160) |
| Sudan | 1219.471(1026.309-1430.751) | 13.587(11.435-15.941) | 4081.312(3475.406-4720.976) | 18.514(15.766-21.416) | 0.861(0.619-1.103) |
| Suriname | 28.509(23.971-33.653) | 13.216(11.112-15.600) | 113.420(97.137-130.572) | 29.127(24.946-33.532) | 2.706(2.594-2.818) |
| Sweden | 2177.217(1879.664-2512.319) | 33.595(29.004-38.766) | 2966.124(2503.486-3401.227) | 37.271(31.458-42.739) | 0.503(0.384-0.622) |
| Switzerland | 1819.060(1573.938-2107.679) | 34.392(29.757-39.848) | 3555.672(3102.753-4055.875) | 49.659(43.333-56.645) | 1.240(1.185-1.296) |
| Syrian Arab Republic | 831.357(711.405-981.460) | 15.494(13.259-18.292) | 3379.564(2914.993-3892.627) | 39.341(33.933-45.314) | 2.951(2.348-3.559) |
| Taiwan (Province of China) | 2633.123(2302.017-2970.524) | 20.128(17.597-22.707) | 8320.745(7407.530-9349.011) | 42.472(37.810-47.720) | 2.322(2.277-2.367) |
| Tajikistan | 113.382(92.548-139.254) | 4.545(3.710-5.582) | 428.067(356.273-519.852) | 7.504(6.245-9.113) | 1.741(1.387-2.096) |
| Thailand | 4360.516(3693.925-5186.489) | 12.818(10.859-15.246) | 20172.176(17666.466-22964.429) | 38.036(33.311-43.300) | 3.597(3.554-3.640) |
| Timor-Leste | 24.759(20.523-29.626) | 6.612(5.480-7.911) | 103.366(88.164-118.778) | 14.512(12.377-16.675) | 2.942(2.748-3.136) |
| Togo | 96.841(81.102-114.202) | 6.482(5.429-7.644) | 388.108(323.752-458.617) | 9.251(7.717-10.931) | 1.084(0.843-1.325) |
| Tokelau | 0.119(0.102-0.138) | 14.275(12.220-16.558) | 0.218(0.188-0.247) | 25.422(21.833-28.763) | 1.917(1.777-2.056) |
| Tonga | 5.996(5.075-7.152) | 13.180(11.157-15.722) | 12.651(10.842-14.611) | 22.306(19.116-25.762) | 1.616(1.556-1.676) |
| Trinidad and Tobago | 91.220(77.935-106.782) | 13.310(11.372-15.581) | 355.083(303.749-411.173) | 34.502(29.514-39.953) | 3.263(3.193-3.334) |
| Tunisia | 770.807(661.517-896.000) | 17.681(15.174-20.553) | 3407.070(2961.440-3898.287) | 41.263(35.866-47.212) | 2.584(2.419-2.749) |
| Turkmenistan | 115.233(93.849-144.178) | 6.352(5.174-7.948) | 388.626(319.648-473.595) | 12.153(9.996-14.810) | 2.292(2.013-2.572) |
| Tuvalu | 0.555(0.458-0.670) | 10.599(8.747-12.793) | 1.474(1.241-1.747) | 19.773(16.642-23.430) | 1.875(1.767-1.983) |
| Türkiye | 4642.032(4035.535-5305.168) | 15.076(13.106-17.229) | 24270.759(20770.271-27906.891) | 41.207(35.264-47.380) | 3.316(3.249-3.383) |
| Uganda | 331.696(285.184-375.384) | 4.743(4.078-5.368) | 913.721(784.228-1043.725) | 4.974(4.269-5.681) | 0.090(-0.197-0.379) |
| Ukraine | 3084.920(2602.161-3665.708) | 8.191(6.909-9.733) | 5168.950(4487.455-6034.276) | 14.925(12.957-17.424) | 1.900(1.754-2.047) |
| United Arab Emirates | 103.960(85.465-128.442) | 8.932(7.343-11.036) | 1762.615(1429.315-2195.105) | 22.210(18.010-27.660) | 1.693(0.718-2.677) |
| United Kingdom | 14161.061(12793.069-15892.039) | 33.347(30.126-37.424) | 21989.061(19908.677-24081.747) | 42.130(38.144-46.139) | 0.532(0.460-0.603) |
| United Republic of Tanzania | 661.195(567.098-767.319) | 6.058(5.196-7.030) | 1920.773(1636.343-2206.613) | 6.962(5.931-7.998) | 0.416(0.261-0.571) |
| United States of America | 65587.022(59560.738-72455.393) | 36.427(33.080-40.242) | 129152.306(119721.195-139043.504) | 51.387(47.635-55.323) | 0.951(0.812-1.089) |
| United States Virgin Islands | 10.151(8.605-11.782) | 15.776(13.372-18.310) | 33.098(28.590-38.272) | 48.902(42.242-56.547) | 3.845(3.724-3.965) |
| Uruguay | 537.263(469.289-613.745) | 26.050(22.755-29.759) | 972.832(858.677-1102.736) | 38.878(34.316-44.069) | 1.404(1.346-1.462) |
| Uzbekistan | 857.042(705.024-1056.706) | 8.335(6.856-10.276) | 3037.617(2519.634-3674.326) | 14.048(11.653-16.993) | 1.586(1.303-1.871) |
| Vanuatu | 5.341(4.498-6.412) | 7.766(6.540-9.322) | 22.278(18.654-26.689) | 13.458(11.269-16.122) | 1.692(1.584-1.801) |
| Venezuela (Bolivarian Republic of) | 1451.445(1236.670-1714.087) | 14.915(12.708-17.614) | 6835.851(5982.600-7740.775) | 38.238(33.465-43.300) | 3.016(2.929-3.103) |
| Viet Nam | 3185.258(2730.636-3771.886) | 9.217(7.901-10.914) | 12600.525(10732.843-15055.178) | 18.355(15.634-21.931) | 2.317(2.165-2.469) |
| Yemen | 607.193(497.384-715.646) | 11.618(9.517-13.693) | 2626.605(2200.133-3044.486) | 16.242(13.605-18.826) | 0.984(0.697-1.272) |
| Zambia | 178.166(153.050-209.346) | 5.506(4.730-6.469) | 552.785(469.584-645.942) | 6.101(5.182-7.129) | 0.276(0.075-0.477) |
| Zimbabwe | 397.288(337.804-469.299) | 9.196(7.819-10.862) | 790.446(666.985-938.247) | 10.385(8.763-12.327) | 0.007(-0.281-0.297) |

ASIR, age-standardized incidence rate; CI, credibility interval. EAPC, estimated annual percentage change. UI, uncertainty interval.

Supplementary Table S5. Mortality of chronic kidney disease due to hypertension between 1990 and 2021 in 204 countries and territories.

|  | Rate per 100 000 (95% UI) | | | |  |
| --- | --- | --- | --- | --- | --- |
|  | 1990 |  | 2021 |  | 1990-2021 |
| Region | Deaths cases | ASDR | Deaths cases | ASDR | EAPC (95%CI) |
| Global | 147950.889(122218.426-176320.795) | 4.811(3.975-5.734) | 453419.544(380122.953-523419.266) | 8.628(7.233-9.959) | 2.065(1.632-2.335) |
| Afghanistan | 894.859(604.812-1495.731) | 20.475(13.838-34.223) | 1367.730(795.033-2557.166) | 10.172(5.913-19.019) | -1.911(-2.112--1.710) |
| Albania | 24.559(18.208-32.639) | 1.321(0.979-1.755) | 56.217(38.276-80.498) | 2.741(1.866-3.925) | 2.550(2.149-2.952) |
| Algeria | 699.495(443.191-1304.750) | 5.942(3.765-11.084) | 3312.999(2432.559-4298.012) | 11.939(8.766-15.489) | 2.425(2.103-2.748) |
| American Samoa | 0.493(0.325-0.737) | 2.007(1.324-3.000) | 2.455(1.687-3.441) | 8.017(5.508-11.235) | 4.788(4.516-5.061) |
| Andorra | 0.932(0.605-1.419) | 2.279(1.478-3.469) | 3.256(2.124-4.749) | 4.572(2.983-6.669) | 2.683(2.339-3.029) |
| Angola | 372.515(264.370-511.305) | 8.259(5.862-11.337) | 1151.104(780.459-1581.186) | 8.204(5.562-11.269) | -0.106(-0.309-0.097) |
| Antigua and Barbuda | 5.749(4.717-6.921) | 15.822(12.981-19.047) | 14.481(11.771-17.466) | 21.931(17.826-26.452) | 1.661(1.202-2.121) |
| Argentina | 2664.415(2148.619-3189.478) | 13.266(10.698-15.880) | 4686.004(3762.433-5661.524) | 14.747(11.841-17.817) | 0.519(0.125-0.915) |
| Armenia | 1.100(0.801-1.400) | 0.053(0.038-0.067) | 22.720(15.955-30.723) | 1.018(0.715-1.376) | 9.580(8.328-10.847) |
| Australia | 303.607(247.595-360.268) | 2.598(2.119-3.083) | 1282.553(937.382-1683.422) | 6.559(4.794-8.609) | 3.613(3.224-4.003) |
| Austria | 148.158(111.975-190.871) | 2.513(1.899-3.238) | 818.318(642.234-978.708) | 11.322(8.886-13.542) | 7.858(6.918-8.806) |
| Azerbaijan | 13.248(9.234-18.780) | 0.316(0.220-0.448) | 42.982(28.250-60.704) | 0.577(0.379-0.814) | 2.154(1.879-2.429) |
| Bahamas | 10.509(8.636-12.617) | 7.051(5.794-8.465) | 42.644(33.075-53.954) | 15.638(12.129-19.785) | 3.208(2.958-3.458) |
| Bahrain | 14.620(10.054-20.418) | 4.772(3.282-6.665) | 83.176(60.887-106.388) | 7.386(5.407-9.448) | 0.134(-0.370-0.640) |
| Bangladesh | 1184.354(890.032-1647.544) | 2.429(1.825-3.379) | 3009.289(2192.977-4303.490) | 2.903(2.116-4.152) | 0.637(0.436-0.839) |
| Barbados | 19.320(15.523-22.992) | 11.465(9.212-13.644) | 46.950(34.352-59.988) | 20.181(14.766-25.785) | 2.063(1.788-2.338) |
| Belarus | 11.611(9.115-14.278) | 0.159(0.125-0.195) | 44.314(32.813-56.857) | 0.607(0.450-0.779) | 4.328(3.473-5.191) |
| Belgium | 324.982(245.369-420.460) | 4.329(3.268-5.601) | 728.819(517.331-959.578) | 8.166(5.796-10.752) | 2.700(2.497-2.904) |
| Belize | 6.955(5.758-8.197) | 8.246(6.826-9.718) | 38.148(30.666-46.106) | 14.671(11.793-17.731) | 2.331(1.948-2.714) |
| Benin | 287.055(232.030-349.577) | 14.491(11.713-17.647) | 733.019(574.181-940.436) | 12.252(9.597-15.719) | -0.527(-0.607--0.446) |
| Bermuda | 2.921(2.393-3.474) | 6.719(5.503-7.991) | 7.902(6.051-9.947) | 15.183(11.628-19.112) | 3.297(2.841-3.754) |
| Bhutan | 7.155(4.688-10.262) | 2.472(1.620-3.546) | 25.223(16.513-36.584) | 5.025(3.290-7.289) | 2.197(2.107-2.286) |
| Bolivia (Plurinational State of) | 352.635(266.428-480.355) | 11.630(8.787-15.842) | 1418.894(1026.954-1941.598) | 19.570(14.164-26.779) | 1.977(1.851-2.103) |
| Bosnia and Herzegovina | 34.075(25.193-45.578) | 1.130(0.835-1.511) | 98.195(71.049-128.928) | 3.723(2.694-4.888) | 4.452(3.609-5.301) |
| Botswana | 37.778(25.953-54.787) | 6.527(4.484-9.466) | 126.188(95.061-179.878) | 8.548(6.439-12.185) | 0.893(0.714-1.073) |
| Brazil | 4516.539(3743.478-5274.687) | 5.554(4.604-6.487) | 15323.395(12331.613-18184.759) | 9.795(7.883-11.624) | 1.889(1.738-2.040) |
| Brunei Darussalam | 5.656(4.117-8.070) | 3.921(2.855-5.595) | 15.140(11.272-19.218) | 4.702(3.501-5.968) | 1.019(0.753-1.286) |
| Bulgaria | 114.912(88.350-143.281) | 1.819(1.399-2.268) | 437.981(323.938-567.250) | 7.974(5.898-10.327) | 5.786(5.229-6.346) |
| Burkina Faso | 568.317(443.526-711.848) | 14.682(11.458-18.390) | 1463.522(1142.993-1844.252) | 14.701(11.481-18.525) | -0.025(-0.151-0.100) |
| Burundi | 150.583(103.351-210.477) | 6.279(4.309-8.776) | 255.622(172.726-402.735) | 4.299(2.905-6.773) | -1.791(-2.072--1.509) |
| Cabo Verde | 15.600(12.454-19.071) | 9.790(7.816-11.969) | 54.249(34.891-69.831) | 14.801(9.520-19.053) | 1.039(0.730-1.349) |
| Cambodia | 445.298(351.829-572.072) | 9.724(7.683-12.492) | 1303.476(948.127-1715.113) | 12.518(9.105-16.471) | 0.768(0.591-0.946) |
| Cameroon | 868.662(653.656-1144.092) | 19.304(14.526-25.425) | 2383.982(1694.855-3441.827) | 16.025(11.393-23.135) | -1.634(-2.179--1.086) |
| Canada | 1006.496(833.888-1161.344) | 5.138(4.257-5.929) | 3981.482(3140.557-4613.266) | 13.616(10.740-15.777) | 3.651(3.435-3.867) |
| Central African Republic | 133.340(101.707-173.220) | 10.810(8.245-14.043) | 253.611(178.215-366.317) | 9.791(6.881-14.143) | -0.313(-0.449--0.176) |
| Chad | 274.307(198.633-390.608) | 10.967(7.942-15.617) | 631.280(453.541-950.199) | 9.284(6.670-13.974) | -0.657(-0.807--0.508) |
| Chile | 419.984(340.496-502.319) | 5.243(4.250-6.270) | 1691.114(1311.773-2054.854) | 12.163(9.434-14.779) | 3.240(2.866-3.614) |
| China | 28667.414(22845.475-35841.736) | 3.919(3.123-4.900) | 65231.195(49295.002-82461.552) | 5.993(4.529-7.576) | 1.300(1.142-1.458) |
| Colombia | 728.343(596.622-865.712) | 4.177(3.421-4.964) | 2310.040(1686.015-2882.351) | 6.696(4.887-8.354) | 1.966(1.775-2.158) |
| Comoros | 12.107(8.525-16.472) | 6.107(4.300-8.308) | 36.412(25.094-50.331) | 8.460(5.830-11.693) | 1.020(0.885-1.156) |
| Congo | 147.925(104.035-192.248) | 13.759(9.677-17.882) | 358.455(233.012-484.983) | 12.340(8.022-16.696) | -0.556(-0.791--0.320) |
| Cook Islands | 0.222(0.155-0.313) | 2.155(1.499-3.035) | 0.653(0.464-0.882) | 5.196(3.690-7.020) | 3.025(2.820-3.231) |
| Costa Rica | 60.254(47.730-72.628) | 3.711(2.940-4.473) | 388.190(296.187-477.375) | 11.489(8.766-14.128) | 3.957(3.711-4.204) |
| Croatia | 85.033(65.911-107.293) | 2.405(1.865-3.035) | 262.048(190.574-341.618) | 7.701(5.600-10.039) | 5.581(5.062-6.102) |
| Cuba | 248.163(202.907-290.761) | 3.449(2.820-4.041) | 1006.190(803.068-1222.494) | 11.346(9.055-13.785) | 4.563(4.273-4.853) |
| Cyprus | 33.441(22.792-48.009) | 6.443(4.392-9.250) | 65.136(45.874-87.466) | 6.072(4.277-8.154) | -0.525(-0.726--0.323) |
| Czechia | 184.055(139.822-231.664) | 2.539(1.928-3.195) | 305.606(226.093-391.937) | 3.633(2.688-4.659) | 1.447(1.128-1.766) |
| Côte d'Ivoire | 469.061(356.155-590.230) | 8.884(6.746-11.179) | 1428.111(1079.919-1885.843) | 10.596(8.013-13.993) | 0.380(0.269-0.492) |
| Democratic People's Republic of Korea | 512.405(351.418-733.674) | 4.003(2.745-5.731) | 1169.701(835.060-1614.528) | 5.911(4.220-8.158) | 1.399(1.299-1.498) |
| Democratic Republic of the Congo | 1511.606(1110.332-2040.665) | 9.163(6.730-12.370) | 3844.451(2502.745-5298.989) | 9.129(5.943-12.583) | -0.175(-0.282--0.067) |
| Denmark | 67.928(51.667-86.563) | 1.745(1.327-2.223) | 307.490(244.957-372.520) | 6.747(5.375-8.174) | 4.532(4.357-4.708) |
| Djibouti | 5.473(3.716-8.003) | 2.881(1.956-4.213) | 36.073(24.420-50.830) | 4.939(3.344-6.960) | 1.778(1.666-1.890) |
| Dominica | 5.935(4.600-7.295) | 14.969(11.600-18.399) | 12.142(9.159-15.724) | 25.481(19.221-32.998) | 1.896(1.833-1.960) |
| Dominican Republic | 189.427(148.581-243.358) | 5.192(4.072-6.670) | 813.233(530.722-1094.749) | 11.418(7.452-15.371) | 3.208(2.912-3.506) |
| Ecuador | 369.872(298.727-439.848) | 7.367(5.950-8.761) | 2170.499(1541.446-3021.191) | 18.958(13.463-26.388) | 3.337(2.359-4.324) |
| Egypt | 3314.299(2406.079-5426.676) | 12.091(8.778-19.798) | 10861.409(8128.260-13570.747) | 18.385(13.759-22.971) | 1.583(1.428-1.738) |
| El Salvador | 163.270(126.098-240.064) | 6.402(4.944-9.413) | 1043.433(737.125-1346.086) | 25.649(18.119-33.088) | 4.706(4.162-5.253) |
| Equatorial Guinea | 21.923(15.520-28.759) | 11.992(8.489-15.731) | 70.183(41.144-106.740) | 9.456(5.544-14.382) | -0.424(-0.934-0.088) |
| Eritrea | 50.095(32.770-74.970) | 3.481(2.277-5.209) | 145.071(91.871-245.513) | 4.290(2.717-7.261) | 0.571(0.529-0.612) |
| Estonia | 18.090(14.285-22.291) | 1.631(1.288-2.010) | 128.660(102.749-155.138) | 12.485(9.970-15.054) | 6.459(5.356-7.574) |
| Eswatini | 32.626(24.099-42.733) | 9.909(7.319-12.978) | 88.308(57.158-126.325) | 14.238(9.216-20.367) | 1.443(0.839-2.051) |
| Ethiopia | 2751.582(2073.662-3549.879) | 13.040(9.827-16.823) | 4099.546(3129.701-5233.196) | 7.923(6.049-10.114) | -2.027(-2.346--1.706) |
| Fiji | 10.001(6.483-15.000) | 2.502(1.622-3.753) | 33.876(22.124-47.160) | 5.903(3.855-8.218) | 2.401(2.197-2.606) |
| Finland | 12.885(9.628-16.841) | 0.344(0.257-0.450) | 61.418(44.179-78.798) | 1.400(1.007-1.796) | 5.222(4.666-5.780) |
| France | 1737.356(1298.148-2234.096) | 4.173(3.118-5.367) | 4174.504(3310.051-4955.704) | 8.244(6.537-9.786) | 2.879(2.546-3.213) |
| Gabon | 83.456(62.515-105.227) | 17.557(13.151-22.137) | 188.734(98.067-260.870) | 19.083(9.915-26.376) | 0.280(0.208-0.353) |
| Gambia | 40.819(30.450-52.854) | 9.723(7.253-12.590) | 156.789(115.397-206.857) | 13.905(10.234-18.346) | 1.216(1.094-1.338) |
| Georgia | 7.860(5.494-11.374) | 0.211(0.147-0.305) | 36.772(25.970-49.312) | 1.373(0.970-1.841) | 6.514(5.795-7.237) |
| Germany | 1866.119(1387.986-2421.289) | 2.981(2.217-3.867) | 7847.517(5371.512-10536.026) | 11.304(7.737-15.176) | 6.383(5.690-7.079) |
| Ghana | 443.364(312.379-667.724) | 6.606(4.654-9.948) | 1941.548(1452.428-2505.224) | 10.827(8.100-13.971) | 1.767(1.572-1.962) |
| Greece | 479.521(354.871-619.314) | 6.327(4.682-8.172) | 1237.497(976.506-1485.550) | 14.964(11.808-17.964) | 3.123(1.766-4.498) |
| Greenland | 1.236(0.967-1.544) | 3.280(2.567-4.098) | 3.270(2.247-4.291) | 8.028(5.517-10.535) | 3.428(3.139-3.718) |
| Grenada | 8.546(6.947-10.239) | 19.052(15.489-22.827) | 18.826(14.629-23.251) | 25.958(20.172-32.060) | 1.251(0.893-1.610) |
| Guam | 4.632(3.712-6.085) | 5.604(4.490-7.361) | 14.960(12.304-17.487) | 13.513(11.114-15.795) | 3.755(3.471-4.041) |
| Guatemala | 209.654(167.692-255.225) | 6.036(4.828-7.348) | 1196.577(906.058-1506.087) | 13.074(9.900-16.456) | 3.538(3.032-4.045) |
| Guinea | 403.781(302.888-545.210) | 14.917(11.190-20.142) | 747.818(546.739-1065.487) | 12.536(9.165-17.861) | -0.381(-0.662--0.099) |
| Guinea-Bissau | 67.888(50.921-85.460) | 16.202(12.153-20.396) | 111.317(83.529-148.197) | 11.775(8.835-15.676) | -1.149(-1.226--1.072) |
| Guyana | 33.352(27.243-40.275) | 8.378(6.844-10.117) | 98.410(69.875-132.123) | 20.331(14.436-27.297) | 4.214(3.776-4.654) |
| Haiti | 241.193(158.465-449.357) | 7.949(5.222-14.809) | 579.848(307.846-1227.422) | 7.991(4.242-16.915) | 0.144(0.060-0.228) |
| Honduras | 50.655(38.011-70.987) | 2.537(1.904-3.556) | 322.557(225.723-428.137) | 5.600(3.919-7.433) | 2.810(2.576-3.044) |
| Hungary | 152.322(118.927-187.454) | 2.033(1.587-2.502) | 465.485(340.250-608.168) | 6.028(4.406-7.876) | 4.870(4.384-5.358) |
| Iceland | 3.168(2.468-3.954) | 1.870(1.457-2.335) | 11.239(8.421-13.779) | 4.302(3.223-5.274) | 3.274(3.079-3.469) |
| India | 10846.663(7976.465-13755.034) | 2.450(1.802-3.107) | 33933.531(25745.261-44054.633) | 3.713(2.817-4.820) | 1.198(0.999-1.398) |
| Indonesia | 7902.642(6397.461-9957.045) | 8.104(6.560-10.210) | 23586.812(18239.792-29577.293) | 12.500(9.666-15.675) | 1.455(1.391-1.519) |
| Iran (Islamic Republic of) | 1457.761(1049.492-2068.213) | 5.696(4.101-8.082) | 5995.932(4691.313-7074.559) | 10.077(7.884-11.890) | 2.080(1.922-2.237) |
| Iraq | 860.016(615.309-1178.314) | 10.593(7.579-14.513) | 2523.672(1631.215-3326.600) | 10.699(6.916-14.103) | -0.101(-0.446-0.244) |
| Ireland | 54.533(41.015-70.715) | 2.399(1.804-3.111) | 135.066(98.573-181.219) | 3.731(2.723-5.006) | 1.965(1.634-2.297) |
| Israel | 230.162(176.222-294.000) | 7.790(5.964-9.950) | 716.424(507.874-928.753) | 11.517(8.165-14.930) | 1.964(1.573-2.356) |
| Italy | 2011.876(1562.476-2507.307) | 4.659(3.619-5.807) | 5749.004(4101.086-7409.750) | 11.654(8.313-15.020) | 2.951(2.677-3.226) |
| Jamaica | 153.850(126.018-180.542) | 12.085(9.899-14.182) | 321.808(227.114-425.766) | 16.216(11.444-21.454) | 0.415(-0.203-1.037) |
| Japan | 2351.135(1769.793-3034.190) | 2.543(1.914-3.281) | 8055.205(5458.493-10962.875) | 7.566(5.127-10.297) | 3.819(3.489-4.150) |
| Jordan | 90.720(67.578-125.398) | 5.535(4.123-7.650) | 511.746(375.588-675.264) | 6.916(5.076-9.126) | 0.603(0.445-0.762) |
| Kazakhstan | 26.413(19.408-34.414) | 0.271(0.199-0.353) | 94.493(68.823-128.612) | 0.773(0.563-1.051) | 2.854(2.478-3.232) |
| Kenya | 343.815(234.867-555.001) | 3.675(2.511-5.932) | 1305.343(924.982-1775.411) | 5.127(3.633-6.973) | 1.290(1.211-1.368) |
| Kiribati | 1.029(0.746-1.423) | 2.726(1.976-3.768) | 2.756(1.607-4.345) | 4.071(2.373-6.416) | 0.891(0.620-1.164) |
| Kuwait | 46.360(37.159-54.889) | 4.490(3.599-5.316) | 124.097(87.007-161.171) | 3.491(2.448-4.534) | -1.355(-1.766--0.942) |
| Kyrgyzstan | 5.257(3.850-6.762) | 0.223(0.164-0.287) | 20.121(14.547-26.016) | 0.498(0.360-0.644) | 1.136(0.309-1.971) |
| Lao People's Democratic Republic | 373.595(266.027-508.843) | 19.652(13.994-26.767) | 779.014(525.107-1074.601) | 17.761(11.972-24.501) | -0.524(-0.601--0.448) |
| Latvia | 6.951(5.476-8.577) | 0.365(0.287-0.450) | 31.475(22.514-42.325) | 2.122(1.518-2.854) | 6.611(5.480-7.755) |
| Lebanon | 195.445(137.994-271.900) | 11.825(8.349-16.450) | 798.179(614.944-1022.119) | 20.602(15.873-26.383) | 2.200(1.933-2.468) |
| Lesotho | 58.470(43.261-79.570) | 8.410(6.222-11.445) | 160.466(109.533-221.087) | 15.496(10.577-21.349) | 2.652(2.196-3.110) |
| Liberia | 185.916(142.186-244.434) | 16.987(12.992-22.334) | 354.522(259.480-478.522) | 13.281(9.720-17.926) | -1.481(-1.833--1.128) |
| Libya | 166.908(114.518-245.234) | 8.754(6.006-12.862) | 713.914(468.269-967.394) | 14.926(9.790-20.226) | 2.250(2.055-2.446) |
| Lithuania | 11.774(9.414-14.579) | 0.459(0.367-0.568) | 48.260(35.524-62.796) | 2.201(1.620-2.864) | 4.097(3.630-4.567) |
| Luxembourg | 9.343(6.976-12.059) | 3.189(2.381-4.117) | 30.974(22.098-41.640) | 6.081(4.339-8.175) | 2.493(2.246-2.740) |
| Madagascar | 213.769(149.114-323.252) | 4.131(2.882-6.247) | 416.192(274.998-580.283) | 3.057(2.020-4.263) | -0.880(-1.120--0.640) |
| Malawi | 225.787(163.271-306.312) | 5.335(3.858-7.237) | 533.360(393.682-695.688) | 6.022(4.445-7.855) | 0.208(-0.080-0.497) |
| Malaysia | 893.950(745.291-1069.337) | 9.588(7.993-11.469) | 3341.937(2720.128-3995.710) | 15.503(12.618-18.536) | 1.349(1.180-1.517) |
| Maldives | 16.587(12.893-22.367) | 17.662(13.729-23.817) | 41.651(33.447-50.615) | 10.765(8.645-13.082) | -1.992(-2.145--1.839) |
| Mali | 490.643(379.211-647.377) | 13.200(10.202-17.417) | 1105.294(840.519-1500.992) | 11.197(8.515-15.206) | -0.244(-0.457--0.031) |
| Malta | 7.962(5.988-10.236) | 3.118(2.345-4.009) | 26.367(18.567-34.771) | 7.360(5.182-9.705) | 2.802(2.623-2.981) |
| Marshall Islands | 0.554(0.316-1.021) | 2.971(1.694-5.474) | 1.783(0.506-4.863) | 5.371(1.525-14.646) | 1.923(1.862-1.985) |
| Mauritania | 153.723(115.290-197.865) | 16.696(12.522-21.491) | 320.920(214.504-461.176) | 15.548(10.392-22.343) | -0.404(-0.600--0.208) |
| Mauritius | 130.027(110.757-146.907) | 19.549(16.652-22.087) | 615.207(520.020-701.021) | 63.094(53.332-71.895) | 4.100(3.691-4.511) |
| Mexico | 3044.875(2446.014-3627.108) | 7.246(5.821-8.631) | 14835.375(11607.329-18225.396) | 17.225(13.477-21.162) | 3.572(2.934-4.215) |
| Micronesia (Federated States of) | 1.669(1.002-2.743) | 3.629(2.179-5.966) | 3.903(2.537-5.905) | 6.384(4.151-9.659) | 1.810(1.564-2.057) |
| Monaco | 0.931(0.647-1.314) | 3.642(2.531-5.139) | 2.338(1.603-3.207) | 7.520(5.155-10.314) | 2.510(2.138-2.882) |
| Mongolia | 7.070(5.061-9.671) | 0.691(0.495-0.946) | 12.817(9.162-17.358) | 0.633(0.453-0.858) | -0.718(-0.958--0.477) |
| Montenegro | 24.677(18.124-31.956) | 5.994(4.402-7.762) | 58.370(39.803-81.292) | 12.481(8.511-17.382) | 2.770(2.340-3.201) |
| Morocco | 632.382(392.010-1265.116) | 4.911(3.044-9.824) | 2597.382(1812.826-3547.776) | 10.706(7.472-14.623) | 3.131(2.816-3.446) |
| Mozambique | 217.038(154.568-328.573) | 3.749(2.670-5.676) | 566.362(403.564-828.781) | 4.252(3.030-6.223) | 0.854(0.657-1.052) |
| Myanmar | 2624.035(1929.172-3584.644) | 12.304(9.046-16.808) | 5019.158(3771.471-6565.807) | 14.078(10.579-18.417) | 0.218(0.133-0.304) |
| Namibia | 41.891(30.016-62.799) | 6.521(4.673-9.776) | 114.409(80.977-155.444) | 8.414(5.955-11.432) | 0.636(0.352-0.922) |
| Nauru | 0.180(0.103-0.303) | 3.598(2.060-6.062) | 0.347(0.219-0.534) | 5.888(3.721-9.057) | 1.334(1.201-1.468) |
| Nepal | 218.795(151.886-303.542) | 2.399(1.665-3.328) | 785.604(553.049-1082.341) | 4.207(2.961-5.796) | 2.247(1.776-2.720) |
| Netherlands | 321.863(245.894-407.817) | 2.903(2.218-3.678) | 1271.199(997.433-1499.347) | 9.403(7.378-11.090) | 5.089(4.715-5.465) |
| New Zealand | 66.469(52.904-80.218) | 2.870(2.284-3.463) | 270.974(209.982-330.756) | 7.009(5.431-8.555) | 3.113(2.666-3.561) |
| Nicaragua | 93.246(72.100-128.458) | 5.695(4.404-7.846) | 576.502(433.737-744.288) | 14.166(10.658-18.288) | 3.467(3.033-3.902) |
| Niger | 277.244(207.935-390.178) | 8.717(6.538-12.267) | 771.587(544.901-1211.396) | 8.164(5.765-12.817) | -0.109(-0.238-0.020) |
| Nigeria | 4412.191(3395.962-5640.288) | 10.626(8.179-13.584) | 9254.881(6974.106-12093.240) | 8.951(6.745-11.696) | -0.625(-0.722--0.528) |
| Niue | 0.084(0.059-0.120) | 6.558(4.591-9.313) | 0.128(0.069-0.211) | 11.224(6.046-18.446) | 1.877(1.677-2.078) |
| North Macedonia | 34.407(24.762-45.271) | 2.656(1.912-3.495) | 80.755(55.809-109.985) | 4.668(3.226-6.358) | 2.135(1.880-2.390) |
| Northern Mariana Islands | 0.280(0.196-0.403) | 0.967(0.677-1.395) | 1.206(0.855-1.728) | 3.601(2.553-5.158) | 4.912(4.390-5.436) |
| Norway | 64.010(48.782-80.387) | 2.043(1.557-2.565) | 228.352(169.565-291.384) | 5.467(4.060-6.977) | 4.436(3.930-4.944) |
| Oman | 41.823(28.942-64.842) | 4.258(2.947-6.602) | 192.800(141.557-249.568) | 6.003(4.407-7.770) | 1.438(1.220-1.655) |
| Pakistan | 1947.458(1437.230-2656.569) | 3.882(2.865-5.296) | 5775.476(4278.580-7785.907) | 4.572(3.387-6.163) | 0.179(-0.036-0.396) |
| Palau | 0.287(0.195-0.396) | 3.176(2.156-4.389) | 0.967(0.626-1.391) | 7.071(4.582-10.180) | 2.875(2.763-2.988) |
| Palestine | 113.855(78.608-158.847) | 13.342(9.212-18.615) | 275.372(212.366-347.633) | 10.095(7.785-12.744) | -0.897(-1.034--0.760) |
| Panama | 45.283(36.535-54.450) | 3.478(2.806-4.182) | 345.718(246.392-435.124) | 12.415(8.848-15.625) | 4.599(4.119-5.082) |
| Papua New Guinea | 20.880(12.795-32.268) | 1.057(0.648-1.634) | 71.407(49.041-105.532) | 1.284(0.882-1.897) | 0.475(0.356-0.594) |
| Paraguay | 113.717(87.908-141.846) | 5.762(4.454-7.187) | 472.380(334.394-645.971) | 10.502(7.434-14.361) | 2.104(1.961-2.246) |
| Peru | 1000.053(791.874-1257.458) | 9.104(7.209-11.448) | 3541.503(2393.053-4619.681) | 14.905(10.072-19.443) | 1.692(1.501-1.883) |
| Philippines | 3716.119(3182.315-4461.798) | 12.005(10.280-14.413) | 14950.012(11976.748-17946.031) | 21.834(17.492-26.209) | 2.322(2.182-2.463) |
| Poland | 853.992(677.214-1045.926) | 3.320(2.633-4.066) | 918.391(678.546-1174.175) | 3.007(2.222-3.845) | 1.381(0.488-2.282) |
| Portugal | 267.647(201.556-338.161) | 3.740(2.817-4.725) | 907.844(637.117-1211.315) | 10.446(7.331-13.937) | 3.624(3.236-4.014) |
| Puerto Rico | 276.540(219.766-335.220) | 12.128(9.638-14.701) | 655.263(522.735-806.580) | 24.812(19.794-30.542) | 2.892(2.422-3.363) |
| Qatar | 8.269(4.480-13.588) | 2.834(1.535-4.657) | 60.816(44.355-82.085) | 2.555(1.864-3.449) | -1.523(-2.142--0.899) |
| Republic of Korea | 1108.685(964.539-1270.233) | 3.915(3.406-4.486) | 3136.453(2293.612-3966.625) | 7.262(5.311-9.185) | 2.336(1.781-2.893) |
| Republic of Moldova | 8.852(6.937-11.082) | 0.309(0.242-0.387) | 24.902(18.657-31.494) | 0.860(0.644-1.087) | 2.598(2.116-3.081) |
| Romania | 207.828(171.403-243.307) | 1.309(1.080-1.532) | 296.563(230.921-366.008) | 1.991(1.550-2.457) | 1.784(1.098-2.474) |
| Russian Federation | 874.857(699.704-1087.697) | 0.827(0.661-1.028) | 1813.495(1376.130-2290.112) | 1.633(1.239-2.062) | 1.961(1.043-2.887) |
| Rwanda | 218.260(164.354-285.904) | 7.154(5.387-9.372) | 403.349(267.827-567.130) | 5.904(3.920-8.301) | -1.600(-2.185--1.011) |
| Saint Kitts and Nevis | 4.925(3.895-5.929) | 21.319(16.858-25.666) | 10.453(7.881-13.143) | 23.446(17.676-29.480) | 0.943(0.555-1.332) |
| Saint Lucia | 9.563(7.837-11.173) | 13.701(11.229-16.009) | 30.160(23.320-37.592) | 22.250(17.203-27.732) | 2.111(1.680-2.543) |
| Saint Vincent and the Grenadines | 5.737(4.736-6.817) | 10.291(8.496-12.228) | 17.133(13.611-20.624) | 21.317(16.935-25.661) | 3.108(2.727-3.490) |
| Samoa | 2.854(1.828-4.168) | 3.691(2.364-5.389) | 6.848(4.804-9.489) | 6.076(4.263-8.420) | 1.573(1.486-1.661) |
| San Marino | 0.385(0.271-0.520) | 2.167(1.526-2.925) | 0.866(0.529-1.321) | 3.268(1.995-4.987) | 2.726(2.183-3.271) |
| Sao Tome and Principe | 12.865(10.147-15.253) | 25.030(19.743-29.677) | 27.847(19.068-34.698) | 24.254(16.607-30.221) | -0.116(-0.200--0.031) |
| Saudi Arabia | 693.696(476.880-1042.512) | 9.074(6.238-13.636) | 3409.208(2399.782-4434.321) | 12.365(8.704-16.083) | 0.571(0.358-0.786) |
| Senegal | 450.583(358.453-586.029) | 14.145(11.253-18.397) | 1180.485(875.802-1640.561) | 15.243(11.309-21.184) | 0.368(0.240-0.497) |
| Serbia | 427.457(325.567-576.256) | 6.353(4.839-8.564) | 914.726(724.201-1125.666) | 12.983(10.279-15.977) | 2.713(2.567-2.858) |
| Seychelles | 7.490(6.305-8.947) | 17.993(15.147-21.494) | 22.445(18.052-27.296) | 29.965(24.101-36.442) | 1.732(1.566-1.899) |
| Sierra Leone | 228.582(170.132-298.002) | 11.747(8.743-15.314) | 395.096(289.982-535.437) | 9.153(6.718-12.404) | -1.189(-1.374--1.005) |
| Singapore | 31.596(24.320-39.384) | 1.486(1.144-1.852) | 124.667(90.505-164.318) | 2.662(1.932-3.508) | 3.339(2.465-4.220) |
| Slovakia | 100.559(76.055-129.371) | 2.850(2.156-3.667) | 148.911(109.819-195.022) | 3.456(2.549-4.527) | 1.119(0.546-1.696) |
| Slovenia | 12.116(9.763-16.177) | 0.855(0.689-1.142) | 49.250(33.655-68.584) | 2.958(2.022-4.120) | 5.624(5.161-6.088) |
| Solomon Islands | 3.049(1.209-5.172) | 2.111(0.837-3.581) | 8.981(6.303-12.285) | 2.546(1.786-3.482) | 0.549(0.364-0.735) |
| Somalia | 154.995(102.602-225.674) | 4.834(3.200-7.038) | 409.098(268.307-653.226) | 4.637(3.041-7.404) | -0.159(-0.275--0.043) |
| South Africa | 1221.089(1005.006-1562.842) | 6.297(5.183-8.059) | 5546.756(4692.723-6577.329) | 15.017(12.705-17.807) | 2.222(1.773-2.673) |
| South Sudan | 173.320(115.564-261.384) | 6.688(4.460-10.087) | 312.103(213.386-436.659) | 7.457(5.099-10.433) | 0.040(-0.380-0.463) |
| Spain | 1204.613(915.376-1570.927) | 4.359(3.312-5.685) | 2908.076(2034.580-3973.950) | 7.915(5.537-10.816) | 2.117(1.889-2.345) |
| Sri Lanka | 1282.443(1025.361-1582.503) | 12.967(10.367-16.000) | 2503.668(1618.065-3495.800) | 16.289(10.527-22.744) | 0.634(0.449-0.820) |
| Sudan | 802.988(529.636-1517.052) | 8.947(5.901-16.903) | 2353.836(1694.625-3304.816) | 10.678(7.687-14.992) | 0.639(0.561-0.717) |
| Suriname | 20.114(15.956-25.256) | 9.324(7.396-11.708) | 73.387(50.818-100.426) | 18.846(13.051-25.790) | 2.653(2.416-2.890) |
| Sweden | 108.485(81.152-141.277) | 1.674(1.252-2.180) | 455.268(318.925-606.781) | 5.721(4.007-7.625) | 4.295(4.100-4.489) |
| Switzerland | 244.507(192.058-306.019) | 4.623(3.631-5.786) | 710.738(519.979-876.657) | 9.926(7.262-12.243) | 3.550(3.142-3.960) |
| Syrian Arab Republic | 573.080(417.936-828.839) | 10.681(7.789-15.447) | 1607.040(1107.501-2195.758) | 18.708(12.892-25.561) | 1.600(0.680-2.530) |
| Taiwan (Province of China) | 804.157(639.978-975.720) | 6.147(4.892-7.459) | 2737.375(2095.673-3376.761) | 13.972(10.697-17.236) | 3.186(2.804-3.569) |
| Tajikistan | 1.939(1.373-2.963) | 0.078(0.055-0.119) | 5.426(3.629-8.054) | 0.095(0.064-0.141) | -0.028(-0.371-0.316) |
| Thailand | 3536.212(2810.986-4629.516) | 10.395(8.263-13.609) | 15276.343(11172.497-19760.501) | 28.804(21.066-37.259) | 2.755(2.455-3.056) |
| Timor-Leste | 30.938(21.789-43.841) | 8.262(5.819-11.707) | 113.410(76.739-170.444) | 15.922(10.773-23.929) | 2.695(2.491-2.900) |
| Togo | 129.312(101.006-164.987) | 8.656(6.761-11.044) | 424.755(313.952-579.307) | 10.124(7.483-13.808) | 0.407(0.328-0.485) |
| Tokelau | 0.036(0.022-0.067) | 4.278(2.652-7.960) | 0.062(0.040-0.098) | 7.234(4.664-11.394) | 1.925(1.652-2.200) |
| Tonga | 0.729(0.473-1.066) | 1.604(1.039-2.343) | 1.805(1.156-2.551) | 3.183(2.039-4.498) | 2.062(1.862-2.263) |
| Trinidad and Tobago | 60.144(49.203-71.124) | 8.776(7.179-10.378) | 238.568(169.944-314.840) | 23.181(16.513-30.592) | 3.685(3.307-4.064) |
| Tunisia | 197.372(129.656-312.894) | 4.528(2.974-7.177) | 882.744(583.875-1289.275) | 10.691(7.071-15.614) | 2.722(2.510-2.935) |
| Turkmenistan | 6.895(5.202-8.801) | 0.380(0.287-0.485) | 29.526(21.048-41.142) | 0.923(0.658-1.287) | 2.529(2.034-3.026) |
| Tuvalu | 0.180(0.118-0.297) | 3.427(2.246-5.667) | 0.421(0.273-0.634) | 5.652(3.667-8.502) | 1.573(1.406-1.741) |
| Türkiye | 2995.474(2223.853-4412.201) | 9.728(7.222-14.329) | 8735.035(6172.159-11302.563) | 14.830(10.479-19.189) | 2.170(1.439-2.906) |
| Uganda | 311.669(209.753-444.234) | 4.457(2.999-6.352) | 876.665(622.342-1184.296) | 4.772(3.388-6.446) | -0.071(-0.214-0.072) |
| Ukraine | 9.379(7.562-11.461) | 0.025(0.020-0.030) | 182.241(125.611-252.996) | 0.526(0.363-0.731) | 13.871(11.805-15.975) |
| United Arab Emirates | 22.649(15.203-33.349) | 1.946(1.306-2.865) | 185.734(118.102-263.146) | 2.340(1.488-3.316) | 0.513(-0.043-1.071) |
| United Kingdom | 561.088(424.475-722.499) | 1.321(1.000-1.701) | 1328.360(955.074-1763.761) | 2.545(1.830-3.379) | 3.173(2.725-3.623) |
| United Republic of Tanzania | 388.174(281.704-536.400) | 3.557(2.581-4.915) | 1017.979(735.486-1360.050) | 3.690(2.666-4.930) | -0.137(-0.272--0.001) |
| United States of America | 11425.612(9348.094-13519.547) | 6.346(5.192-7.509) | 55557.205(45508.028-62182.833) | 22.105(18.107-24.741) | 4.470(4.283-4.657) |
| United States Virgin Islands | 5.050(3.893-6.617) | 7.849(6.050-10.284) | 11.653(7.939-15.448) | 17.217(11.730-22.825) | 2.773(2.531-3.016) |
| Uruguay | 182.562(166.304-196.004) | 8.852(8.064-9.504) | 394.751(306.037-481.703) | 15.776(12.230-19.250) | 2.151(1.899-2.402) |
| Uzbekistan | 24.942(15.056-44.584) | 0.243(0.146-0.434) | 151.044(109.120-201.311) | 0.699(0.505-0.931) | 2.423(1.631-3.220) |
| Vanuatu | 1.290(0.735-2.314) | 1.876(1.068-3.365) | 5.631(3.622-9.245) | 3.402(2.188-5.584) | 1.813(1.723-1.904) |
| Venezuela (Bolivarian Republic of) | 338.840(272.689-403.730) | 3.482(2.802-4.149) | 2790.910(1896.405-3747.979) | 15.612(10.608-20.965) | 4.364(3.933-4.797) |
| Viet Nam | 4349.287(3093.268-5597.493) | 12.585(8.950-16.197) | 11596.785(7744.266-14692.232) | 16.893(11.281-21.402) | 1.042(0.849-1.236) |
| Yemen | 248.154(146.686-482.116) | 4.748(2.807-9.225) | 783.994(506.743-1308.675) | 4.848(3.134-8.093) | -0.053(-0.221-0.116) |
| Zambia | 200.235(151.273-258.762) | 6.188(4.675-7.996) | 553.780(373.034-810.948) | 6.112(4.117-8.950) | -0.311(-0.475--0.147) |
| Zimbabwe | 316.108(237.000-426.324) | 7.317(5.486-9.868) | 829.367(602.455-1155.050) | 10.897(7.915-15.176) | 1.433(0.896-1.972) |

ASDR, age-standardized deaths rate; CI, credibility interval. EAPC, estimated annual percentage change. UI, uncertainty interval.

Supplementary Table S6. DALYs of chronic kidney disease due to hypertension between 1990 and 2021 in 204 countries and territories

|  | Rate per 100 000 (95% UI) | | | |  |
| --- | --- | --- | --- | --- | --- |
|  | 1990 |  | 2021 |  | 1990-2021 |
| Region | DALYs cases | Age-standardized DALY rate | DALYs cases | Age-standardized DALY rate | EAPC (95%CI) |
| Global | 4256733.709(3577599.278-5030814.990) | 138.428(116.343-163.601) | 10767724.382(9142005.282-12240814.073) | 204.884(173.951-232.914) | 1.319(1.269-1.369) |
| Afghanistan | 22698.691(15459.411-36858.658) | 519.361(353.721-843.350) | 37095.812(21808.147-63715.330) | 275.898(162.197-473.879) | -1.818(-2.022--1.614) |
| Albania | 751.497(600.935-926.097) | 40.419(32.321-49.809) | 1333.396(1010.815-1713.540) | 65.020(49.290-83.557) | 1.553(1.273-1.833) |
| Algeria | 16762.507(11278.229-28627.301) | 142.399(95.810-243.192) | 65384.498(49676.078-84902.895) | 235.627(179.019-305.966) | 1.720(1.440-2.000) |
| American Samoa | 19.856(14.756-27.398) | 80.793(60.040-111.482) | 72.030(52.708-95.404) | 235.161(172.080-311.473) | 3.710(3.506-3.914) |
| Andorra | 22.790(16.962-30.441) | 55.717(41.468-74.421) | 61.206(45.897-79.808) | 85.944(64.448-112.066) | 1.671(1.461-1.880) |
| Angola | 11029.993(7859.030-14822.739) | 244.554(174.248-328.646) | 32649.183(22252.902-45367.811) | 232.698(158.601-323.346) | -0.235(-0.410--0.059) |
| Antigua and Barbuda | 122.565(102.540-144.905) | 337.297(282.188-398.775) | 316.526(255.921-377.617) | 479.354(387.572-571.872) | 1.786(1.385-2.189) |
| Argentina | 54364.526(44486.449-64376.729) | 270.682(221.499-320.533) | 82132.330(67480.932-96255.932) | 258.475(212.366-302.923) | -0.014(-0.297-0.270) |
| Armenia | 526.595(351.833-712.323) | 25.162(16.812-34.037) | 1216.374(916.341-1516.160) | 54.493(41.052-67.923) | 2.650(2.489-2.812) |
| Australia | 5537.695(4725.834-6371.145) | 47.384(40.437-54.515) | 18228.721(14320.828-22174.468) | 93.224(73.239-113.403) | 2.561(2.283-2.840) |
| Austria | 3754.411(2996.775-4526.812) | 63.688(50.836-76.791) | 11739.726(9817.565-13683.228) | 162.433(135.838-189.324) | 4.906(4.326-5.489) |
| Azerbaijan | 1434.153(1063.125-1915.315) | 34.176(25.335-45.643) | 3306.248(2417.024-4408.782) | 44.359(32.429-59.151) | 0.729(0.663-0.794) |
| Bahamas | 311.975(253.801-375.135) | 209.322(170.290-251.700) | 1105.212(846.844-1421.666) | 405.286(310.541-521.331) | 2.687(2.488-2.888) |
| Bahrain | 390.181(280.498-522.643) | 127.358(91.556-170.594) | 2077.445(1571.232-2667.719) | 184.485(139.531-236.903) | 0.120(-0.298-0.540) |
| Bangladesh | 40818.565(32223.164-52268.341) | 83.710(66.083-107.191) | 88573.399(68244.883-121465.886) | 85.457(65.844-117.192) | 0.299(0.165-0.434) |
| Barbados | 407.997(343.900-479.473) | 242.120(204.082-284.536) | 922.416(676.363-1200.155) | 396.492(290.728-515.875) | 1.961(1.740-2.182) |
| Belarus | 1718.839(1208.570-2350.553) | 23.525(16.541-32.171) | 2853.999(2215.100-3624.740) | 39.099(30.346-49.658) | 1.613(1.354-1.873) |
| Belgium | 7065.063(5642.923-8288.337) | 94.111(75.167-110.406) | 11958.456(9503.518-14362.283) | 133.988(106.482-160.922) | 1.701(1.541-1.862) |
| Belize | 173.091(145.160-201.324) | 205.217(172.102-238.691) | 993.467(790.266-1206.536) | 382.066(303.919-464.008) | 2.515(2.159-2.872) |
| Benin | 6733.146(5472.097-8312.625) | 339.897(276.238-419.631) | 18252.556(13847.417-23535.983) | 305.080(231.451-393.389) | -0.394(-0.491--0.297) |
| Bermuda | 70.910(59.744-83.106) | 163.083(137.401-191.131) | 142.169(112.281-174.544) | 273.173(215.744-335.381) | 2.219(1.839-2.600) |
| Bhutan | 263.054(182.862-359.284) | 90.883(63.177-124.130) | 674.677(480.524-918.902) | 134.418(95.736-183.076) | 1.043(0.949-1.136) |
| Bolivia (Plurinational State of) | 8706.735(6681.869-11640.665) | 287.143(220.364-383.903) | 30527.588(21769.173-42429.797) | 421.050(300.250-585.211) | 1.358(1.279-1.438) |
| Bosnia and Herzegovina | 1200.125(957.921-1478.133) | 39.785(31.756-49.001) | 2308.628(1784.642-2867.066) | 87.521(67.657-108.692) | 2.850(2.266-3.439) |
| Botswana | 1065.252(754.759-1556.301) | 184.058(130.410-268.903) | 3244.400(2453.619-4613.685) | 219.773(166.206-312.527) | 0.489(0.314-0.664) |
| Brazil | 129286.257(108042.852-150352.448) | 158.992(132.867-184.898) | 343498.024(288050.212-401152.341) | 219.573(184.129-256.427) | 0.961(0.815-1.106) |
| Brunei Darussalam | 142.522(110.148-183.825) | 98.815(76.369-127.452) | 391.679(307.544-479.009) | 121.640(95.511-148.761) | 0.920(0.707-1.134) |
| Bulgaria | 3780.279(3059.378-4499.705) | 59.845(48.433-71.234) | 9468.265(7225.680-11978.051) | 172.381(131.552-218.074) | 4.161(3.782-4.540) |
| Burkina Faso | 14218.816(11153.665-17792.052) | 367.326(288.142-459.637) | 35821.387(27599.936-46475.207) | 359.822(277.238-466.838) | -0.052(-0.192-0.088) |
| Burundi | 3793.426(2660.116-5180.873) | 158.176(110.920-216.029) | 6624.700(4492.132-9968.045) | 111.419(75.552-167.650) | -1.678(-1.942--1.413) |
| Cabo Verde | 330.258(270.339-395.428) | 207.271(169.665-248.171) | 1080.612(733.099-1407.245) | 294.833(200.018-383.951) | 0.879(0.644-1.115) |
| Cambodia | 16101.715(12532.937-20902.827) | 351.610(273.679-456.451) | 40763.566(29622.231-55135.087) | 391.469(284.474-529.485) | 0.259(0.122-0.395) |
| Cameroon | 22732.300(17280.066-29475.193) | 505.182(384.017-655.030) | 66081.455(45704.674-94159.128) | 444.190(307.220-632.924) | -1.504(-2.079--0.926) |
| Canada | 18246.079(15621.515-20751.268) | 93.148(79.750-105.938) | 58134.629(48527.594-66297.035) | 198.817(165.961-226.731) | 2.891(2.696-3.087) |
| Central African Republic | 3991.602(3009.829-5203.220) | 323.600(244.008-421.826) | 7904.087(5490.061-11361.071) | 305.162(211.961-438.630) | -0.192(-0.319--0.065) |
| Chad | 6609.532(4930.298-9281.131) | 264.256(197.119-371.070) | 16903.442(12017.660-25650.517) | 248.588(176.736-377.225) | -0.307(-0.459--0.155) |
| Chile | 9733.870(8131.201-11367.860) | 121.506(101.500-141.903) | 28247.563(23042.562-33103.112) | 203.160(165.725-238.082) | 2.154(1.821-2.488) |
| China | 934658.710(765819.059-1136348.964) | 127.788(104.704-155.363) | 1622065.500(1293394.384-1981189.476) | 149.026(118.830-182.021) | 0.419(0.278-0.559) |
| Colombia | 19354.789(16241.113-22711.821) | 110.988(93.133-130.239) | 48790.916(38849.724-59245.349) | 141.419(112.605-171.721) | 1.036(0.889-1.183) |
| Comoros | 312.051(217.379-428.268) | 157.402(109.648-216.024) | 814.089(566.196-1111.634) | 189.136(131.543-258.263) | 0.453(0.274-0.634) |
| Congo | 4110.289(2876.680-5289.534) | 382.319(267.575-492.006) | 9888.632(6608.355-13855.182) | 340.429(227.501-476.982) | -0.601(-0.840--0.362) |
| Cook Islands | 7.889(6.062-10.200) | 76.497(58.779-98.912) | 17.693(13.377-22.700) | 140.816(106.463-180.667) | 2.126(1.962-2.289) |
| Costa Rica | 1597.026(1323.144-1890.896) | 98.366(81.497-116.467) | 8218.115(6454.979-9904.269) | 243.217(191.037-293.119) | 3.122(2.908-3.337) |
| Croatia | 2218.399(1809.822-2620.522) | 62.756(51.198-74.132) | 4598.940(3582.589-5675.284) | 135.147(105.280-166.777) | 3.713(3.336-4.091) |
| Cuba | 6945.332(5918.381-7980.473) | 96.529(82.256-110.916) | 20848.705(16853.286-25153.316) | 235.092(190.039-283.631) | 3.400(3.167-3.634) |
| Cyprus | 618.901(461.575-846.844) | 119.251(88.937-163.172) | 1163.614(893.609-1431.096) | 108.476(83.306-133.412) | -0.605(-0.779--0.431) |
| Czechia | 5096.928(4149.311-6115.459) | 70.297(57.228-84.345) | 6618.248(5263.507-7982.044) | 78.678(62.572-94.890) | 0.544(0.305-0.783) |
| Côte d'Ivoire | 13932.093(10539.616-17760.027) | 263.884(199.628-336.388) | 40041.957(28957.947-54401.907) | 297.108(214.866-403.658) | 0.175(0.048-0.303) |
| Democratic People's Republic of Korea | 17611.092(12815.213-23455.305) | 137.564(100.102-183.215) | 35477.339(26550.047-47133.746) | 179.269(134.159-238.170) | 0.868(0.804-0.932) |
| Democratic Republic of the Congo | 42685.550(31225.215-56574.052) | 258.740(189.272-342.925) | 106998.436(71813.220-149286.062) | 254.088(170.534-354.508) | -0.207(-0.323--0.092) |
| Denmark | 2082.322(1650.762-2528.327) | 53.483(42.398-64.938) | 5312.307(4420.263-6173.052) | 116.560(96.987-135.446) | 2.498(2.385-2.612) |
| Djibouti | 160.111(111.124-227.153) | 84.282(58.495-119.572) | 960.257(665.311-1393.989) | 131.485(91.099-190.875) | 1.433(1.307-1.559) |
| Dominica | 129.482(103.036-157.261) | 326.563(259.865-396.623) | 272.704(203.722-359.663) | 572.295(427.529-754.785) | 2.072(1.970-2.175) |
| Dominican Republic | 5355.546(4307.877-6829.034) | 146.779(118.065-187.162) | 19983.493(13344.751-26484.715) | 280.580(187.368-371.861) | 2.772(2.577-2.968) |
| Ecuador | 8640.034(7181.686-10219.497) | 172.090(143.043-203.549) | 43319.547(29661.519-64212.450) | 378.366(259.072-560.850) | 2.612(1.696-3.536) |
| Egypt | 80149.745(59858.227-125268.964) | 292.404(218.376-457.010) | 257945.116(192721.661-326953.007) | 436.624(326.220-553.434) | 1.479(1.347-1.611) |
| El Salvador | 4237.427(3334.249-5995.229) | 166.155(130.740-235.081) | 22410.074(15715.350-29431.195) | 550.866(386.302-723.454) | 4.103(3.631-4.577) |
| Equatorial Guinea | 613.045(431.926-814.921) | 335.322(236.254-445.744) | 1882.991(1121.581-2858.432) | 253.710(151.119-385.139) | -0.717(-1.217--0.214) |
| Eritrea | 1648.502(1108.229-2437.060) | 114.547(77.006-169.341) | 4112.590(2673.363-6628.720) | 121.629(79.064-196.043) | 0.102(0.059-0.146) |
| Estonia | 740.859(592.285-899.969) | 66.794(53.399-81.139) | 2286.325(1902.357-2676.055) | 221.859(184.600-259.677) | 3.584(2.815-4.358) |
| Eswatini | 879.954(650.296-1147.119) | 267.249(197.500-348.389) | 2538.022(1582.266-3676.988) | 409.207(255.110-592.843) | 1.647(0.962-2.336) |
| Ethiopia | 76020.506(56971.358-97231.924) | 360.262(269.988-460.784) | 95954.552(74046.650-121570.547) | 185.455(143.113-234.964) | -2.653(-2.963--2.342) |
| Fiji | 391.682(281.886-537.548) | 98.001(70.530-134.498) | 1074.173(776.039-1452.909) | 187.189(135.235-253.189) | 1.826(1.668-1.985) |
| Finland | 934.735(686.908-1247.571) | 24.985(18.361-33.347) | 2021.190(1531.219-2586.745) | 46.068(34.900-58.958) | 2.424(2.206-2.643) |
| France | 30197.352(24528.825-36033.559) | 72.539(58.922-86.558) | 60426.352(50881.149-70146.038) | 119.330(100.480-138.524) | 1.977(1.720-2.236) |
| Gabon | 1960.797(1470.813-2496.418) | 412.491(309.413-525.169) | 4576.169(2509.893-6516.438) | 462.690(253.772-658.868) | 0.365(0.281-0.449) |
| Gambia | 1098.528(797.412-1454.385) | 261.673(189.946-346.439) | 3921.480(2862.722-5270.018) | 347.792(253.892-467.392) | 0.844(0.694-0.994) |
| Georgia | 1361.078(950.508-1850.167) | 36.527(25.509-49.653) | 1896.570(1432.421-2443.967) | 70.821(53.489-91.261) | 2.322(2.118-2.526) |
| Germany | 46161.159(36877.192-54414.218) | 73.728(58.900-86.909) | 114716.962(87720.241-141687.807) | 165.239(126.353-204.088) | 3.854(3.472-4.238) |
| Ghana | 11625.600(8269.406-16967.371) | 173.208(123.205-252.794) | 48321.283(35553.054-63408.621) | 269.471(198.267-353.608) | 1.551(1.364-1.738) |
| Greece | 8835.258(7024.714-10715.920) | 116.580(92.690-141.394) | 18404.039(15521.695-21144.563) | 222.550(187.696-255.690) | 2.526(1.603-3.457) |
| Greenland | 32.671(27.078-40.160) | 86.735(71.886-106.617) | 72.864(54.720-93.005) | 178.887(134.342-228.335) | 2.781(2.613-2.950) |
| Grenada | 191.293(158.443-229.062) | 426.490(353.250-510.697) | 456.183(356.348-563.393) | 629.028(491.366-776.859) | 1.721(1.391-2.051) |
| Guam | 145.643(118.962-187.169) | 176.190(143.912-226.424) | 429.037(359.708-495.525) | 387.528(324.907-447.583) | 3.275(3.070-3.480) |
| Guatemala | 5631.224(4679.651-6728.001) | 162.119(134.724-193.694) | 29177.456(22115.632-36578.746) | 318.795(241.637-399.661) | 3.057(2.601-3.514) |
| Guinea | 9502.331(7205.599-12953.818) | 351.047(266.198-478.557) | 18329.399(13515.942-26441.832) | 307.267(226.576-443.261) | -0.414(-0.604--0.223) |
| Guinea-Bissau | 1922.279(1424.591-2417.244) | 458.764(339.988-576.891) | 3364.544(2463.307-4496.837) | 355.891(260.561-475.661) | -0.940(-1.007--0.873) |
| Guyana | 955.404(785.710-1143.855) | 240.005(197.376-287.345) | 2684.778(1868.874-3622.187) | 554.675(386.109-748.344) | 3.918(3.519-4.320) |
| Haiti | 7359.813(5074.958-12842.007) | 242.547(167.248-423.215) | 17491.836(9782.125-35753.637) | 241.058(134.809-492.727) | 0.107(0.000-0.214) |
| Honduras | 1690.010(1312.222-2213.643) | 84.648(65.726-110.875) | 8575.857(6284.416-11138.540) | 148.895(109.111-193.389) | 2.059(1.919-2.198) |
| Hungary | 4598.660(3829.002-5387.586) | 61.375(51.103-71.904) | 8595.129(6744.788-10458.544) | 111.308(87.346-135.439) | 2.747(2.394-3.101) |
| Iceland | 74.105(60.886-87.665) | 43.750(35.946-51.756) | 196.563(160.421-229.845) | 75.231(61.398-87.968) | 1.993(1.859-2.127) |
| India | 410258.881(328550.218-514050.511) | 92.683(74.224-116.130) | 1098641.585(859840.688-1367444.424) | 120.212(94.082-149.624) | 0.765(0.658-0.871) |
| Indonesia | 294034.667(237205.622-364696.435) | 301.512(243.238-373.971) | 772005.029(607610.440-969585.612) | 409.126(322.004-513.834) | 1.046(1.009-1.083) |
| Iran (Islamic Republic of) | 39439.506(29607.356-54275.377) | 154.117(115.696-212.091) | 127909.971(101747.509-147747.580) | 214.970(171.000-248.310) | 1.235(1.078-1.392) |
| Iraq | 20303.821(15096.593-26572.696) | 250.082(185.945-327.296) | 58592.612(37994.609-78849.439) | 248.406(161.080-334.286) | -0.175(-0.411-0.061) |
| Ireland | 1612.173(1261.110-1984.917) | 70.917(55.475-87.314) | 3311.037(2618.846-4132.679) | 91.471(72.348-114.169) | 0.981(0.789-1.172) |
| Israel | 4342.629(3518.948-5240.667) | 146.971(119.095-177.364) | 11251.718(8935.790-13561.148) | 180.881(143.650-218.007) | 1.204(0.910-1.500) |
| Italy | 43017.257(35314.163-50145.326) | 99.626(81.786-116.134) | 82501.205(64588.472-99599.428) | 167.237(130.927-201.897) | 1.576(1.332-1.820) |
| Jamaica | 3153.618(2646.208-3654.303) | 247.727(207.868-287.058) | 7260.289(5180.570-9675.155) | 365.845(261.048-487.530) | 0.821(0.238-1.408) |
| Japan | 53758.181(43891.739-63802.316) | 58.139(47.469-69.002) | 126888.995(99682.498-157800.860) | 119.185(93.630-148.220) | 2.590(2.336-2.846) |
| Jordan | 2290.508(1764.963-3104.367) | 139.743(107.680-189.396) | 11893.290(9004.046-15669.593) | 160.731(121.685-211.766) | 0.258(0.112-0.406) |
| Kazakhstan | 3488.531(2584.184-4514.443) | 35.781(26.505-46.303) | 5713.331(4322.080-7239.414) | 46.710(35.336-59.187) | 0.561(0.404-0.718) |
| Kenya | 8222.544(5964.794-12841.194) | 87.891(63.758-137.260) | 32196.004(23667.496-43510.483) | 126.448(92.952-170.884) | 1.404(1.328-1.481) |
| Kiribati | 40.612(31.303-52.852) | 107.572(82.915-139.993) | 102.830(65.825-153.448) | 151.855(97.207-226.605) | 0.822(0.611-1.035) |
| Kuwait | 1279.836(1037.684-1504.745) | 123.944(100.493-145.725) | 2907.809(2186.549-3632.746) | 81.804(61.513-102.198) | -1.719(-2.009--1.428) |
| Kyrgyzstan | 841.202(587.325-1133.319) | 35.758(24.966-48.175) | 1585.637(1191.116-2056.837) | 39.246(29.481-50.909) | -0.222(-0.503-0.060) |
| Lao People's Democratic Republic | 12050.542(8578.994-16207.628) | 633.901(451.286-852.579) | 23576.343(16075.301-32310.942) | 537.532(366.511-736.678) | -0.712(-0.780--0.644) |
| Latvia | 576.070(435.333-740.219) | 30.229(22.844-38.843) | 1003.506(783.232-1243.169) | 67.656(52.805-83.814) | 2.821(2.423-3.221) |
| Lebanon | 4233.365(3049.592-5836.857) | 256.126(184.506-353.140) | 12480.918(9698.918-15441.672) | 322.156(250.347-398.578) | 0.988(0.754-1.224) |
| Lesotho | 1435.025(1072.999-1892.277) | 206.405(154.334-272.173) | 4256.507(2871.620-5937.494) | 411.034(277.301-573.360) | 2.943(2.453-3.436) |
| Liberia | 4512.217(3376.396-6074.595) | 412.282(308.502-555.037) | 9460.313(6749.411-13031.991) | 354.393(252.840-488.191) | -1.012(-1.409--0.614) |
| Libya | 3734.198(2726.465-5105.142) | 195.843(142.992-267.744) | 16305.722(10461.000-22744.629) | 340.907(218.710-475.527) | 2.189(2.001-2.376) |
| Lithuania | 846.076(638.126-1092.678) | 32.964(24.862-42.571) | 1564.731(1215.719-1954.917) | 71.367(55.449-89.163) | 2.175(1.998-2.353) |
| Luxembourg | 212.509(171.738-253.928) | 72.543(58.625-86.682) | 523.710(405.827-649.047) | 102.820(79.676-127.427) | 1.418(1.275-1.561) |
| Madagascar | 5506.507(3971.908-7953.606) | 106.415(76.758-153.705) | 11898.980(8046.945-16551.722) | 87.405(59.109-121.582) | -0.546(-0.748--0.343) |
| Malawi | 5921.727(4254.212-7916.301) | 139.914(100.515-187.040) | 13789.149(10532.399-17803.024) | 155.687(118.916-201.005) | 0.152(-0.114-0.419) |
| Malaysia | 26895.096(22419.304-31496.599) | 288.452(240.449-337.804) | 88129.270(71442.632-103799.616) | 408.819(331.412-481.512) | 1.018(0.865-1.171) |
| Maldives | 527.621(412.983-668.940) | 561.811(439.744-712.288) | 1077.586(852.759-1312.379) | 278.517(220.407-339.202) | -2.639(-2.827--2.450) |
| Mali | 13028.383(10074.246-16950.728) | 350.511(271.034-456.036) | 28737.210(21761.009-39073.348) | 291.126(220.452-395.837) | -0.412(-0.594--0.230) |
| Malta | 185.087(146.732-221.978) | 72.487(57.465-86.934) | 476.242(374.134-584.816) | 132.931(104.430-163.237) | 1.901(1.768-2.033) |
| Marshall Islands | 20.988(13.583-34.898) | 112.563(72.848-187.167) | 65.964(25.585-159.217) | 198.680(77.062-479.552) | 1.898(1.838-1.957) |
| Mauritania | 3685.085(2790.246-4797.619) | 400.251(303.059-521.087) | 7126.214(4850.726-10207.683) | 345.252(235.009-494.543) | -0.678(-0.874--0.483) |
| Mauritius | 3751.965(3219.577-4243.856) | 564.090(484.048-638.043) | 15073.908(12773.065-17236.050) | 1545.948(1309.978-1767.693) | 3.578(3.164-3.993) |
| Mexico | 72317.061(60841.719-84548.555) | 172.086(144.779-201.192) | 359583.773(277305.632-452834.856) | 417.513(321.980-525.787) | 3.537(2.899-4.180) |
| Micronesia (Federated States of) | 58.349(39.234-83.476) | 126.891(85.322-181.535) | 133.251(91.257-187.403) | 217.968(149.275-306.548) | 1.787(1.579-1.995) |
| Monaco | 20.694(16.140-25.922) | 80.936(63.124-101.382) | 40.119(31.435-49.243) | 129.028(101.099-158.372) | 1.587(1.367-1.808) |
| Mongolia | 438.624(327.685-566.857) | 42.889(32.041-55.427) | 829.457(624.947-1092.870) | 40.989(30.883-54.006) | -0.394(-0.533--0.254) |
| Montenegro | 558.354(436.470-689.768) | 135.630(106.023-167.552) | 1112.220(805.053-1499.132) | 237.821(172.141-320.553) | 2.019(1.744-2.294) |
| Morocco | 14482.823(9930.606-25751.607) | 112.469(77.118-199.979) | 54151.344(38931.140-72483.335) | 223.203(160.468-298.765) | 2.806(2.521-3.090) |
| Mozambique | 5565.938(4102.725-7980.442) | 96.143(70.868-137.849) | 15321.764(10928.169-21816.700) | 115.041(82.053-163.807) | 1.059(0.867-1.250) |
| Myanmar | 94508.802(68385.477-125198.711) | 443.137(320.649-587.038) | 148324.558(110912.810-192001.445) | 416.040(311.103-538.551) | -0.474(-0.569--0.380) |
| Namibia | 1155.581(855.554-1682.858) | 179.892(133.186-261.974) | 2956.167(2110.353-4173.710) | 217.400(155.198-306.939) | 0.373(0.078-0.670) |
| Nauru | 6.845(4.463-9.907) | 137.049(89.358-198.348) | 12.138(8.225-17.373) | 205.893(139.514-294.704) | 1.143(1.035-1.250) |
| Nepal | 8957.733(6804.891-11546.058) | 98.217(74.612-126.597) | 25949.149(19392.061-34045.890) | 138.949(103.838-182.304) | 1.159(0.770-1.551) |
| Netherlands | 7275.184(5950.194-8702.608) | 65.608(53.660-78.481) | 19458.410(16207.959-22310.648) | 143.927(119.885-165.025) | 3.335(3.113-3.556) |
| New Zealand | 1349.058(1111.284-1573.192) | 58.245(47.979-67.922) | 4270.064(3489.818-5015.741) | 110.451(90.269-129.739) | 2.229(1.921-2.538) |
| Nicaragua | 2647.744(2143.741-3485.691) | 161.718(130.934-212.897) | 15271.275(11446.766-19746.822) | 375.242(281.267-485.214) | 3.197(2.840-3.556) |
| Niger | 7723.173(5775.621-11111.031) | 242.818(181.587-349.333) | 20219.741(14044.841-32668.207) | 213.933(148.600-345.643) | -0.353(-0.489--0.216) |
| Nigeria | 108626.980(84158.475-140303.896) | 261.608(202.680-337.896) | 230602.526(172127.758-310380.512) | 223.034(166.478-300.194) | -0.604(-0.717--0.491) |
| Niue | 2.178(1.659-2.872) | 169.502(129.103-223.483) | 3.244(1.957-4.963) | 283.701(171.104-433.946) | 1.655(1.482-1.828) |
| North Macedonia | 1016.995(795.389-1271.140) | 78.510(61.402-98.129) | 2002.799(1512.524-2631.120) | 115.777(87.435-152.099) | 1.469(1.255-1.682) |
| Northern Mariana Islands | 15.329(11.407-20.355) | 53.032(39.461-70.419) | 41.452(31.283-55.867) | 123.753(93.394-166.791) | 3.198(2.846-3.550) |
| Norway | 1747.546(1426.861-2092.895) | 55.768(45.534-66.789) | 3878.498(3140.525-4600.323) | 92.862(75.193-110.145) | 2.367(2.089-2.646) |
| Oman | 1128.020(786.115-1670.037) | 114.857(80.043-170.046) | 4955.553(3711.868-6591.876) | 154.286(115.565-205.231) | 1.169(1.003-1.335) |
| Pakistan | 59848.926(46271.497-77233.240) | 119.307(92.240-153.961) | 188167.588(142400.745-245723.656) | 148.949(112.721-194.510) | 0.396(0.208-0.584) |
| Palau | 10.127(7.396-13.183) | 112.189(81.932-146.043) | 30.785(20.752-42.627) | 225.226(151.822-311.863) | 2.471(2.390-2.553) |
| Palestine | 2365.253(1668.079-3203.986) | 277.176(195.476-375.464) | 6139.625(4760.496-7651.282) | 225.081(174.521-280.499) | -0.733(-0.838--0.628) |
| Panama | 1258.690(1054.424-1493.932) | 96.679(80.990-114.748) | 7211.479(5354.418-9038.481) | 258.961(192.275-324.568) | 3.570(3.199-3.942) |
| Papua New Guinea | 1059.017(748.342-1436.304) | 53.634(37.900-72.742) | 3472.597(2637.497-4567.283) | 62.428(47.415-82.108) | 0.382(0.277-0.486) |
| Paraguay | 2712.570(2179.951-3369.478) | 137.446(110.458-170.731) | 10442.001(7509.787-14203.510) | 232.139(166.952-315.762) | 1.686(1.553-1.819) |
| Peru | 22403.219(17710.293-27706.930) | 203.953(161.230-252.237) | 67774.562(46022.290-88846.956) | 285.243(193.694-373.931) | 1.044(0.852-1.237) |
| Philippines | 121248.654(103737.338-142773.769) | 391.682(335.114-461.217) | 450520.008(360290.240-546765.613) | 657.964(526.188-798.527) | 2.135(1.958-2.313) |
| Poland | 22163.136(18223.558-25968.529) | 86.160(70.845-100.954) | 21660.722(17184.407-26144.493) | 70.926(56.269-85.608) | 0.732(0.081-1.386) |
| Portugal | 6000.767(4776.291-7063.014) | 83.855(66.744-98.699) | 13229.686(10398.095-16395.232) | 152.221(119.641-188.644) | 2.258(1.911-2.607) |
| Puerto Rico | 5815.469(4808.983-6921.767) | 255.037(210.898-303.554) | 10824.657(8993.629-12850.543) | 409.891(340.556-486.604) | 2.064(1.682-2.447) |
| Qatar | 255.633(157.204-386.518) | 87.618(53.881-132.478) | 1912.902(1421.998-2587.260) | 80.375(59.749-108.710) | -1.223(-1.701--0.743) |
| Republic of Korea | 26969.998(23662.088-30073.398) | 95.239(83.558-106.198) | 53092.837(41528.007-64558.244) | 122.936(96.158-149.485) | 1.197(0.751-1.645) |
| Republic of Moldova | 909.218(677.266-1182.032) | 31.724(23.631-41.242) | 1627.863(1250.890-2095.206) | 56.187(43.176-72.318) | 1.501(1.328-1.674) |
| Romania | 7102.474(5909.162-8402.998) | 44.735(37.219-52.926) | 9074.802(7396.820-10932.120) | 60.914(49.651-73.381) | 1.510(1.058-1.965) |
| Russian Federation | 42268.898(34361.989-50472.398) | 39.952(32.478-47.705) | 55186.413(44311.039-67240.595) | 49.689(39.897-60.542) | 0.221(-0.211-0.655) |
| Rwanda | 5893.254(4461.712-7626.681) | 193.176(146.251-249.997) | 9729.217(6664.034-13721.018) | 142.408(97.542-200.836) | -1.998(-2.535--1.458) |
| Saint Kitts and Nevis | 109.673(89.883-129.634) | 474.730(389.065-561.131) | 249.139(184.503-318.885) | 558.818(413.838-715.257) | 1.124(0.666-1.584) |
| Saint Lucia | 225.737(186.424-263.803) | 323.438(267.110-377.979) | 649.293(496.899-812.242) | 478.991(366.569-599.201) | 1.812(1.474-2.151) |
| Saint Vincent and the Grenadines | 137.434(115.221-162.502) | 246.520(206.676-291.487) | 388.865(309.124-463.111) | 483.837(384.621-576.216) | 2.854(2.519-3.191) |
| Samoa | 91.962(64.285-127.599) | 118.911(83.123-164.991) | 205.545(150.674-270.526) | 182.386(133.698-240.045) | 1.360(1.293-1.427) |
| San Marino | 9.369(7.279-11.510) | 52.720(40.961-64.766) | 19.779(14.622-25.498) | 74.634(55.175-96.215) | 1.885(1.608-2.162) |
| Sao Tome and Principe | 274.696(217.277-327.096) | 534.459(422.742-636.411) | 652.313(454.257-850.156) | 568.143(395.643-740.458) | 0.050(-0.074-0.175) |
| Saudi Arabia | 17751.766(12309.629-25289.035) | 232.201(161.015-330.791) | 101616.882(69144.241-136060.190) | 368.567(250.788-493.494) | 1.132(0.943-1.321) |
| Senegal | 11130.192(8730.810-14532.981) | 349.409(274.085-456.232) | 27678.788(20216.486-38803.667) | 357.411(261.052-501.064) | 0.174(0.039-0.309) |
| Serbia | 9771.337(7780.129-12731.379) | 145.224(115.630-189.217) | 16766.594(13695.544-20158.232) | 237.971(194.383-286.109) | 1.632(1.528-1.737) |
| Seychelles | 196.000(165.471-233.262) | 470.842(397.504-560.355) | 557.213(445.256-673.241) | 743.906(594.438-898.808) | 1.625(1.495-1.755) |
| Sierra Leone | 5402.004(3978.189-7060.183) | 277.601(204.433-362.813) | 10170.787(7282.703-13959.884) | 235.620(168.714-323.400) | -0.770(-0.881--0.659) |
| Singapore | 872.814(716.174-1048.285) | 41.050(33.683-49.302) | 2686.628(2170.338-3223.728) | 57.359(46.336-68.826) | 2.236(1.590-2.885) |
| Slovakia | 2750.799(2182.921-3385.775) | 77.969(61.873-95.967) | 3548.801(2796.806-4382.087) | 82.370(64.916-101.711) | 0.460(0.017-0.905) |
| Slovenia | 446.228(360.373-545.946) | 31.497(25.436-38.535) | 1049.929(802.161-1335.491) | 63.065(48.183-80.218) | 2.840(2.652-3.029) |
| Solomon Islands | 138.131(67.806-223.029) | 95.629(46.943-154.404) | 399.809(293.228-525.723) | 113.317(83.109-149.005) | 0.507(0.364-0.650) |
| Somalia | 4601.113(3066.020-6627.837) | 143.499(95.622-206.708) | 12381.484(8158.903-19902.528) | 140.346(92.482-225.598) | -0.164(-0.269--0.059) |
| South Africa | 34586.705(28900.330-42070.199) | 178.361(149.037-216.953) | 136842.534(114755.519-162350.670) | 370.482(310.684-439.541) | 1.796(1.388-2.205) |
| South Sudan | 4137.187(2799.106-6096.717) | 159.654(108.018-235.273) | 7963.221(5557.019-11036.012) | 190.272(132.779-263.693) | 0.299(-0.188-0.787) |
| Spain | 24420.837(19671.956-29290.436) | 88.369(71.185-105.990) | 43174.040(33209.323-53360.697) | 117.506(90.385-145.231) | 1.156(0.964-1.348) |
| Sri Lanka | 36574.136(30003.186-44754.806) | 369.793(303.356-452.506) | 62659.800(40819.427-85985.383) | 407.673(265.576-559.432) | 0.194(0.015-0.373) |
| Sudan | 20596.156(14095.385-35721.145) | 229.478(157.048-397.998) | 57399.198(39820.168-78167.659) | 260.385(180.640-354.599) | 0.455(0.328-0.581) |
| Suriname | 548.224(435.289-681.119) | 254.131(201.780-315.735) | 1824.098(1302.077-2473.232) | 468.445(334.385-635.149) | 2.240(2.031-2.450) |
| Sweden | 3120.060(2439.430-3771.658) | 48.144(37.641-58.198) | 7330.538(5655.232-9052.138) | 92.113(71.062-113.746) | 2.388(2.251-2.524) |
| Switzerland | 4883.795(3998.048-5764.472) | 92.335(75.588-108.985) | 10290.438(8142.222-12057.095) | 143.717(113.715-168.390) | 2.077(1.819-2.336) |
| Syrian Arab Republic | 13463.204(9897.398-18612.922) | 250.917(184.460-346.893) | 35184.500(25113.694-48779.510) | 409.582(292.348-567.842) | 1.267(0.422-2.118) |
| Taiwan (Province of China) | 21269.951(18119.510-24998.228) | 162.591(138.508-191.090) | 52915.832(43490.028-62089.273) | 270.100(221.987-316.924) | 2.265(1.964-2.568) |
| Tajikistan | 592.775(413.913-804.208) | 23.763(16.593-32.239) | 1382.355(960.156-1904.092) | 24.231(16.831-33.377) | -0.149(-0.265--0.033) |
| Thailand | 106892.262(85798.581-136612.315) | 314.225(252.217-401.591) | 338364.218(254302.819-429281.510) | 638.001(479.499-809.430) | 1.548(1.193-1.904) |
| Timor-Leste | 1079.872(750.550-1509.255) | 288.367(200.425-403.028) | 3127.162(2147.287-4674.424) | 439.021(301.457-656.241) | 1.764(1.546-1.982) |
| Togo | 3524.807(2763.490-4433.983) | 235.937(184.977-296.794) | 11831.878(8682.607-16278.097) | 282.012(206.949-387.987) | 0.510(0.451-0.569) |
| Tokelau | 1.094(0.756-1.798) | 130.834(90.402-214.990) | 1.635(1.162-2.319) | 190.200(135.191-269.806) | 1.273(1.092-1.454) |
| Tonga | 31.186(22.964-41.742) | 68.557(50.483-91.762) | 58.331(43.190-77.021) | 102.845(76.151-135.799) | 1.174(1.077-1.271) |
| Trinidad and Tobago | 1549.812(1306.029-1813.628) | 226.141(190.570-264.636) | 5580.210(3954.448-7476.348) | 542.214(384.243-726.457) | 3.262(2.927-3.598) |
| Tunisia | 4693.824(3366.331-7176.833) | 107.671(77.220-164.629) | 16925.065(11594.285-23774.713) | 204.978(140.417-287.933) | 1.992(1.784-2.201) |
| Turkmenistan | 658.043(489.145-878.566) | 36.276(26.965-48.432) | 1770.326(1345.776-2301.740) | 55.360(42.084-71.978) | 1.310(1.141-1.480) |
| Tuvalu | 6.579(4.796-10.006) | 125.572(91.542-190.988) | 13.286(9.129-18.195) | 178.236(122.464-244.083) | 1.104(1.008-1.201) |
| Türkiye | 67462.254(51794.581-94678.518) | 219.095(168.212-307.485) | 163209.639(121260.768-207806.278) | 277.097(205.876-352.813) | 1.341(0.754-1.930) |
| Uganda | 7693.111(5459.569-10560.863) | 110.009(78.070-151.018) | 21656.773(15174.674-29389.050) | 117.882(82.599-159.971) | -0.137(-0.292-0.019) |
| Ukraine | 7820.395(5249.321-10959.485) | 20.765(13.938-29.100) | 14403.497(10827.305-18418.624) | 41.589(31.263-53.183) | 2.700(2.410-2.990) |
| United Arab Emirates | 814.110(575.428-1143.549) | 69.949(49.441-98.255) | 6955.672(4831.643-9244.930) | 87.646(60.882-116.493) | 0.494(0.080-0.909) |
| United Kingdom | 21725.260(17098.034-26610.729) | 51.160(40.263-62.665) | 37384.325(29985.630-45465.249) | 71.626(57.451-87.109) | 1.476(1.302-1.651) |
| United Republic of Tanzania | 9838.095(7337.691-13462.777) | 90.140(67.231-123.351) | 24391.102(18291.793-32374.270) | 88.407(66.300-117.342) | -0.300(-0.417--0.182) |
| United States of America | 235546.605(200938.175-270254.265) | 130.824(111.602-150.100) | 958825.490(847457.673-1050654.254) | 381.499(337.188-418.036) | 3.816(3.645-3.986) |
| United States Virgin Islands | 146.255(113.545-186.426) | 227.294(176.460-289.724) | 256.530(179.078-345.067) | 379.020(264.586-509.832) | 1.966(1.745-2.188) |
| Uruguay | 3459.204(3213.744-3710.576) | 167.727(155.825-179.915) | 6090.469(5015.027-7229.159) | 243.396(200.418-288.902) | 1.410(1.199-1.620) |
| Uzbekistan | 3409.057(2457.730-4520.690) | 33.152(23.901-43.962) | 10532.045(8050.500-13481.293) | 48.708(37.232-62.348) | 0.942(0.715-1.169) |
| Vanuatu | 54.148(36.214-85.924) | 78.727(52.653-124.928) | 214.486(148.627-323.790) | 129.565(89.781-195.592) | 1.526(1.446-1.607) |
| Venezuela (Bolivarian Republic of) | 9941.512(8227.708-11714.283) | 102.161(84.550-120.379) | 64036.430(44026.666-84825.633) | 358.205(246.275-474.495) | 3.499(3.112-3.888) |
| Viet Nam | 117738.543(83096.476-152340.465) | 340.680(240.442-440.802) | 281353.883(191131.833-367027.935) | 409.844(278.419-534.645) | 0.715(0.499-0.931) |
| Yemen | 6857.946(4220.290-12212.637) | 131.218(80.750-233.673) | 19984.365(13500.175-31827.864) | 123.579(83.482-196.816) | -0.360(-0.538--0.183) |
| Zambia | 5166.887(3904.946-6670.296) | 159.671(120.673-206.130) | 14990.354(9825.956-23025.638) | 165.437(108.442-254.117) | -0.195(-0.354--0.036) |
| Zimbabwe | 8017.795(6144.532-10865.855) | 185.580(142.222-251.501) | 23312.133(16916.512-32499.607) | 306.290(222.260-427.001) | 1.842(1.333-2.352) |

CI, credibility interval. DALYs, disability adjusted life years. EAPC, estimated annual percentage change. UI, uncertainty interval.
