# Supplementary material for: Global burden and risk factors of chronic kidney disease due to hypertension in adults aged 20 plus years, 1990–2021
Source: Front Public Health. 2025 May 7;13:1503837. doi: 10.3389/fpubh.2025.1503837 (PMC12092432; doi:10.3389/fpubh.2025.1503837)
Supplement: Supplementary file 2 [file Data_Sheet_2.docx]

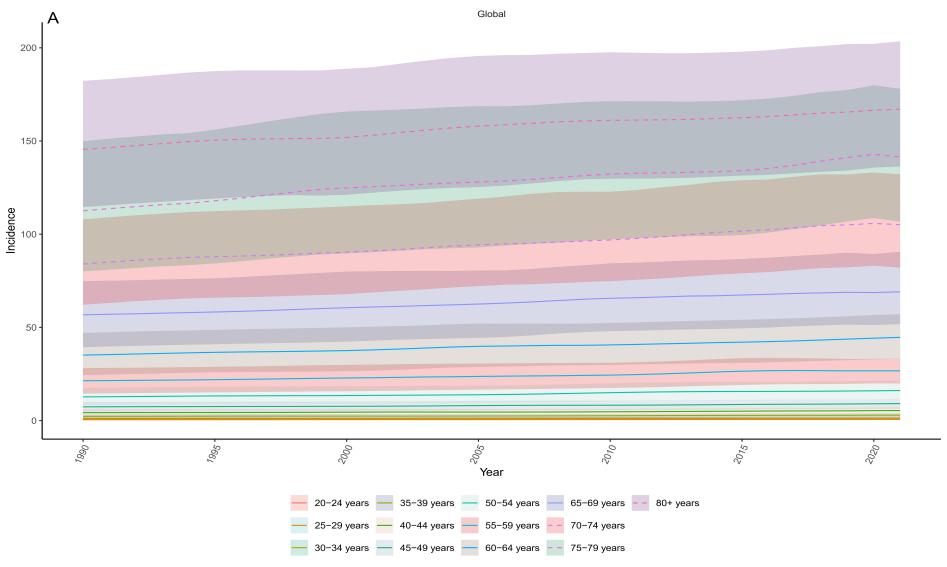

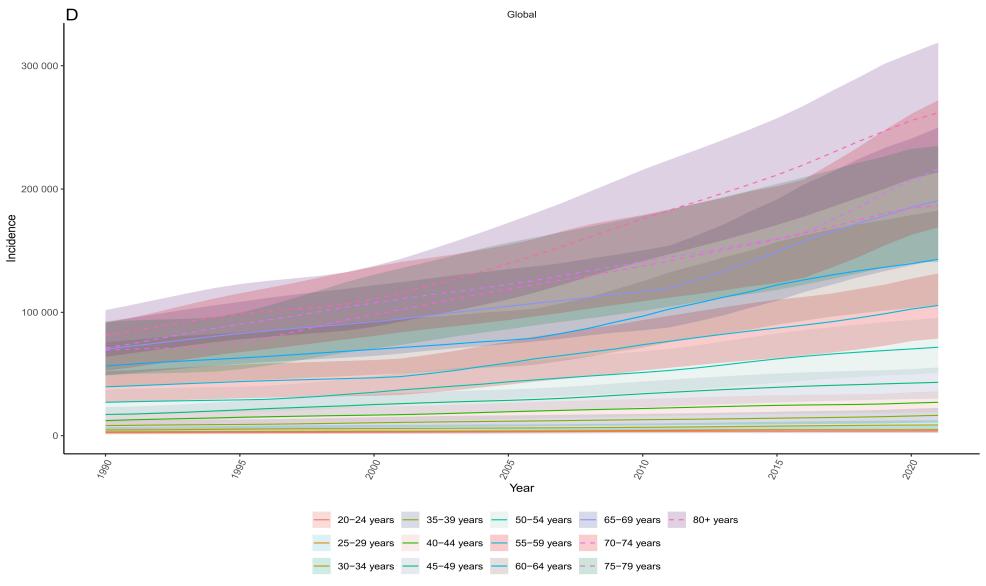


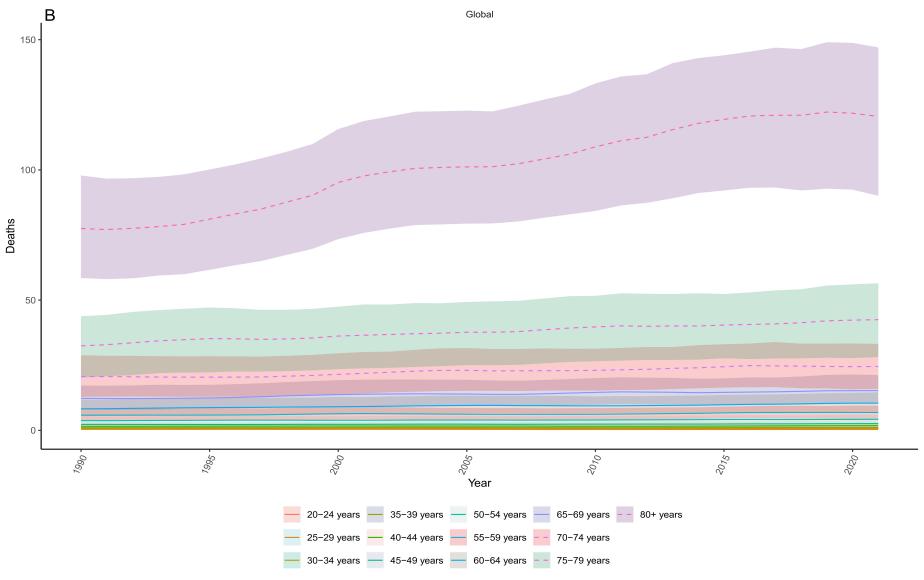

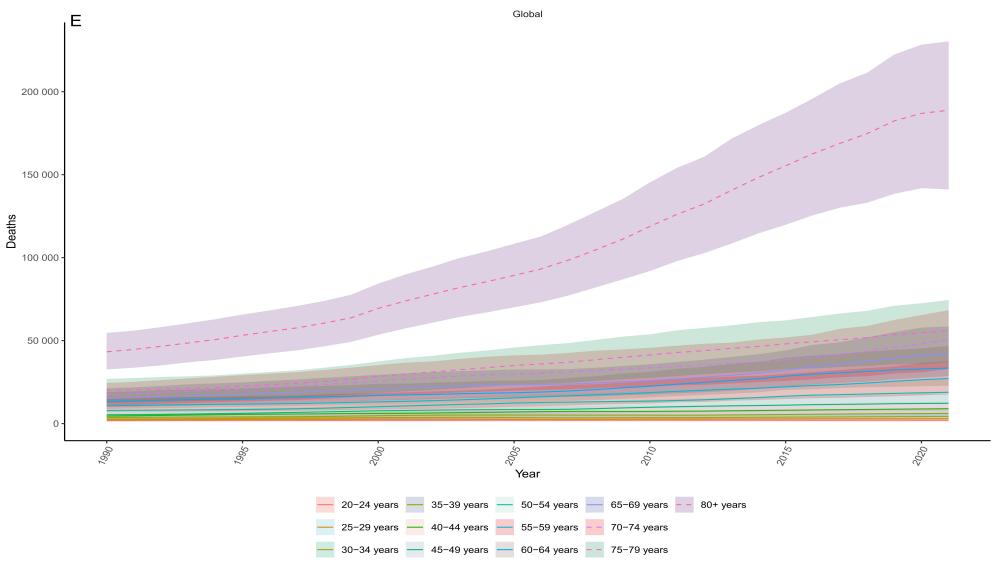


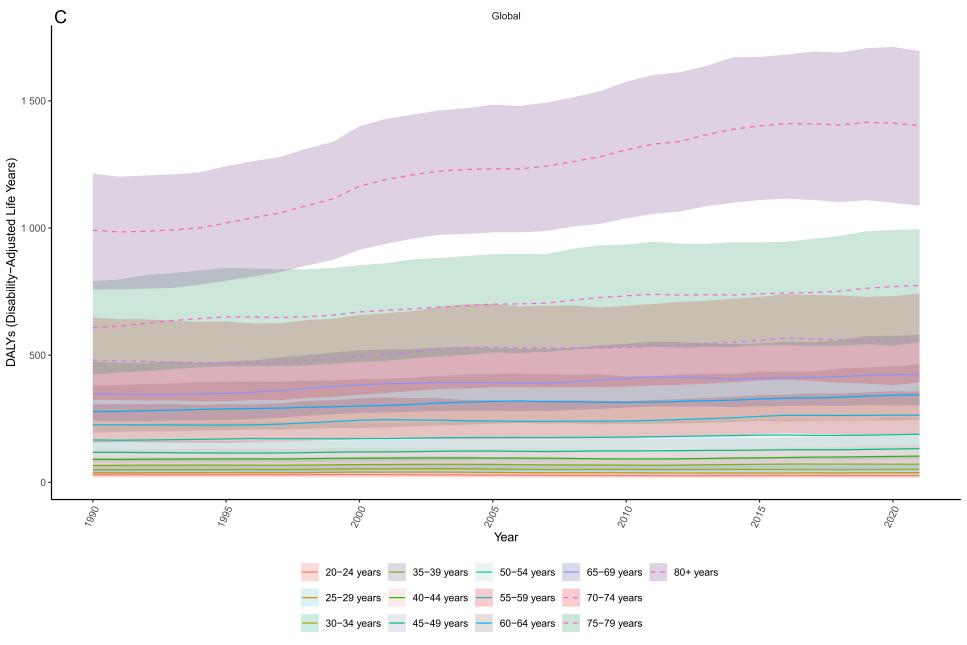

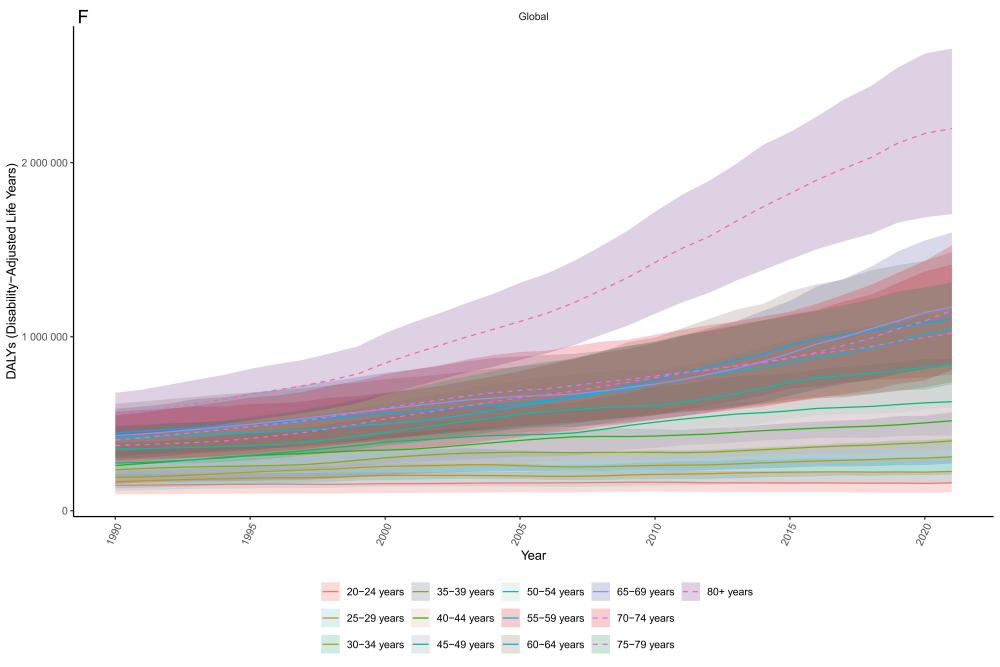


Supplementary Figure 1. Trends in chronic kidney disease due to hypertension incidence, deaths, and DALYs among adults aged 20 plus years from 1990 to 2021. (A) The age-standardized incidence rate. (B) The age-standardized death rate. (C) The age-standardized DALY rate. (D) Trends in incident cases. (E) Trends in death cases. (F) Trends in DALYs cases. DALYs, Disability adjusted life years.


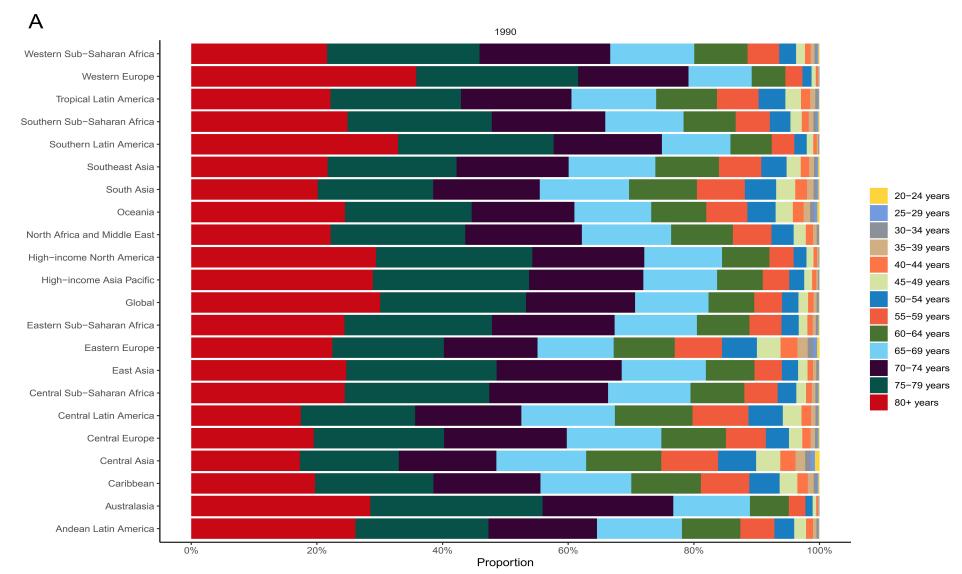

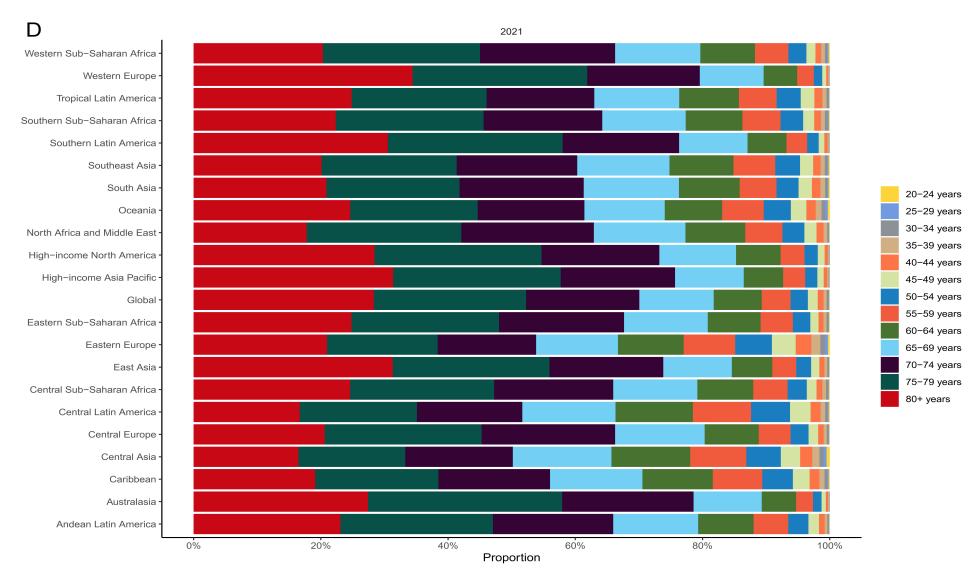


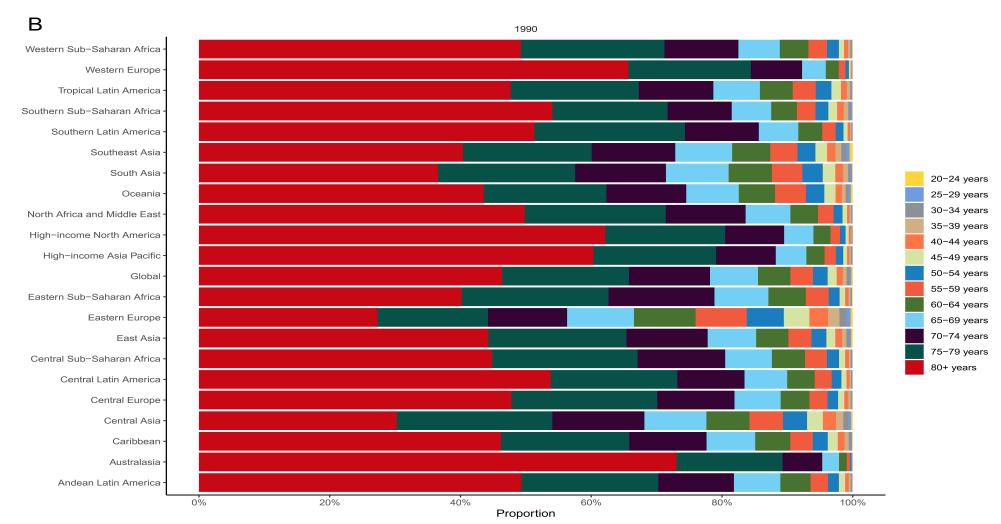

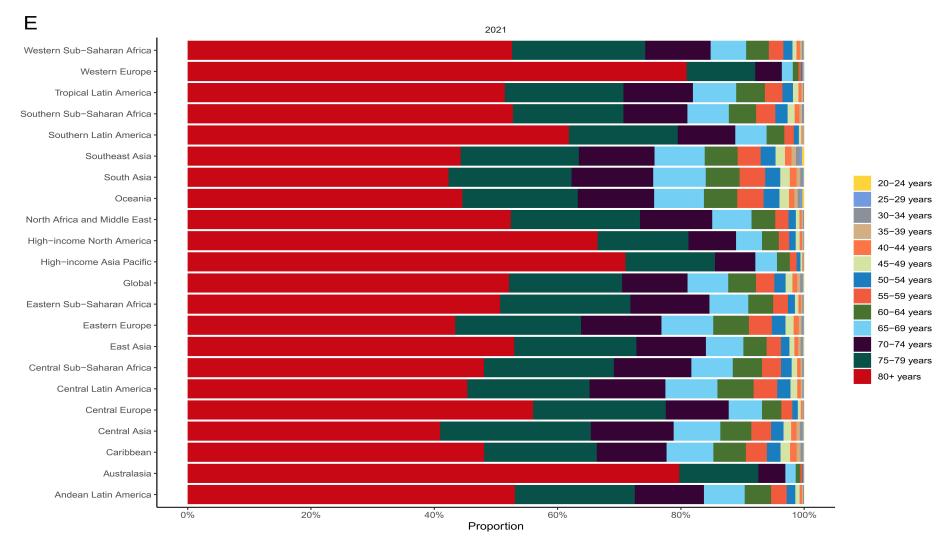


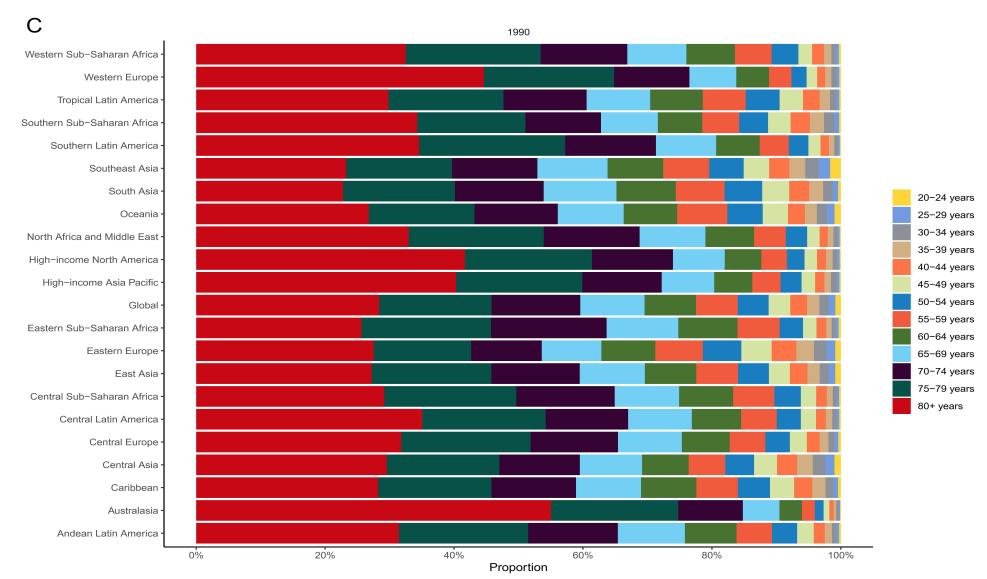

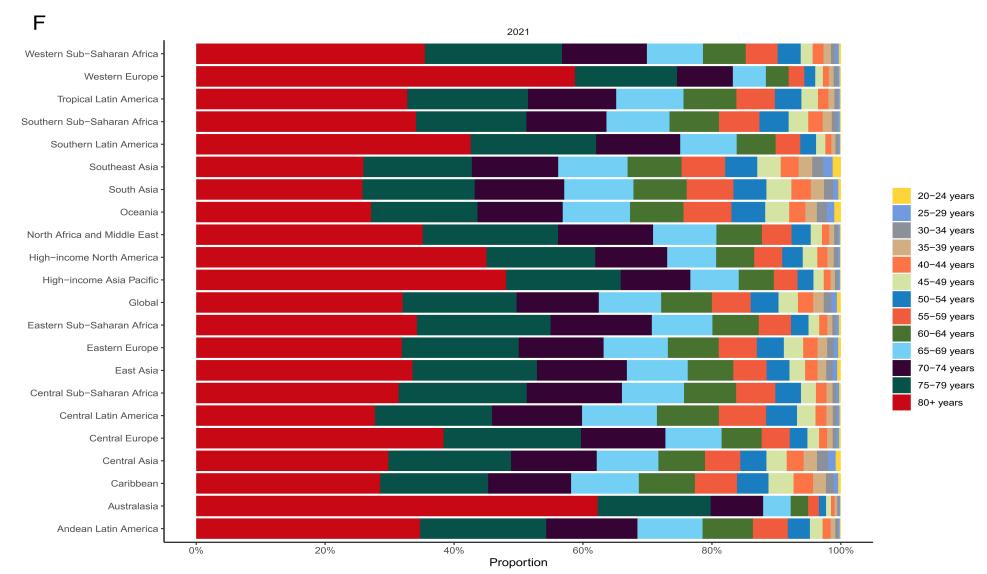


Supplementary Figure 2. Component ratios of age-standardized incidence, death, and DALY rates for different age groups of adults aged 20 years and older with chronic kidney disease due to hypertension, 1990 and 2021. (A) The incidence rate in 1990. (B) The death rate in 1990. (C) The DALY rate in 1990. (D) The incidence rate in 2021. (E) The death rate in 2021. (F) The DALY rate in 2021. DALY, Disability adjusted life years.


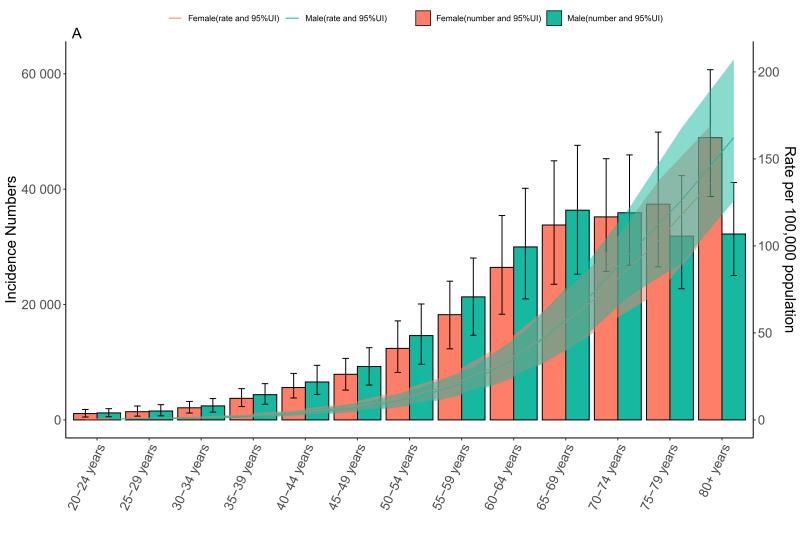

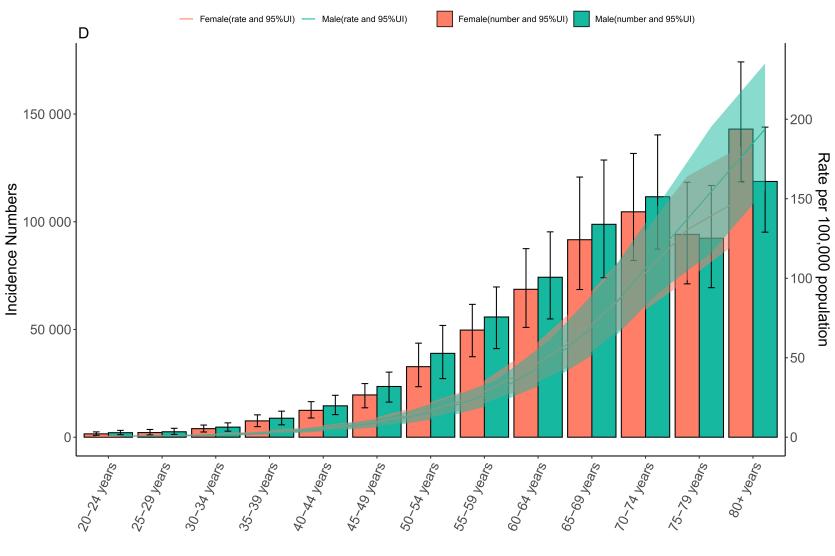


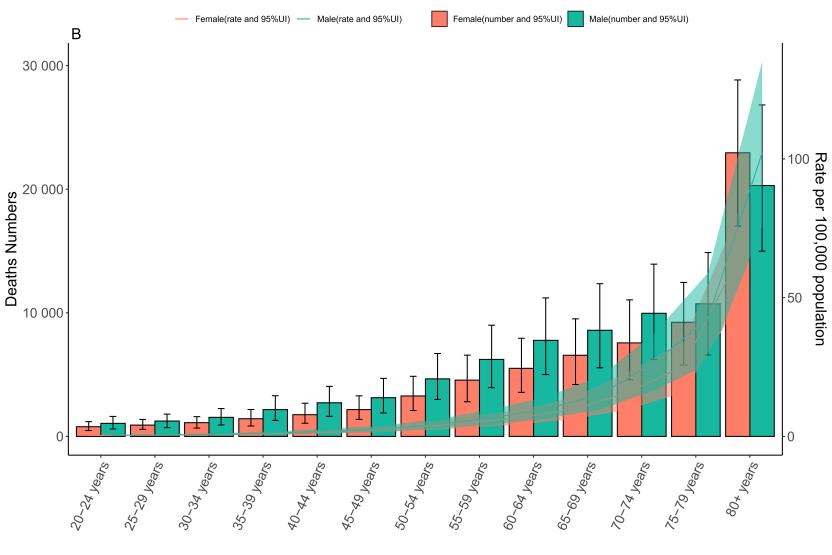

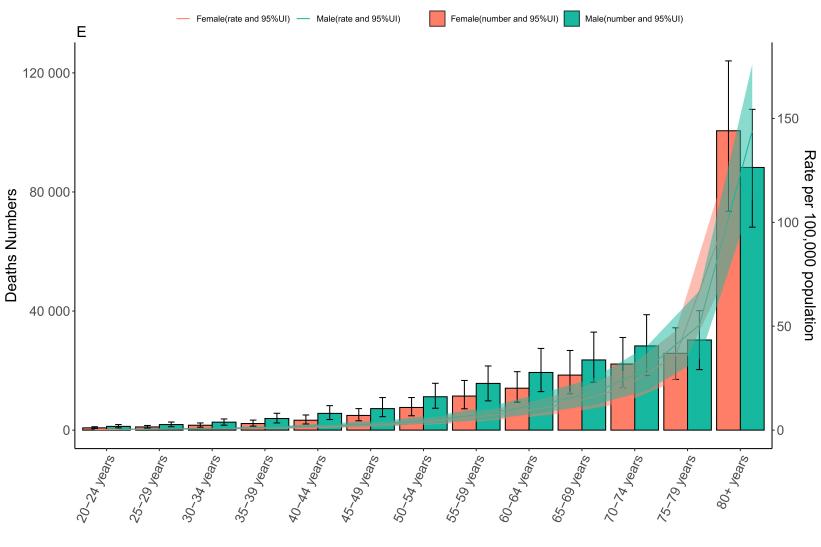


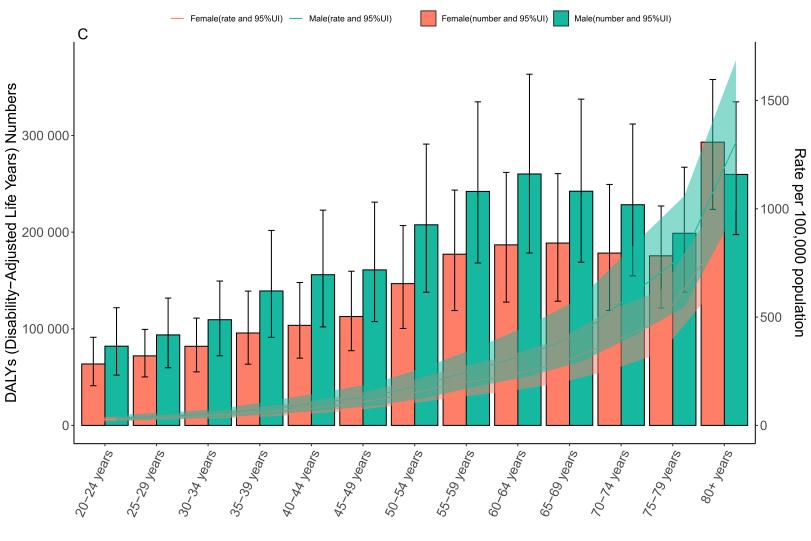

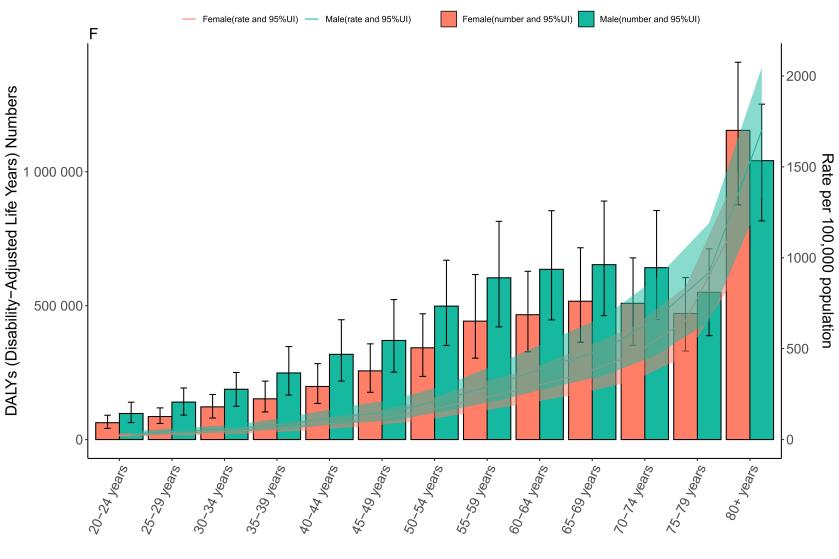


Supplementary Figure 3. Age specific burden of chronic kidney disease due to hypertension in adults aged 20 plus years in 1990 and 2021. (A) Trends in incident cases and age-standardized incidence rate in 1990. (B) Trends in death cases and age-standardized rate in 1990. (C) Trends in DALYs cases and age-standardized rate in 1990. (D) Trends in incident cases and age-standardized incidence rate in 2021. (E) Trends in death cases and age-standardized rate in 2021. (F) Trends in DALYs cases and age-standardized rate in 2021. DALYs, Disability adjusted life years.


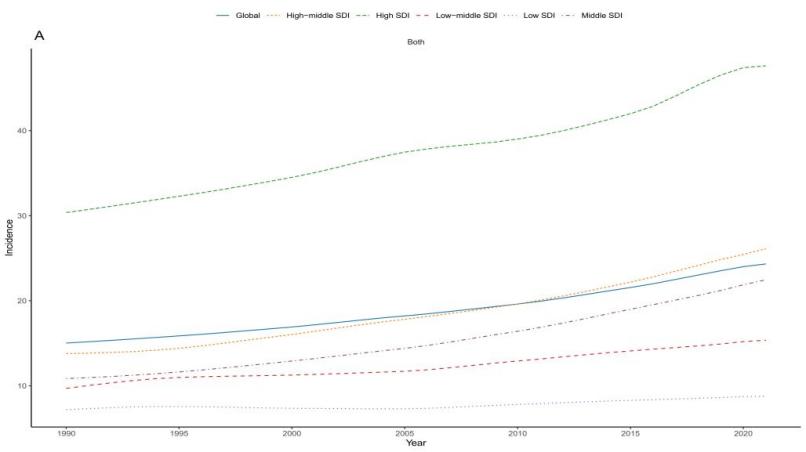

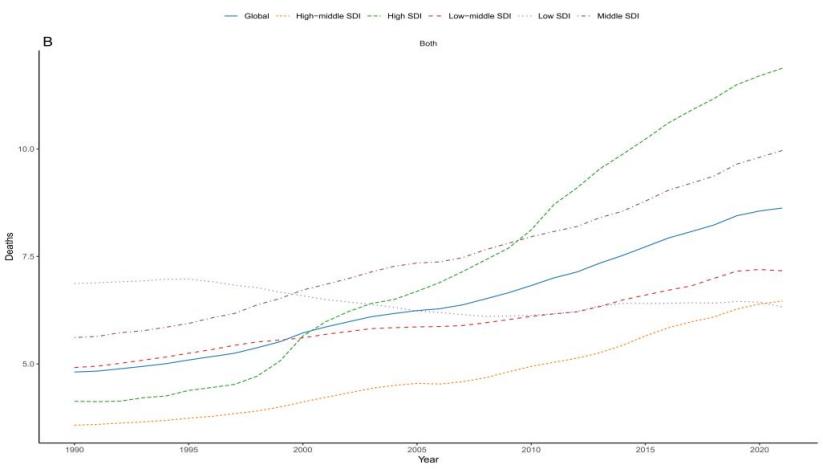


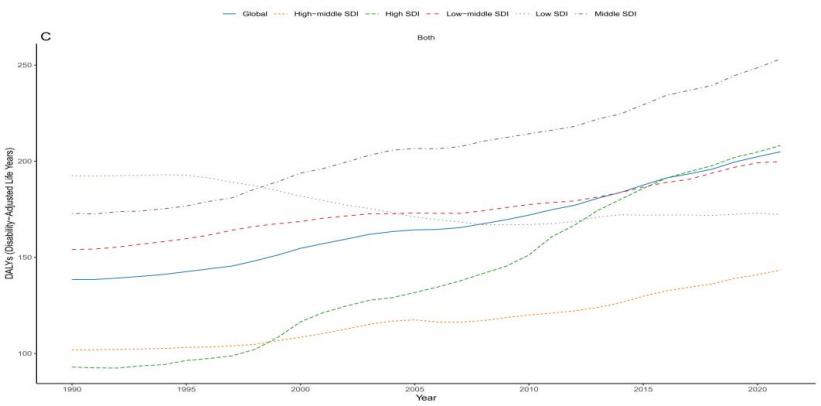


Supplementary Figure 4. Epidemiologic trends of age-standardized incidence, death, and DALY rates in global and 5 SDI regions of chronic kidney disease due to hypertension in adults aged 20 plus years between 1990 and 2021. (A) Incidence rate. (B) Death rate. (C) DALY rate. DALY, Disability adjusted life years. SDI, Sociodemographic Index.


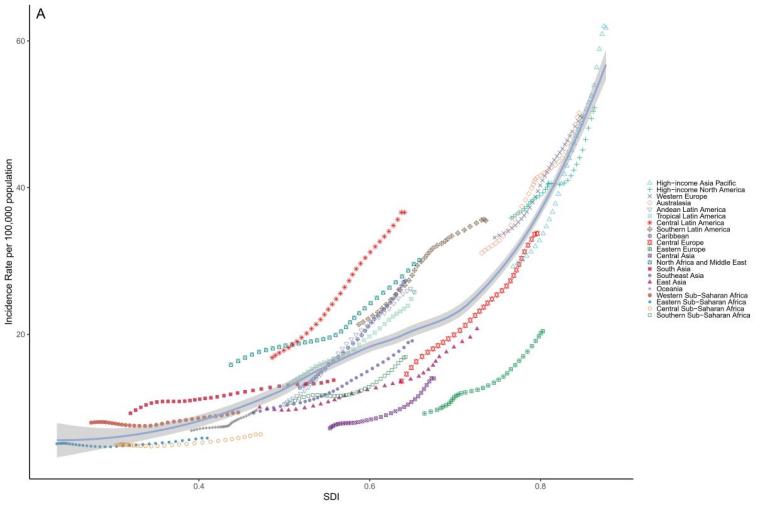

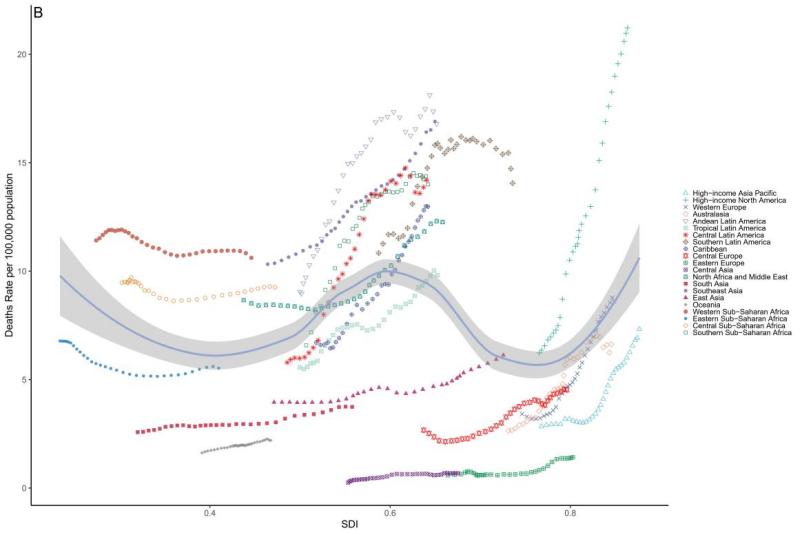


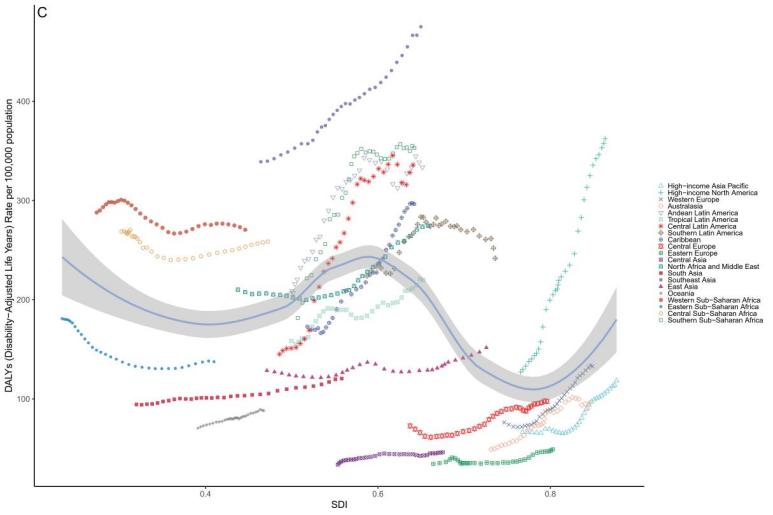

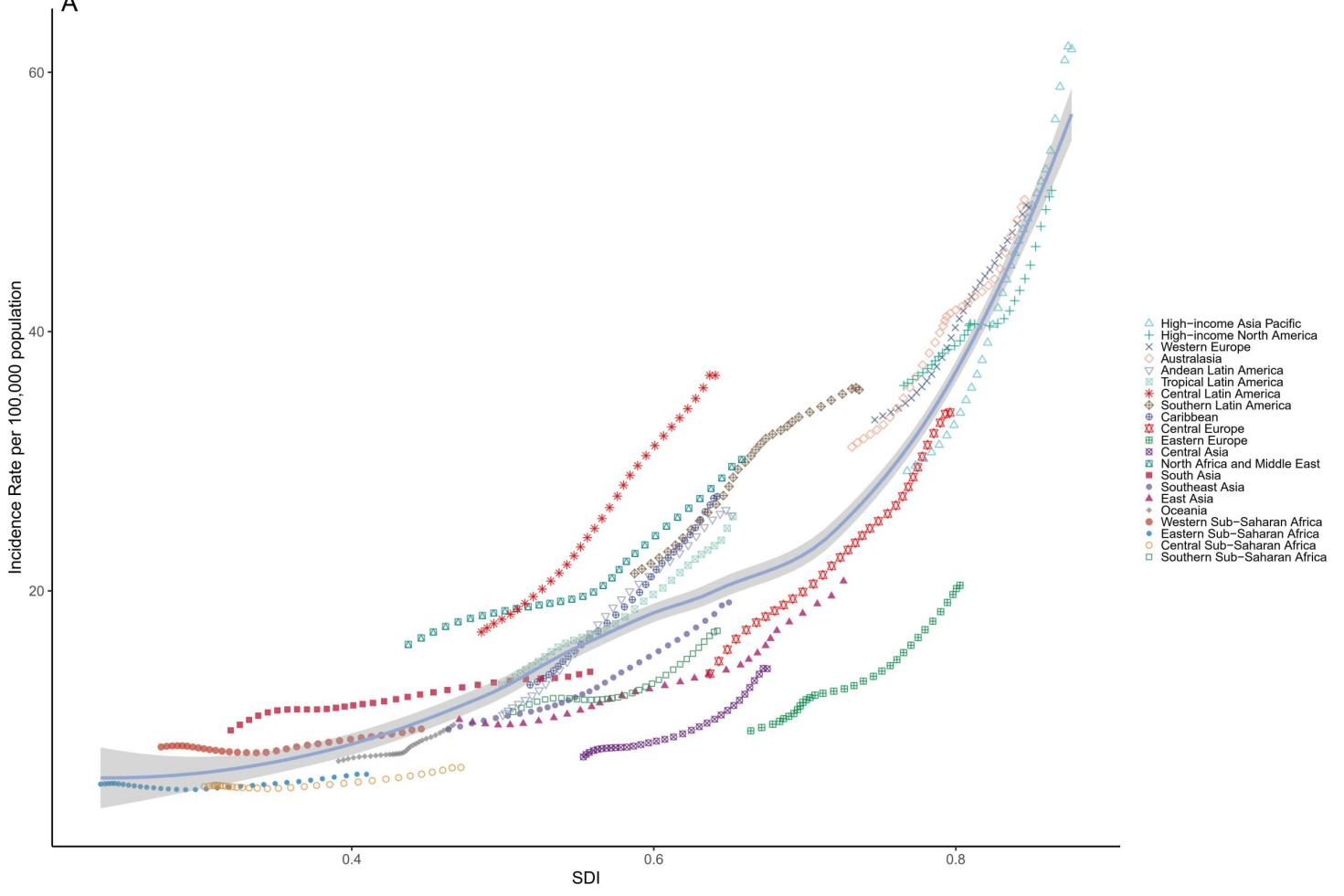


Supplementary Figure 5. Age-standardized Incidence, death, and DALY rates for chronic kidney disease due to hypertension in adults aged 20 plus years from 1990 to 2021. (A) Incidence rate. (B) Death rate. (C) DALY rate. DALY, Disability adjusted life years. SDI, Sociodemographic Index.


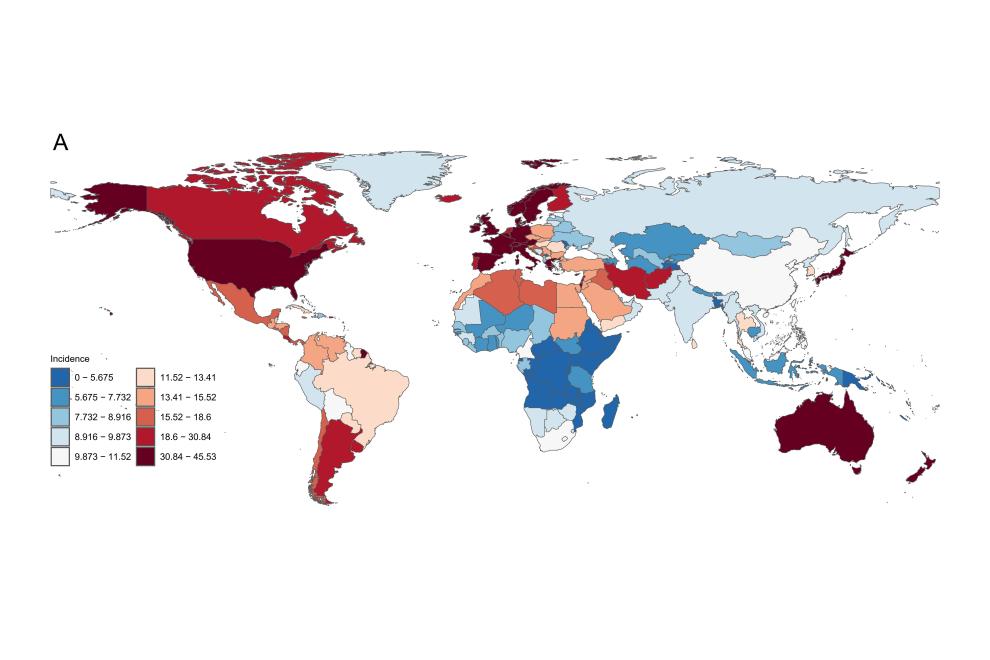

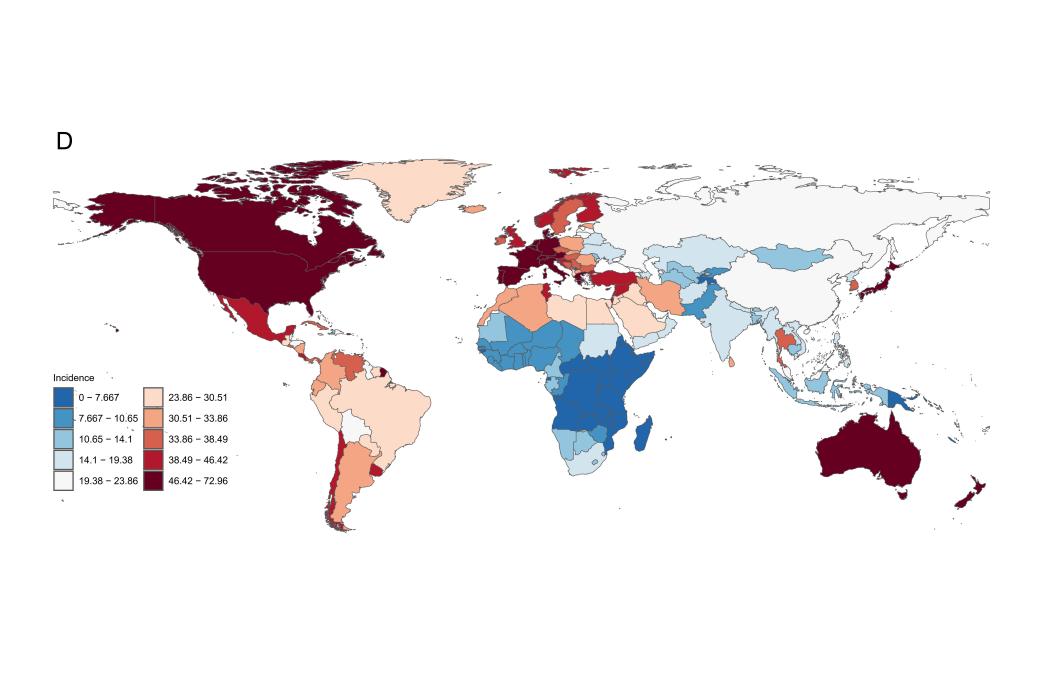


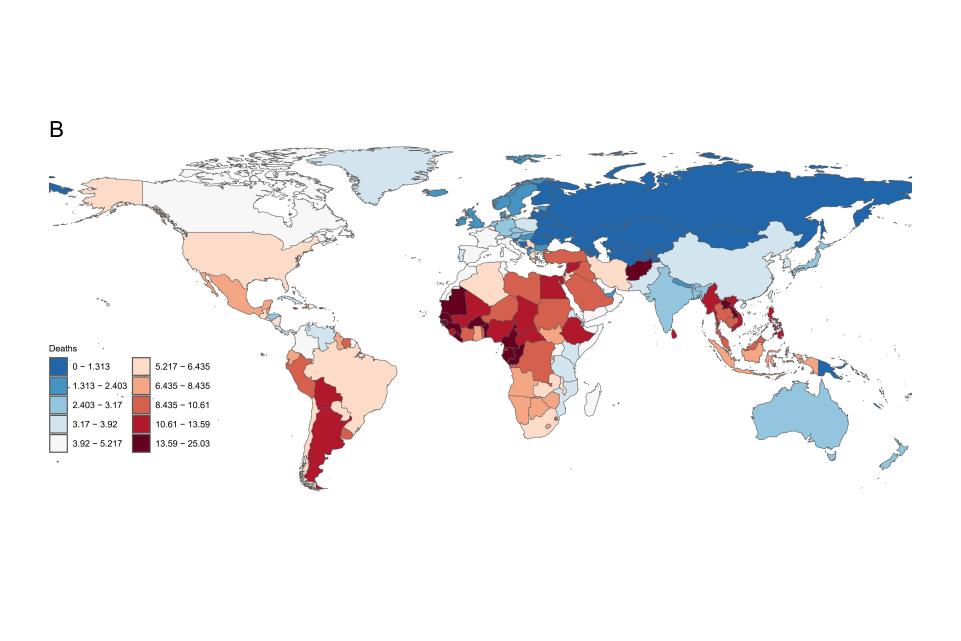

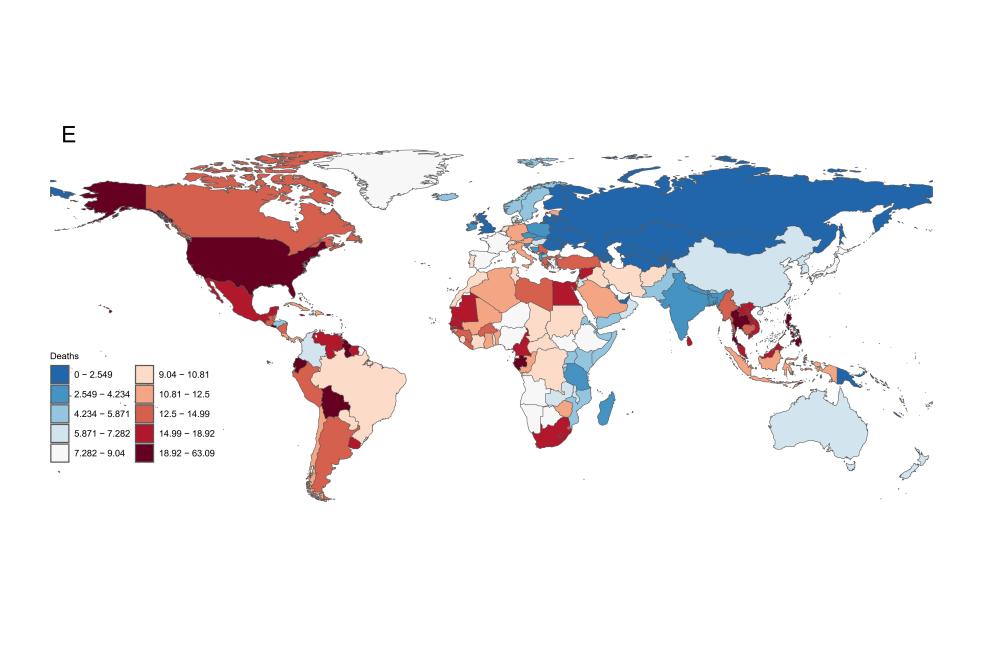


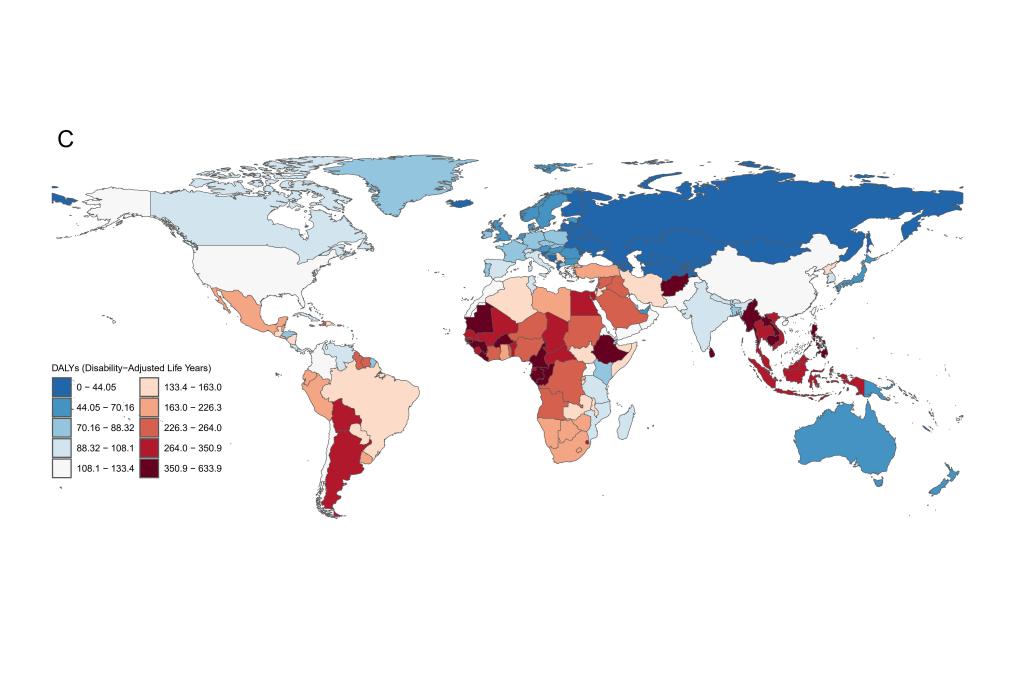

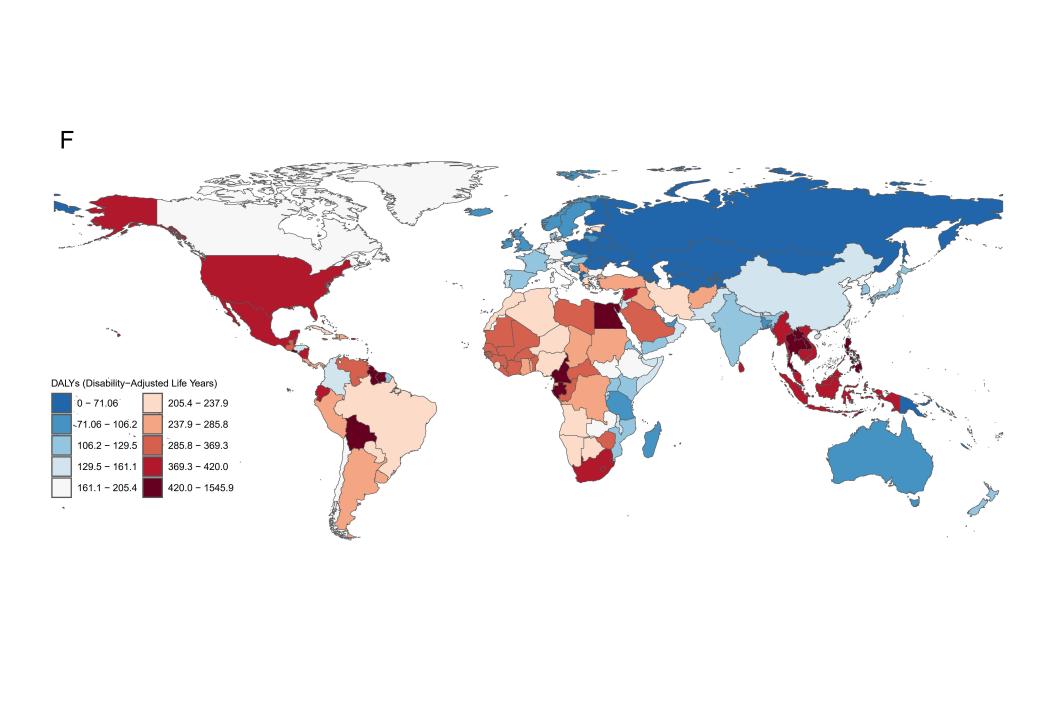


Supplementary Figure 6. The age-standardized incidence, death, and DALY rates of chronic kidney disease due to hypertension in adults aged 20 plus years in 204 Countries and Territories. (A) Incidence rate in 1990. (B) Death rate in 1990. (C) DALY rate in 1990. (D) Incidence rate in 2021. (E) Death rate in 2021. (F) DALY rate in 2021. DALY, Disability adjusted life years.
